# 2‑(Cyanomethyl)benzimidazole Derivatives as 1,3-Dicarbonyl Analogues for a Kinetically Controlled Diastereo- and Enantioselective Mannich-Type Reaction Catalyzed by Chiral Phosphoric Acid

**DOI:** 10.1021/acs.orglett.5c01478

**Published:** 2025-05-21

**Authors:** Haiting Ye, Linan Hou, Akihiro Takeda, Takuma Sato, Jyothi Yadav, Jun Kikuchi, Ming Bao, Masahiro Terada

**Affiliations:** † Department of Chemistry, Graduate School of Science, 13101 Tohoku University, Aoba-ku, Sendai 980-8578, Japan; ‡ State Key Laboratory of Fine Chemicals, 12399 Dalian University of Technology, Dalian 116023, China

## Abstract

An asymmetric Mannich-type reaction of *N*-protected 2-(cyanomethyl)­benzimidazoles with *N*-benzoyl imines was developed by using chiral phosphoric acid as a chiral Brønsted acid catalyst. Products having vicinal trisubstituted carbon stereogenic centers were formed in a highly diastereo- and enantioselective manner, even though one of the stereogenic centers had an active methine proton. Comprehensive control experiments revealed that high stereoselectivity was achieved through a kinetically controlled process.

## Graphical abstract

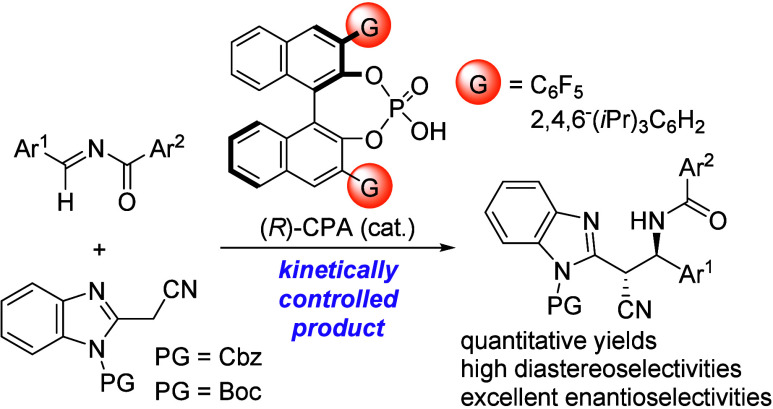

Benzimidazole, a representative azaarene chromophore, is widely found in natural products having pharmaceutical activities.^1^ An alkylazaarene derivative, such as 2-alkylbenzimidazole, exhibits unique chemical reactivity owing to the enamine tautomer, which possesses intrinsic nucleophilicity at the α-position.^2^ An electron-withdrawing group (EWG) is generally introduced at the α-position to enhance the acidity of the α-proton and the molecular complexity of the nucleophilic addition product.^3–5^ The EWG-mediated modification facilitates the generation of nucleophilic enamine. Hence, 2-alkylbenzimidazole with an EWG is regarded as an analogue of 1,3-dicarbonyl compounds, which are representative pronucleophiles in organic synthesis. Although 1,3-dicarbonyl compounds are synthetically useful pronucleophiles, one intrinsic drawback associated with the stereoselective reaction of 1,3-dicarbonyl compounds possessing an active methylene moiety with electrophiles (***El***) is the tautomerization of the addition products, which results in epimerization at the α-position^6^ and, in general, difficult control of the stereochemical outcome (Figure 1a). The 2-alkylazaarene derivatives, including benzimidazole analogues, also encounter similar issues. To circumvent this inherent problem, 2-alkylazaarene derivatives having an additional substituent at the α-position are employed. The stereocontrol of a generated tetrasubstituted stereogenic center has drawn much interest as a challenging issue.^5^

**Figure 1:**
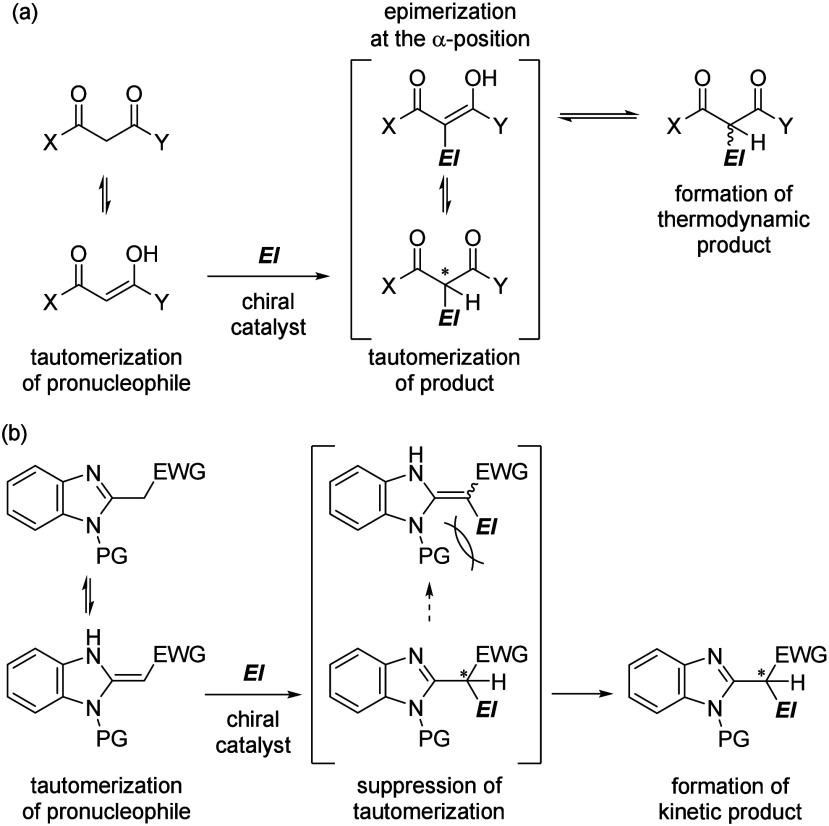
Tautomerization of a pronucleophile and formation of a nucleophilic addition product. (a) 1,3-Dicarbonyl compound. (b) 2-Alkylbenzimidazole derivative having an EWG and a PG.

In our continuous efforts to utilize the benzimidazole chromophore in stereoselective reactions,^7^ we envisioned the fascinating effects of an additional electron-withdrawing substituent, such as a common protective group (PG), for example, a *tert*-butoxycarbonyl (Boc) or a benzyloxycarbonyl (Cbz) group, at the nitrogen atom of the 2-alkylbenzimidazole unit (Figure 1b). Introducing PG enhances the acidity of the α-proton, facilitating the tautomerization of the 2-alkylbenzimidazole pronucleophile^8^ and leading to an intriguing steric effect estimated in the nucleophilic addition product. The steric congestion, which originates from A^(1,3)^ strain between ***El*** (or EWG) and PG around the tetrasubstituted double bond of the enamine tautomer, will suppress the tautomerization of the addition product.^6^

Leveraging the anticipated properties of the 2-alkylbenzimidazole derivative as a pronucleophile, we postulated that a kinetically controlled nucleophilic addition reaction would be feasible. In order to validate the above postulate, we adopted the Mannich-type reaction of *N*-protected 2-(cyanomethyl)­benzimidazoles **1** with *N*-benzoyl imines **2** using binaphthol-derived chiral phosphoric acid (CPA) as a chiral Brønsted acid catalyst (Scheme 1).^9–11^ Herein, we report that the stereoselective Mannich-type reaction using CPA (*R*)-**3** affords enantioenriched 2-alkylbenzimidazole derivatives **4** having vicinal trisubstituted carbon stereogenic centers in a highly diastereo- and enantioselective manner. Comprehensive control experiments revealed that high stereoselectivity was achieved through a kinetically controlled process.

**Scheme 1:**
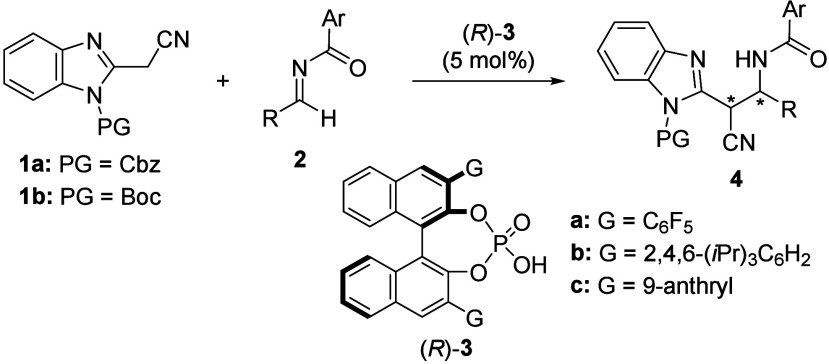
Diastereo- and Enantioselective Mannich-Type Reaction of 2-(Cyanomethyl)­benzimidazoles **1** with *N*-Benzoyl Imines **2** Catalyzed by CPA (*R*)-**3**

We initiated our investigation by the reaction of 2-(cyanomethyl)­benzimidazole **1a**, which has a Cbz group on the nitrogen atom, with *N*-benzoyl imine **2a** at 0 °C for 4 h under the influence of CPA (*R*)-**3a** having pentafluorophenyl substituents (Table 1). When the reaction was conducted in toluene (entry 1) or dichloromethane (entry 2), these commonly used solvents^10^ resulted in the quantitative formation of the desired **4aa** in a highly enantioselective manner, albeit with low diastereoselectivity. In contrast, the use of THF, which is rarely employed in CPA-catalyzed reactions,^10^ improved the diastereoselectivity without any detrimental effect on the enantioselectivity (entry 3). Further screening of the solvents showed that high diastereoselectivity (94/6) was achieved in Et_2_O, affording **4aa** quantitatively with a slight improvement in enantioselectivity (97% ee) (entry 4). The effect of substituents at the 3,3′-positions of CPA was further investigated using (*R*)-**3b** and (*R*)-**3c** having bulky 2,4,6-triisopropylphenyl and 9-anthryl substituents, respectively (entries 5 and 6). Both catalysts exhibited excellent enantioselectivities (98% ee), although the diastereoselectivity was slightly influenced by the substituent on CPA. The reaction of **1b**, which has a Boc protective group, also proceeded smoothly using catalysts **3a** and **3b**, but the diastereoselectivity decreased (entries 7 and 8). In addition, the enantioselectivity was dependent on catalysts **3a** and **3b**; the use of **3a** resulted in a higher enantioselectivity than the use of **3b**, unlike the case of Cbz-protected **1a**.

**Table 1:**
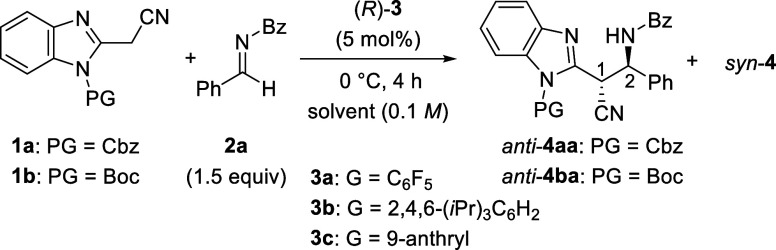
Optimization of the Reaction Conditions^a^

entry	**1**	(*R*)-**3**	solvent	*anti*/*syn*^b^,^c^	ee (%)^d^
1	**1a**	(*R*)-**3a**	toluene	60/40^e^	96
2	**1a**	(*R*)-**3a**	CH_2_Cl_2_	64/36^f^	95
3	**1a**	(*R*)-**3a**	THF	85/15	95
4	**1a**	(*R*)-**3a**	Et_2_O	94/6	97
5	**1a**	(*R*)-**3b**	Et_2_O	95/5	98
6	**1a**	(*R*)-**3c**	Et_2_O	91/9	98
7	**1b**	(*R*)-**3a**	Et_2_O	84/16	95
8	**1b**	(*R*)-**3b**	Et_2_O	83/17	88

a Reaction conditions: **1** (0.20 mmol), **2a** (0.30 mmol, 1.5 equiv), and (*R*)-**3** (5 mol %, 0.01 mmol) in the indicated solvent (2 mL, 0.1 *M*) under a nitrogen atmosphere unless otherwise specified.

b A diastereomeric mixture of **4** was obtained quantitatively in all cases.

c The diastereomeric ratio was determined by ^1^H NMR measurements after short path silica-gel column chromatography.

d The enantiomeric excess of the major *anti*-diastereoisomer was determined by HPLC analysis using a chiral stationary phase column.

e A 93% ee for the *syn*-isomer.

f A 60% ee for the *syn*-isomer.

In order to verify the formation of a kinetically controlled product, we monitored the stereoselectivities during the reaction of **1a** with **2a** (1.5 equiv) in the presence of (*R*)-**3a** and (*R*)-**3b** (Scheme 2a). After confirmation of the complete consumption of **1a** at 0 °C for 4 h, the reaction temperature was increased to room temperature, and the reaction was continued for an additional 44 h. Neither the enantioselectivity nor the diastereoselectivity was changed by using less acidic (*R*)-**3b**. Hence, the tautomerization of formed **4aa** seemed to be completely suppressed under the present reaction conditions. In contrast, the use of more acidic (*R*)-**3a** resulted in a significant decrease in the diastereoselectivity to 65/35, clearly indicating that epimerization of **4aa** occurred. More curiously, a non-negligible decrease in enantioselectivity was also observed in both diastereoisomers, strongly suggesting that the epimerization proceeds not only at the α-position of **4aa** but also at the β-position. Consequently, a retro reaction was suspected because tautomerization is responsible only for epimerization at the α-position. Therefore, we performed a control experiment using a 1/1 mixture of product **4aa** and imine **2b** in the presence of (*R*)-**3a** at room temperature for 6 h (Scheme 2b). As expected, cross product **4ab** was formed in 27% yield with 73% recovery of the starting product **4aa**. Hence, we confirmed that the retro reaction occurred under the present acidic conditions. It should be emphasized that these epimerization processes were suppressed at 0 °C during the initial 4 h, and hence, initially formed **4aa** at 0 °C is regarded as a kinetically controlled product, as postulated (Figure 1).

**Scheme 2:**
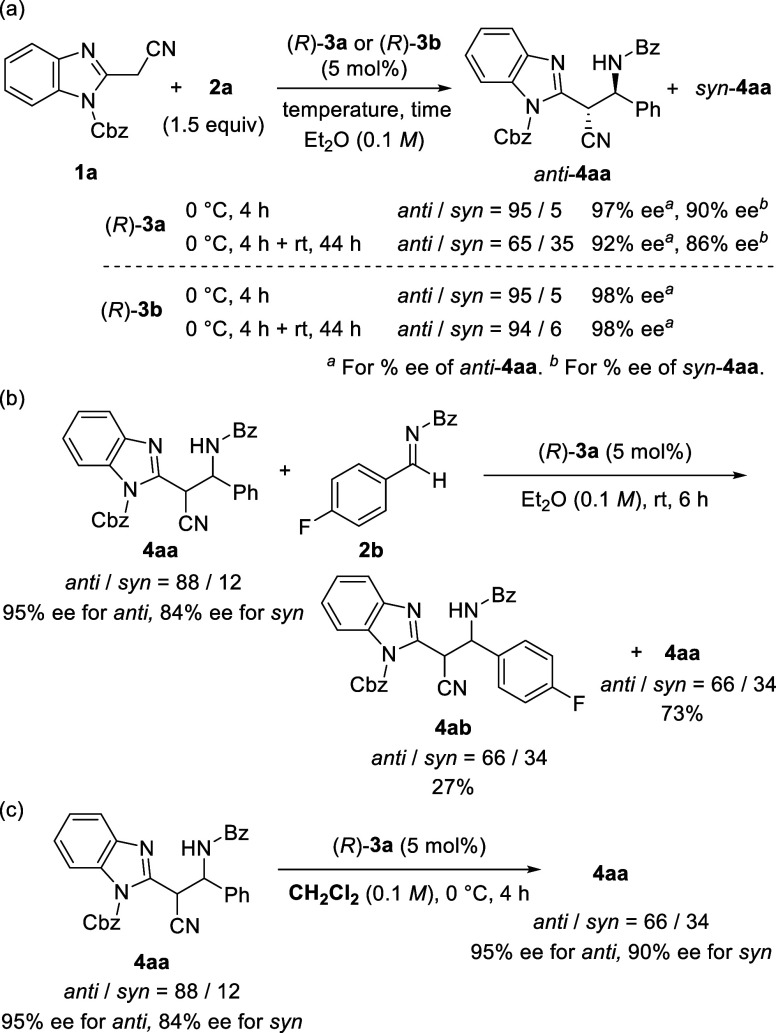
Control Experiments: (a) Monitoring the Reaction, (b) Retro Reaction, and (c) Solvent Effect

As shown in Table 1, a unique solvent effect was observed in the present reaction. Commonly used solvents, such as toluene and dichloromethane, afforded product **4aa** with low diastereoselectivity, despite the high enantioselectivity (entries 1 and 2). We performed a control experiment in dichloromethane using enantiomerically enriched product **4aa** [*anti*/*syn* = 88 (95% ee)/12 (84% ee)] and (*R*)-**3a** to confirm whether a kinetically controlled product could be formed in this commonly used solvent (Scheme 2c). The diastereoselectivity was markedly decreased to a 66/34 *anti*/*syn* ratio, even at 0 °C for 4 h. In contrast, high enantioselectivities were maintained for both diastereoisomers; more importantly, an enhancement of the enantioselectivity from 84% to 90% ee was observed for minor *syn*-**4aa**. These results suggest that the epimerization at only the α-position of **4aa**, namely, tautomerization, occurred. The enhanced enantioselectivity of *syn*-**4aa** stemmed from major *anti*-**4aa** having higher enantioselectivity (95% ee) than that of initial *syn*-**4aa** (84% ee). In addition, the retro reaction would be excluded from the present epimerization process. On the basis of the above control experiments, we conclude that the use of Et_2_O is the key to suppressing the tautomerization efficiently.

The optimal conditions for the formation of the kinetically controlled product were identified. Both catalysts (*R*)-**3a** and (*R*)-**3b** performed efficiently in the reaction of Cbz-protected substrate **1a** in Et_2_O. The scope of the reaction was demonstrated using a series of aryl-substituted imines **2** under the influence of (*R*)-**3a** or (*R*)-**3b** (see the Supporting Information for details). As shown in Table 2, a range of imines **2** are suitable for the present reaction, affording desired products **4** quantitatively with high diastereo- and enantioselectivities. The reaction of *N*-benzoyl imines **2** having a substituent at the *para* position of the aryl ring, **2b**–**2f**, proceeded well with high stereoselectivities, regardless of the electronic properties of the aryl ring (entries 1–7). The absolute configuration of **4ac** was determined to be 1*R*,2*R*, indicating *anti*-relative stereochemistry, as the major stereoisomer using (*R*)-**3a** [and also (*R*)-**3b**] through single-crystal X-ray analysis of *syn*-**4ac** after epimerization at the C1 position of *anti*-**4ac** (see the Supporting Information for details). In addition, the reaction on a gram scale (**1a**, 2.0 mmol; **2d**, 3.0 mmol) proceeded smoothly without any undesirable effects on the stereoselectivity, affording **4ad** quantitatively, even with a reduced loading of (*R*)-**3a** of 2.5 mol % (entry 5). Aryl rings having a substituent at the *meta* or *ortho* position, **2g**–**2i**, also underwent the reaction smoothly with good stereoselectivities (entries 8–10). The reaction of **2k**, which has a heteroaryl group, 2-thiophenyl, resulted in a decrease in stereoselectivity (entry 12). In contrast, the reaction of 2-naphthyl substrate **2j** proceeded well using (*R*)-**3b** with excellent stereoselectivity (entry 11). The *N*-benzoyl group was comparable to those of other aryl rings; the use of 4-bromophenyl **2l** and 4-toluyl **2m** afforded **4al** and **4am**, respectively, in a highly enantioselective manner, albeit with a slight decrease in diastereoselectivity (entries 13 and 14). In contrast, the use of *N*-benzoyl imine **2n** having an aliphatic substituent retarded the reaction markedly (entry 15), affording **4an** in moderate yield with a significant decrease in enantioselectivity, albeit with relatively high diastereoselectivity. Introducing an ester moiety instead of a cyano group (entry 16) markedly decreased the diastereoselectivity, although the major diastereoisomer exhibited fairly good enantioselectivity. This result emphasizes the crucial role played by a cyano group in achieving high diastereoselectivity.

**Table 2:**
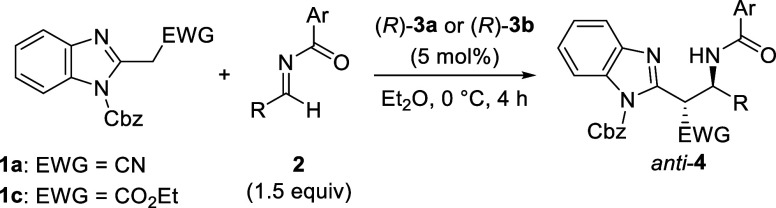
Scope of Substrates Using (*R*)-**3a** or (*R*)-**3b** ^a^

entry	(*R*)-**3**	**2**: R, Ar	**4**	*anti*/*syn*^b^	ee (%)^c^
1	**3b**	**2b**: 4-FC_6_H_4_, Ph	**4ab**	>95/5	98
2	**3b**	**2c**: 4-ClC_6_H_4_, Ph	**4ac**	>95/5	98
3	**3b**	**2d**: 4-BrC_6_H_4_, Ph	**4ad**	>95/5	98
4	**3a**	**2d**	**4ad**	>95/5	98
5^d^	**3a**	**2d**	**4ad**	>95/5	98
6	**3a**	**2e**: 4-CF_3_C_6_H_4_, Ph	**4ae**	>95/5	99
7	**3a**	**2f**: 4-MeC_6_H_4_, Ph	**4af**	94/6	99
8	**3b**	**2g**: 3-MeC_6_H_4_, Ph	**4ag**	>95/5	91
9	**3a**	**2h**: 3-FC_6_H_4_, Ph	**4ah**	91/9	92
10^e^	**3a**	**2i**: 2-FC_6_H_4_, Ph	**4ai**	>95/5	99
11^e^	**3b**	**2j**: 2-naphthyl, Ph	**4aj**	>95/5	98
12	**3b**	**2k**: 2-thiophenyl, Ph	**4ak**	83/17	92
13	**3b**	**2l**: Ph, 4-BrC_6_H_4_	**4al**	94/6	99
14	**3b**	**2m**: Ph, 4-MeC_6_H_4_	**4am**	93/7	95
15	**3a**	**2n**: cyclohexyl, Ph	**4an** ^f^	87/13	29, 18
16^g^	**3a**	**2a**: Ph, Ph	**4ca**	73/27	90, 81

a Reaction conditions: **1a** (0.20 mmol), **2** (0.30 mmol), and (*R*)-**3** (5 mol %) in Et_2_O (2 mL, 0.1 *M*) under a nitrogen atmosphere at 0 °C for 4 h unless otherwise specified.

b Diastereomeric mixtures were formed quantitatively, and the diastereomeric ratio (*anti*/*syn*) was determined by ^1^H NMR measurements after short path silica-gel column chromatography.

c The enantiomeric excess was determined by HPLC analysis using a chiral stationary phase column.

d Gram-scale experiment: **1a** (2.0 mmol), **2d** (3.0 mmol), and (*R*)-**3a** (2.5 mol %, 0.05 mmol) in Et_2_O (20 mL, 0.1 *M*).

e For 12 h.

f A 63% combined yield of diastereoisomers.

g Use **1c** (0.20 mmol) instead of **1a** for 16 h.

Finally, to gain insight into the reaction profile, we performed density functional theory (DFT) calculations^12^ of the reaction of **1a** with **2a** catalyzed by (*R*)-**3a**. Focusing on the (1*R*,2*R*)-**4aa** stereoisomer obtained as the major product, transition state (TS) analysis of C–C bond formation (**TS**_**CRR**_), namely, the Mannich-type reaction, and the tautomerization (**TS**_**TU**_) steps was conducted to verify the progress of the retro reaction and the formation of a tautomer. The structural optimization of TSs was performed, taking into account the solvent effect of diethyl ether and using CPCM­(ether)/ωB97X-D/def2-SVP, followed by a further single-point energy calculation of these optimized structures at the SMD­(ether)/ωB97M-V/def2-SVPD level of theory. The energy diagram for the C–C bond formation and tautomerization steps is shown in Figure 2 (the three-dimensional structures are presented in the Supporting Information). We confirmed that the activation energy for C–C bond formation that gives the product through **TS**_**CRR**_ was sufficiently small (Δ*G* ^⧧^ = 5.8 kcal/mol), affording (1*R*,2*R*)-**4aa** predominantly (blue line). More importantly, the energy barrier of the backward reaction was not markedly high (Δ*G* ^⧧^ = 17.2 kcal/mol), allowing the retro reaction to proceed at room temperature. On the other hand, tautomerization (**TS**_**TU**_) had a relatively low energy barrier. Still, it was higher (ΔΔ*G* ^⧧^ = 1.4 kcal/mol) than that of the C–C bond formation step for giving (1*R*,2*R*)-**4aa**, in good agreement with the experimental result that the tautomerization was suppressed at 0 °C. High enantioselectivity was also confirmed by the TS analysis of the pathway to 1*S*,2*S*-**4aa** (**TS**_**CSS**_) (orange line), which is ΔΔ*G* ^⧧^ = 1.8 kcal/mol higher than the formation of (1*R*,2*R*)-**4aa** (**TS**_**CRR**_).

**Figure 2:**
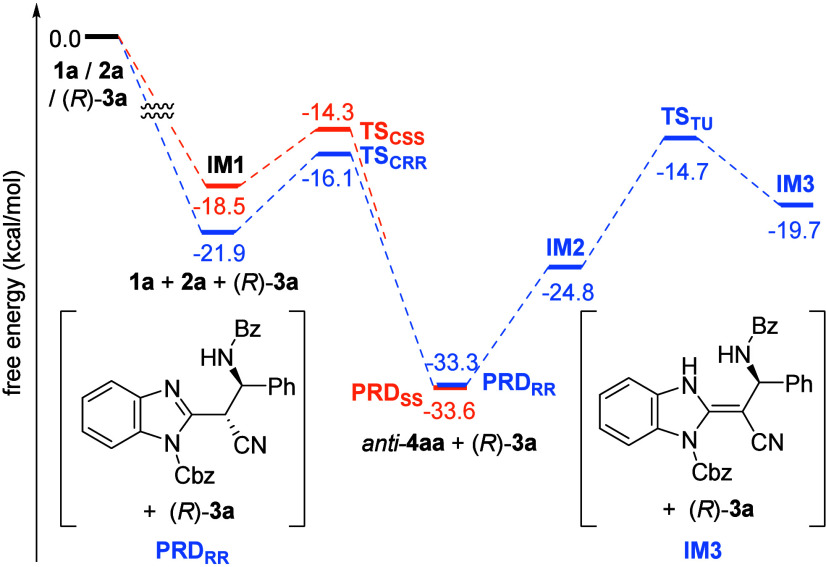
Energy diagram of the reaction of **1a** with **2a** catalyzed by (*R*)-**3a**. The relative free energy (kcal/mol) of the sum of **1a**, **2a**, and (*R*)-**3a** is set to zero.

In conclusion, we demonstrated an asymmetric Mannich-type reaction of *N*-protected 2-(cyanomethyl)­benzimidazoles with *N*-benzoyl imines catalyzed by chiral phosphoric acid. Taking advantage of the structural features of *N*-protected 2-(cyanomethyl)­benzimidazoles, vicinal trisubstituted carbon stereogenic centers were kinetically controlled in a highly diastereo- and enantioselective manner, even though one of the two stereogenic centers had an active methine proton. DFT calculations characterized the present reaction profile well, which involved not only tautomerization but also retroreaction, both of which were observed under specific reaction conditions. Further studies of other reaction designs utilizing the structural properties of *N*-protected 2-alkylbenzimidazole derivatives are in progress in our laboratory.

## Supplementary Material



## Data Availability

The data underlying this study are available in the published article and its Supporting Information.

## Notes

Experimental details, characterization data, theoretical studies, and ^1^H, ^13^C, and ^19^F NMR spectra and HPLC charts of isolated compounds (PDF)

## ∇

J.K.: Graduate School of Pharmaceutical Science, Tohoku University, Aoba-ku, Sendai 980-8578, Japan
