# Supplementary material for: 2‑(Cyanomethyl)benzimidazole Derivatives as 1,3-Dicarbonyl Analogues for a Kinetically Controlled Diastereo- and Enantioselective Mannich-Type Reaction Catalyzed by Chiral Phosphoric Acid
Source: Org Lett. 2025 May 21;27(22):5720–5. doi: 10.1021/acs.orglett.5c01478 (PMC12150310; doi:10.1021/acs.orglett.5c01478)
Supplement: Supplementary file 1 [file ol5c01478_si_001.pdf]

Supporting Information

2-(Cyanomethyl)benzimidazole Derivatives as 1,3-Dicarbonyl Analogs for Kinetically Controlled Diastereo- and Enantioselective Mannich-Type Reaction Catalyzed by Chiral Phosphoric Acid

Haiting Ye,[a] Linan Hou,[a,b] Akihiro Takeda,[a] Takuma Sato,[a] Jyothi Yadav,[a] Jun Kikuchi,[a] Ming Bao,[b] Masahiro Terada[a]*

e-mail: mterada@tohoku.ac.jp

[a] Department of Chemistry, Graduate School of Science, Tohoku University, Aoba-ku, Sendai 980-8578, Japan

[b]State Key Laboratory of Fine Chemicals, Dalian University of Technology, Dalian 116023, China

Contents

1. **General Information……S2**
2. **Preparation of Substrates……S3**
3. **Initial Screening of Diastereo- and Enantioselective Mannich-type Reaction of Benzimidazoles with Imines…S5**
4. **Scope of Diastereo- and Enantioselective Mannich-type Reaction of Benzimidazoles with Imines……S9**
5. **Gram Scale Experiment……S15**
6. **Control Experiments……S16**
7. **Determination of Relative and Absolute Configurations……S17**
8. **Theoretical Studies……S21**
9. **NMR Spectra……S37**
10. **HPLC Charts……S59**

1. General Information

All reactions were carried out under a nitrogen atmosphere in flame-dried glassware. Dichloromethane (CH_2Cl_2), tetrahydrofuran (THF), diethyl ether (Et_2O), and toluene were supplied from KANTO Chemical Co., Inc., as a “dehydrated solvent system”. Other solvents and reagents were purchased from commercial suppliers and used without further purification. Purification of reaction products was carried out by flash column chromatography using silica gel 60 N (Merck 40 - 63 μm). Analytical thin layer chromatography (TLC) was performed on Merck precoated TLC plates (silica gel 60 GF 254, 0.25 mm). ^{1}H NMR spectra were recorded on a JEOL ECA-600 (600 MHz) spectrometer and a JEOL ECA-400 (400 MHz) spectrometer. Chemical shifts are reported in ppm from tetramethylsilane or solvent resonance as the internal standard ($CDCl_3$, 7.26 ppm; TMS, 0.00 ppm). ^{13}C NMR spectra were recorded on a JEOL ECA-600 (151 MHz) spectrometer a JEOL ECA-400 (100 MHz) spectrometer with complete proton decoupling. Chemical shifts are reported in ppm from the solvent resonance as the internal standard ($CDCl_3$: 77.0 ppm). ^{19}F NMR spectra were recorded on a JEOL JNM-ECA600 (565 MHz) spectrometer JEOL JNM-ECA400 (377 MHz) spectrometer with complete proton decoupling. Chemical shifts are reported in ppm from the $C_6H_5CF_3$ (-67.2 ppm) resonance as the external standard. Infrared spectra were recorded on a Jasco FT/IR-4100 spectrometer. Melting point (mp) was measured by METTLER TOLEDO MP70. Chiral stationary phase HPLC analysis was performed on a Jasco LC-2000 Plus Series system with a DACIEL chiral analytical column (4.6 mm Φ × 250 mm length). Optical rotations were measured on a Jasco P-1020 digital polarimeter with a sodium lamp and reported as follows: [α] T ° C D (c = g/100 mL, solvent, % ee). High resolution mass spectra analysis was performed on a Bruker Daltonics solariX 9.4T FT-ICR-MS spectrometer and a JEOL JMS-T100GCV Time-of-Flight Mass Spectrometer at the Research and Analytical Center for Giant Molecules, Graduate School of Science, Tohoku University. X-ray crystallographic analysis was conducted on a Rigaku XtaLAB Synergy at the same Research and Analytical Center.

2. Preparation of Substrates

Procedure for preparation of **1a**

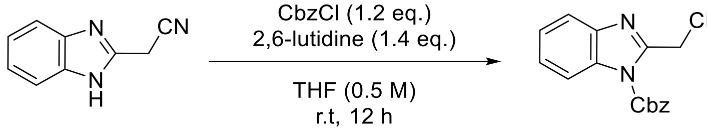

S1 **1a**

To a mixture of **S1** (1.6 g, 10 mmol) and 2,6-lutidine (1.63 mL, 14 mmol) in THF (20 mL) was added CbzCl (1.8 mL, 12 mmol) at 0 °C. After stirring at room temperature for 12 h, the reaction mixture was concentrated under reduced pressure. The residual crude product was recrystallized from CH_2Cl_2/Hexane to obtain **1a** as a yellow solid (2.6 g, 90% yield).

Benzyl 2-(Cyanomethyl)-1*H*-benzo[*d*]imidazole-1-carboxylate (1a)

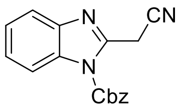

Yellow solid, mp 105.6-109.6 °C; R_f = 0.50 (hexane/EtOAc= 2/1); ^{1}H NMR (600 MHz, $CDCl_3$) δ 7.88-7.91 (m, 1H), 7.75-7.79 (m, 1H), 7.51-7.53 (m, 2H), 7.42-7.47 (m, 3H), 7.35-7.40 (m, 2H), 5.56 (s, 2H), 4.34 (s, 2H); ^{13}C NMR (151 MHz, $CDCl_3$) δ 150.0, 144.7, 141.8, 133.6, 132.6, 129.5, 129.1, 126.0, 125.2, 120.6, 115.1, 114.5, 70.5, 21.9; IR (ATR) 3033, 1748, 1610, 1549, 1497, 1454, 1388, 1338, 1320, 1297, 1261, 1217, 1189, 1121, 1090, 1017, 984, 947, 907, 862, 826, 771, 699, 673 cm^{-1}; HRMS (FD+(eiFi)) m/z: [M] Calcd for $C_{17}H_{13}N_3O_2$, 291.1008; found, 291.1007.

Procedure for preparation of **1b**

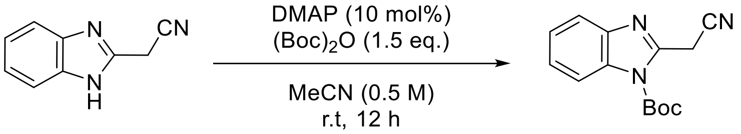

1 **1b**

To a mixture of **1** (785 mg, 5 mmol) and DMAP (61 mg, 0.5 mmol) in MeCN (10 mL) was added $(Boc)_2O$ (1.72 mL, 7.5 mmol). The reaction mixture was stirred at room temperature for 12 h. The mixture was directly passed through flash column chromatography (Hexane/EtOAc = 2/1) to obtain **1b** as a yellow solid (1.2 g, 90% yield).

tert-Butyl 2-(Cyanomethyl)-1*H*-benzo[*d*]imidazole-1-carboxylate (1b)

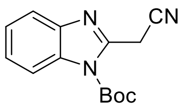

Yellow solid, mp 117.6-1236 °C; R_f = 0.50 (hexane/EtOAc= 2/1); ^{1}H NMR (400 MHz, $CDCl_3$) δ 7.95-7.91 (m, 1H), 7.79-7.75 (m, 1H), 7.42-7.35 (m, 2H), 4.35 (s, 2H), 1.75 (s, 9H); ^{13}C NMR (101 MHz, $CDCl_3$) δ 148.5, 144.8, 141.8, 133.0, 125.7, 124.9, 120.5, 115.1, 114.8, 87.3, 28.1, 22.1; IR (ATR) 2978, 1742, 1626, 1576, 1534, 1511, 1495, 1476, 1450, 1394, 1370, 1351, 1338, 1319, 1297, 1268, 1219, 1151, 1120, 1093, 1034, 1017, 970, 910, 856, 841, 771, 698, 666, 630 cm^{-1}; HRMS (FD+(eiFi)) m/z: [M] Calcd for $C_{14}H_{15}N_3O_2$, 257.1164; found, 257.1164.

Procedure for preparation of **1c**[1]

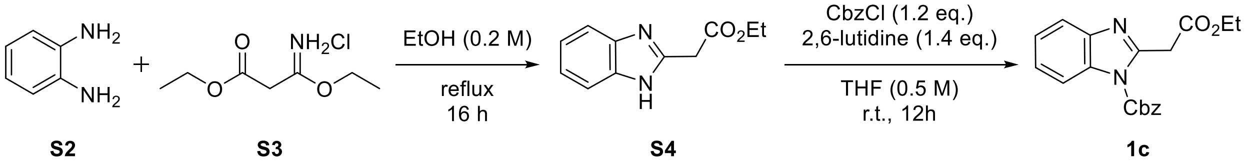

S2 **S3** **S4** **1c**

(1) M. G. Woll, H. Qi, A. Turpoff, N. Zhang, X. Zhang, G. Chen, C. Li, S. Huang, T. Yang, Y.-C. Moon, C.-S. Lee, S. Choi, N. G. Almstead, N. A. Naryshkin, A. Dakka, J. Narasimhan, V. Gabbeta, E. Welch, X. Zhao, N. Risher, J. Sheedy, M. Weetall, G. M. Karp, *J. Med. Chem.* **2016**, *59*, 6070-6085.

A mixture of benzene-1,2-diamine (1.08 g, 10 mmol) and ethyl 3-ethoxy-3-iminopropanoate hydrochloride (1.95 g, 10 mmol) in EtOH (20 mL) was stirred at 80 °C for 16 h. The reaction mixture was partitioned between EtOAc (100 mL) and aqueous saturated $NaHCO_3$ (100 mL). The organic layer was washed with brine and concentrated. The residue was purified by column chromatography (CH_2Cl_2/MeOH = 10/1) to give **S4** (1.59 g 78% yield). To a mixture of a **S4** (1.6 g, 8 mmol), 2,6-lutidine (1.3 mL, 11.2 mmol) in THF (20 mL) was added CbzCl (1.35 mL, 9.6 mmol) at 0 °C. After stirring at room temperature for 12 h, the reaction mixture was concentrated under reduced pressure. The residual crude product was recrystallized from CH_2Cl_2/Hexane to obtain **1a** (2.38 g, 90% yield).

.

Benzyl 2-(2-Ethoxy-2-oxoethyl)-1*H*-benzo[*d*]imidazole-1-carboxylate (1c)

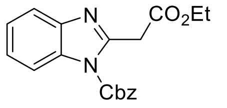

Yellow solid, mp 81.6-83.4 °C; R_f = 0.45 (hexane/EtOAc= 2/1); ^{1}H NMR (600 MHz, $CDCl_3$) δ 7.89-7.92 (m, 1H), 7.70-7.73 (m, 1H), 7.48-7.50 (m, 2H), 7.41-7.45 (m, 3H), 7.31-7.35 (m, 2H), 5.48 (s, 2H), 4.26 (s, 2H), 4.12 (q, *J* = 7.2 Hz, 2H), 1.19 (t, *J* = 7.2 Hz, 3H); ^{13}C NMR (151 MHz, $CDCl_3$) δ 168.5, 150.3, 149.0, 142.1, 134.0, 132.8, 129.2, 128.9, 125.2, 124.6, 120.1, 115.0, 69.8, 61.4, 38.1, 14.1; IR (ATR) 2980, 1740, 1613, 1552, 1455, 1387, 1372, 1336, 1291, 1256, 1199, 1121, 1092, 1028, 985, 907, 879, 855, 761, 747, 698 cm^{-1}; HRMS (FD+) m/z: [M] Calcd for $C_{19}H_{18}N_2O_4$, 338.1267; found, 338.1266.

Procedure for preparation of novel imines **2**[2]

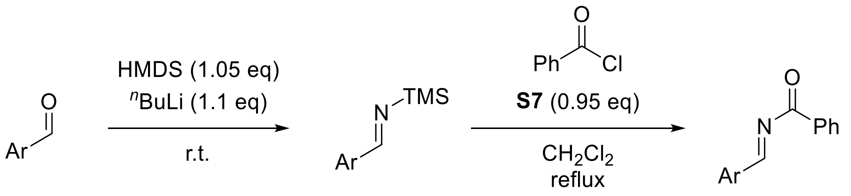

S5 **S6** **2**

In a flame-dried round-bottomed flask equipped with a magnetic stir bar, 1.05 eq. of HMDS (1,1,1,3,3,3-hexamethyldisilazane) was charged. To the reaction mixture, ^{n}BuLi (1.55 M in hexane, 1.1 eq.) was added at 0 °C. After the resulting mixture was stirring at room temperature for 15 minutes, **S5** (1.0 eq.) was added at 0 °C. The reaction mixture was stirred for 1 h at room temperature and solvent was removed *in vacuo*. Then, the resulting mixture was distilled (1 torr, 45 °C-55 °C) to obtain **S6**. To the solution of **S6** in CH_2Cl_2 (0.8 M) was added **S7** (0.95 eq.). After stirring for 3 h at reflux temperature (oil bath at 50 °C), solvent was removed *in vacuo* and the residual mixture was Kugelrohr distilled.

(*E*)-*N*-(2-Fluorobenzylidene)benzamide (2i)

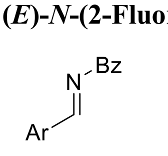

Ar = 2-FC_6H_4

^{1}H NMR (600 MHz, $CDCl_3$) δ 9.09 (s, 1H), 8.20-8.23 (m, 1H), 8.14-8.15 (m, 2H), 7.56-7.62 (m, 2H), 7.47-7.50 (m, 2H), 7.28-7.31 (m, 1H), 7.16-7.19 (m, 1H); ^{19}F NMR (565 MHz, $CDCl_3$) δ -118.6 to -118.7; ^{13}C NMR (151 MHz, $CDCl_3$) δ 181.1, 164.0 (d, *J* = 257 Hz), 158.1 (d, *J* = 5.7 Hz), 135.2 (d, *J* = 8.7 Hz), 133.7, 133.2, 130.3, 128.7 (d, *J* = 9.1 Hz), 124.8 (d, *J* = 2.8 Hz), 122.6 (d, *J* = 8.7 Hz), 116.4 (d, *J* = 27.2 Hz) one carbon was not found due to overlapping; IR (ATR) 3305, 3064, 2977, 2930, 2896, 2879, 1918, 1644, 1602, 1580, 1520, 1486, 1456, 1404, 1346, 1269, 1232, 1185, 1155, 1137, 1106, 1074, 1047, 1029, 1000, 946, 927, 835, 801, 757, 710, 693, 665 cm^{-1}; HRMS (FD+) m/z: $[M]^+$ Calcd for $C_{14}H_{10}FNO$, 227.0746; found, 227.0746.

(2) R. Kupfer, S. Meier, E. Würthwein, *Synthesis* **1984**, *8*, 688-690.

3. Initial Screening of Diastereo- and Enantioselective Mannich-type Reaction of Benzimidazoles with Imines

Representative procedure for the diastereo- and enantioselective Mannich-type reaction of benzimidazoles with imines catalyzed by chiral phosphoric acid

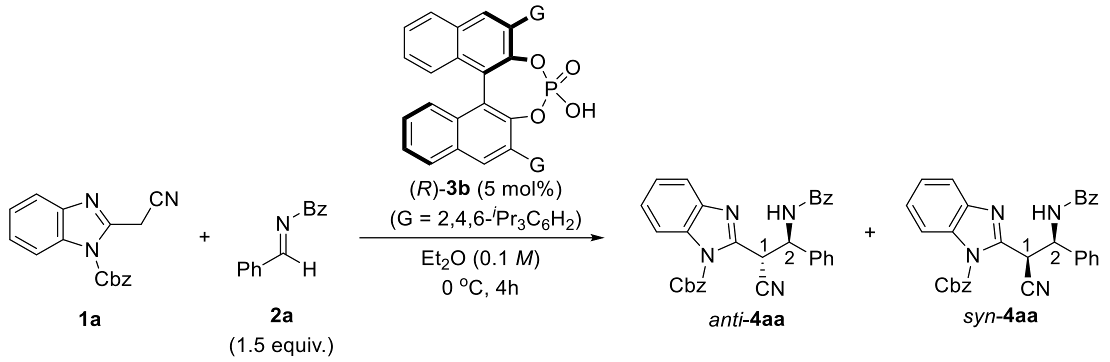

To a mixture of **1a** (58.3 mg, 0.20 mmol), **2a** (62.8 mg, 0.30 mmol) and (*R*)-**3b** (7.3 mg, 0.01 mmol) was added Et_2O (2.0 mL) at 0 °C. The reaction mixture was stirred at 0 °C for 4 h. The mixture was directly passed through flash column chromatography (Hexane/EtOAc = 2/1) to give **4aa** as a colorless oil. The enantiomeric excess was determined by chiral stationary phase HPLC analysis.

Benzyl 2-((1*R*,2*R*)-2-Benzamido-1-cyano-2-phenylethyl)-1*H*-benzo[*d*]imidazole-1-carboxylate [*anti*-(1*R*,2*R*)-4aa]

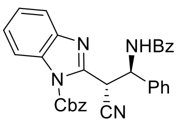

Colorless oil (99% combined yield of diastereomers, 0.2 mmol, 100.1 mg, dr = 95/5); R_f = 0.44 (hexane/EtOAc = 2/1); $[\alpha]^{27.1}_D$ = -19.9 (c 5.65, $CHCl_3$, 98% ee); ^{1}H NMR (600 MHz, $CDCl_3$) δ 8.79 (d, *J* = 8.6 Hz, 1H), 7.95-7.93 (m, 2H), 7.80-7.77 (m, 2H), 7.55 (tt, *J* = 6.6 Hz, 2.4 Hz,1H), 7.51-7.48 (m, 2H), 7.47-7.44 (m, 5H), 7.40-7.34 (m, 2H), 7.19-7.16 (m, 5H), 6.13 (dd, *J* = 8.6, 4.8 Hz, 1H), 5.81 (d, *J* = 4.8 Hz, 1H), 5.45 (d, *J* = 11.7 Hz, 1H), 5.42 (d, *J* = 11.7 Hz, 1H); ^{13}C NMR (151 MHz, $CDCl_3$) δ 166.8, 149.7, 146.5, 141.2, 137.2, 133.9, 133.3, 131.94, 131.87, 129.5, 129.1, 129.0, 128.71, 128.69, 128.3, 126.4, 126.3, 125.3, 120.5, 116.0, 115.3, 70.7, 54.4, 37.3, one carbon was not found due to overlapping; IR (ATR) 3276, 3064, 3032, 1746, 1647, 1602, 1577, 1522, 1487, 1450, 1390, 1320, 1266, 1214, 1144, 1099, 1027, 1002, 989, 932, 909, 845, 789, 746, 695, 614 cm^{-1}; HRMS (FD+) $[M]^+$ Calcd for $C_{31}H_{24}N_4O_3$, 500.1848; found, 500.1846; HPLC analysis CHIRALPAK IC-3 (hexane/^{i}PrOH = 80/20, 1.0 mL/min, 40 °C, 254 nm) 38.4 min (minor), 56.0 min (major); CHIRALPAK IA (hexane/EtOH = 90/10, 0.7 mL/min, 40 °C, 254 nm) 18.9 min (major), 45.9 min (minor). Configuration assignment: The absolute configuration was assigned as (1*R*,2*R*) by analogy with compound **4ac**.

Benzyl 2-((1*S*,2*R*)-2-Benzamido-1-cyano-2-phenylethyl)-1*H*-benzo[*d*]imidazole-1-carboxylate [*syn*-(1*S*,2*R*)-4aa]

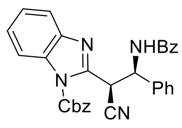

White solid; mp 82.1-88.9 °C; R_f = 0.40 (hexane/EtOAc= 2/1); $[\alpha]^{22.8}_D$ = 23.9 (c 0.50, $CHCl_3$, 94% ee); ^{1}H NMR (600 MHz, $CDCl_3$) δ 7.84-7.82 (m, 1H), 7.72-7.71 (m, 1H), 7.64-7.60 (m, 4H), 7.56 (dd, *J* = 8.1, 1.5 Hz, 2H), 7.49-7.45 (m, 6H), 7.40-7.38 (m, 3H), 7.33-7.31 (m, 2H), 7.02 (d, *J* = 8.9 Hz, 1H), 6.11 (dd, *J* = 9.3, 3.1 Hz, 1H), 5.67 (d, *J* = 11.7 Hz, 1H), 5.63 (d, *J* = 3.1 Hz, 1H), 5.58 (d, *J* = 11.7 Hz, 1H); ^{13}C NMR (151 MHz, $CDCl_3$) δ 167.7, 150.7, 147.2, 141.8, 137.3, 133.9, 133.6, 132.6, 131.9, 129.5, 129.2, 129.2, 129.1, 128.8, 128.7, 127.1, 126.4, 126.2, 125.2, 120.7, 115.9, 115.5, 70.8, 53.3, 42.1; IR (ATR) 3326, 3063, 3033, 1745, 1645, 1603, 1580, 1523, 1487, 1452, 1386, 1348, 1314, 1295, 1261, 1198,1122, 1090, 1028, 1002, 941, 908, 799, 761, 727, 695, 647 cm^{-1}; HRMS (FD+) m/z: $[M+Na]^+$ Calcd for $C_{31}H_{24}N_4O_3$, 523.1741 ; found, 523.1740; HPLC analysis CHIRALPAK IA (hexane/EtOH = 90/10, 0.7 mL/min, 40 °C, 254 nm) 21.8 min (major), 39.2 min (minor). Configuration assignment: The absolute configuration was assigned as (1*S*,2*R*) by analogy with compound **4ac**.

tert-Butyl 2-((1_R_,2_R_)-2-Benzamido-1-cyano-2-phenylethyl)-1_H_-benzo[_d_]imidazole-1-carboxylate (4ba)

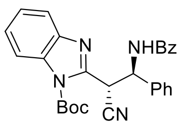

Colorless oil (99% combined yield of diastereomers, 0.2 mmol, 92.9 mg, dr = 93/7); R_f = 0.45 (hexane/EtOAc = 2/1); $[\alpha]^{25.0}_D$ = +8.4 (c 1.23, $CHCl_3$, 97% ee); ^{1}H NMR (600 MHz, $CDCl_3$) δ 9.05 (d, J = 8.6 Hz, 1H), 7.96-7.95 (m, 2H), 7.83-7.78 (m, 2H), 7.54 (t, J = 7.4 Hz, 1H), 7.48 (t, J = 7.4 Hz, 2H), 7.41-7.37 (m, 2H), 7.24-7.16 (m, 5H), 6.14 (dd, J = 8.6, 4.8 Hz, 1H), 5.90 (d, J = 4.8 Hz, 1H), 1.63 (s, 9H); ^{13}C NMR (151 MHz, $CDCl_3$) δ 166.7, 148.3, 146.7, 141.1, 137.5, 133.9, 132.0, 131.8, 128.65, 128.61, 128.2, 127.2, 126.4, 126.0, 125.0, 120.4, 116.2, 115.3, 87.6, 54.6, 36.9, 27.9; IR (ATR) 3324, 3062, 3029, 3008, 2980, 1747, 1645, 1620, 1579, 1525, 1487, 1451, 1382, 1317, 1297, 1264, 1226, 1206, 1150, 1122, 1093, 1028, 931, 835, 750, 699 cm $^{-1}$; HRMS (ESI) m/z: $[M+Na]^+$ Calcd for $C_{28}H_{26}N_4O_3$, 489.1903 found, 489.1897; HPLC analysis CHIRALPAK AD-3 (hexane/^{i}PrOH = 85/15, 1.0 mL/min, 30 °C, 254 nm) 8.9 min (major), 29.3 min minor). Configuration assignment: The absolute configuration was assigned as (1_R_,2_R_) by analogy with compound **4ac**.

Table S1. Optimization of reaction conditions (Table 1)[a]

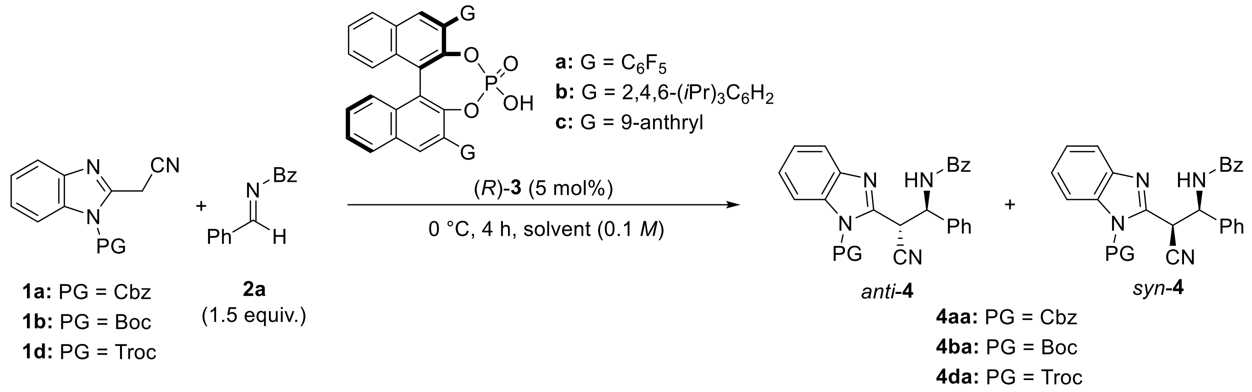

Entry	**1**	CPA	solvent	Yield (%)[b] (_anti_/_syn_)[c]	Ee (%) (_anti_/_syn_)[d]
1	**1a** (R = Cbz)	(_R_)-**3a** (C_6F_5)	Toluene	quant. (60/40)	96/93
2	**1a**	(_R_)-**3a**	CH_2Cl_2	quant. (64/36)	95/60
3	**1a**	(_R_)-**3a**	THF	quant. (85/15)	95/60
4	**1a**	(_R_)-**3a**	Et_2O	quant. (94/6)	97/90
5	**1a**	(_R_)-**3b** (TRIP)	Et_2O	quant. (95/5)	98/43
6	**1a**	(_R_)-**3c** (9-anthryl)	Et_2O	quant. (91/9)	98/38
7	**1b** (R = Boc)	(_R_)-**3a**	Et_2O	quant. (84/16)	95/69
8[e]	**1b**	(_R_)-**3a**	Et_2O	quant. (93/7)	97/80
9	**1b**	(_R_)-**3b**	Et_2O	quant. (83/17)	88/44
10	**1d** (R = Troc)	(_R_)-**3a**	Et_2O	quant. (95/5)	97/-

[a] Reaction conditions: **1** (0.20 mmol), **2a** (0.30 mmol, 1.5 equiv.), (_R_)-**3** (5 mol%, 0.01 mmol) in the indicated solvent (2 mL, 0.1 _M_) under nitrogen atmosphere unless otherwise specified. [b] Combined yield of diastereomeric **4**. [c] Diastereomeric ratio was determined by crude ^{1}H NMR. [d] Enantiomeric excess was determined by HPLC analysis using a chiral stationary phase column. [e] 2.0 equiv. of **2a** was used.

Table S2. Monitor the reaction of 1a with 2a using (*R*)-3a as the catalyst (Scheme 2a up)[a]

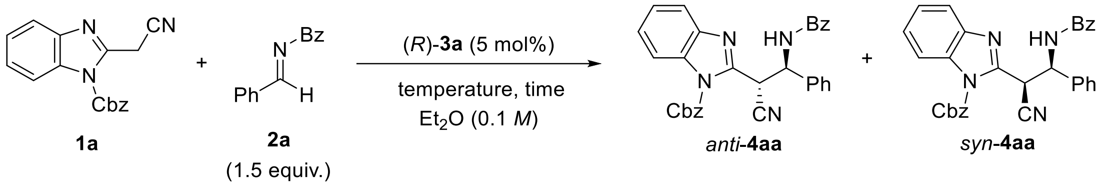

Entry	Time (h)	Temp. (°C)	Conv. (%)[b]	*anti*/*syn*[c]	Ee (*anti*/*syn*)[d]
1	1	0	98%	95/5	96/92
2	2	0	>99%	94/6	96/93
3	4	0	>99%	94/6	96/90
4[e]	24	r.t.	>99%	68/32	92/90
5[e]	48	r.t.	>99%	65/35	92/86

[a] Reaction conditions: **1a** (0.20 mmol), **2a** (0.30 mmol, 1.5 equiv.), (*R*)-**3a** (5 mol%, 0.01 mmol) in Et_2O (2 mL, 0.1 *M*) under nitrogen atmosphere. [b] Conversion of **1a**. [c] Diastereomeric ratio was determined by crude ^1H NMR. [d] Enantiomeric excess was determined by HPLC analysis using a chiral stationary phase column. [e] After the reaction was conducted at 0 °C for 4 hours, the reaction temperature was elevated to room temperature and the reaction was continued for 20 hours (entry 4) and 44 hours (entry 5).

Table S3. Monitor the reaction of 1a with 2a using (*R*)-3b as the catalyst (Scheme 2a down)[a]

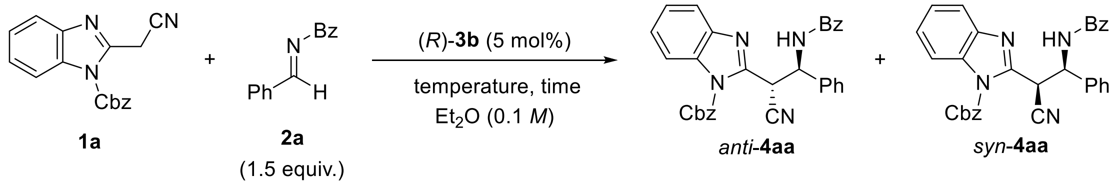

Entry	Time (h)	Temp. (°C)	Conv. (%)[b]	*anti*/*syn*[c]	Ee (*anti*/*syn*)[d]
1	1	0	95%	95/5	98/81
2	2	0	98%	95/5	97/75
3	4	0	>99%	95/5	98/43
4[e]	24	r.t.	>99%	95/5	96/85
5[e]	48	r.t.	>99%	94/6	98/80

[a] Reaction conditions: **1a** (0.20 mmol), **2a** (0.30 mmol, 1.5 equiv.), (*R*)-**3b** (5 mol%, 0.01 mmol) in Et_2O (2 mL, 0.1 *M*) under nitrogen atmosphere. [b] Conversion of **1a**. [c] Diastereomeric ratio was determined by crude ^1H NMR. [d] Enantiomeric excess was determined by HPLC analysis using a chiral stationary phase column. [e] After the reaction was conducted at 0 °C for 4 hours, the reaction temperature was elevated to room temperature and the reaction was continued for 20 hours (entry 4) and 44 hours (entry 5).

Table S4. Monitor the reaction of 1b with 2a using (*R*)-3a as the catalyst[a]

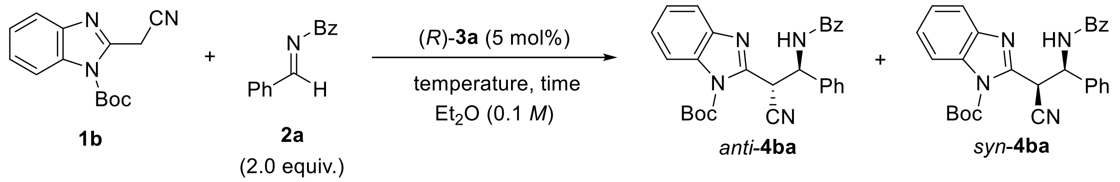

Entry	Time (h)	Temp. (°C)	Conv. (%)[b]	*anti*/*syn*[c]	Ee (*anti*/*syn*)[d]
1	1	0	98%	92/8	97/81
2	2	0	>99%	91/9	97/90
3	3	0	>99%	92/8	97/76
4	4	0	>99%	93/7	97/80
5[e]	9	r.t.	>99%	82/18	94/90
6[f]	24	r.t.	>99%	63/37	93/85
7[f]	48	r.t.	>99%	45/55	93/84

[a] Reaction conditions: **1b** (0.20 mmol), **2a** (0.30 mmol, 1.5 equiv.), (*R*)-**3a** (5 mol%, 0.01 mmol) in Et_2O (2 mL, 0.1 *M*) under nitrogen atmosphere. [b] Conversion of **1b**. [c] Diastereomeric ratio was determined by crude ^{1}H NMR. [d] Enantiomeric excess was determined by HPLC analysis using a chiral stationary phase column. [e] After the reaction was conducted at 0 °C for 4 hours, the reaction temperature was elevated to room temperature and the reaction was continued for 5 hours. [f] The reaction was continued at room temperature for additional 15 hours (entry 6) and 39 hours (entry 7).

4. Scope of Diastereo- and Enantioselective Mannich-type Reaction of Benzimidazoles with Imines

Table S5. Scope of substrates[a]

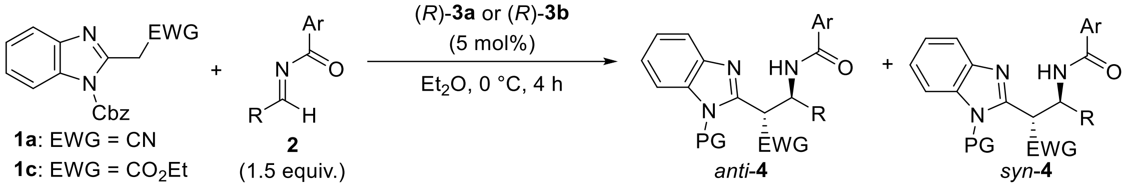

Entry	(*R*)-3	1	2: R, Ar	4	Yield (%) (dr)[b]	Ee (%)[c]	Table 2 entry
1	**3a**	**1a**	**2b**: 4-FC_6H_4, Ph	**4ab**	quant. (>95/5)	98	
	**3b**				quant. (>95:5)	98	entry 1
2	**3a**	**1a**	**2c**: 4-ClC_6H_4, Ph	**4ac**	quant. (85/15)	98	
	**3b**				quant. (>95/5)	98	entry 2
3	**3a**	**1a**	**2d**: 4-BrC_6H_4, Ph	**4ad**	quant. (>95/5)	98	entry4
	**3b**				quant. (>95/5)	98	entry3
4	**3a**	**1a**	**2e**: 4-$CF_3C_6H_4$, Ph	**4ae**	quant. (>95/5)	99	entry 6
	**3b**				quant. (>95/5)	98	
5	**3a**	**1a**	**2f**: 4-MeC_6H_4, Ph	**4af**	quant. (94/6)	99	entry 7
	**3b**				quant. (94/6)	97	
6	**3a**	**1a**	**2g**: 3-MeC_6H_4, Ph	**4ag**	--	--	
	**3b**				quant. (>95/5)	91	entry 8
7	**3a**	**1a**	**2h**:3-FC_6H_4, Ph	**4ah**	quant. (91/9)	92	entry 9
	**3b**				--	--	
8[d]	**3a**	**1a**	**2i**: 2-FC_6H_4, Ph	**4ai**	quant. (>95/5)	99	entry 10
	**3b**				quant. (>95/5)	95	
9[d]	**3a**	**1a**	**2j**: 2-naphthyl, Ph	**4aj**	--	--	
	**3b**				quant. (>95/5)	98	entry 11
10	**3a**	**1a**	**2k**: 2-thiophenyl, Ph	**4ak**	quant. (83/17)	92	entry 12
	**3b**				--	--	
11	**3a**	**1a**	**2l**: Ph, 4-BrC_6H_4	**4al**	--	--	
	**3b**				quant. (94/6)	99	entry 13
12	**3a**	**1a**	**2m**: Ph, 4-MeC_6H_4	**4am**	--	--	
	**3b**				quant. (93/7)	95	entry 14
13	**3a**	**1a**	**2n**: cyclohexyl, Ph	**4an**	63% (87/13)	29/18	entry 15
	**3b**				58% (85/15)	28/22	
14[e]	**3a**	**1c**	**2a**: Ph, Ph	**4ca**	quant. (73/27)	90/81	entry 16
	**3b**				quant. (80/20)	85/80	

[a] Reaction conditions: **1a** (0.20 mmol), **2** (0.30 mmol), (*R*)-**3** (5 mol%, 0.01 mmol) in Et_2O (2 mL, 0.1 *M*) under nitrogen atmosphere at 0 °C for 4 h unless otherwise specified. [b] Diastereomeric mixtures were formed quantitatively and diastereomeric ratio (*anti*/*syn*) was determined by crude ^{1}H NMR. [c] Enantiomeric excess was determined by HPLC analysis using a chiral stationary phase column. [d] For 12 h. [e] Use **1c** (0.20 mmol), instead of **1a**, for 16 h.

Benzyl 2-((1*R*,2*R*)-2-Benzamido-1-cyano-2-(4-fluorophenyl)ethyl)-1*H*-benzo[*d*]imidazole-1-carboxylate (4ab)

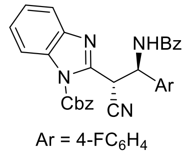

Colorless oil (99% combined yield of diastereomers, 0.2 mmol, 103.2 mg, dr = >95/5); R_f = 0.40 (hexane/EtOAc= 2/1); $[\alpha]^{22.0}_{D}$ = -39.1 (c 2.26, $CHCl_3$, 98% ee); ^{1}H NMR (600 MHz, $CDCl_3$) δ 8.83 (d, *J* = 8.4 Hz, 1H), 7.94-7.92 (m, 2H), 7.79-7.77 (m, 2H), 7.55 (tt, *J* = 7.8 Hz, 1.2 Hz, 1H), 7.49-7.44 (m, 7H), 7.39 (dt, *J* = 7.2 Hz, 1.2 Hz, 1H), 7.35 (dt, *J* = 8.4 Hz, 1.8 Hz, 1H), 7.18-7.15 (m, 2H), 6.85 (tt, *J* = 8.4 Hz, 1.8 Hz, 2H), 6.10 (dd, *J* = 8.4, 4.8 Hz, 1H), 5.80 (d, *J* = 4.8 Hz, 1H), 5.47 (d, *J* = 11.4 Hz, 1H), 5.44 (d, *J* = 11.4 Hz, 1H); ^{13}C NMR (151 MHz, $CDCl_3$) δ 166.8, 162.4 (d, *J* = 247.2 Hz), 149.8, 146.4, 141.1, 133.7, 133.3, 133.1 (d, *J* = 2.8 Hz), 132.0, 131.9, 129.5, 129.1, 129.0, 128.7, 128.3 (d, *J* = 7.2 Hz), 127.2, 126.4, 125.4, 120.5, 115.8, 115.7, 115.7 (d, *J* = 21.7 Hz), 70.7, 53.9, 37.2; ^{19}F NMR (565 MHz, $CDCl_3$) δ -113.1 (m, 1F); IR (ATR) 3323, 3033, 2964, 1751, 1651, 1530, 1509, 1486, 1453, 1388, 1346, 1313, 1296, 1262, 1219, 1124, 1092, 835, 772, 698 cm^{-1}; HRMS (FD+) m/z: $[M]^+$ Calcd for $C_{31}H_{23}FN_4O_3$, 518.1754; found, 518.1753; HPLC analysis CHIRALPAK IA (hexane/EtOH = 80/20, 1.0 mL/min, 40 °C, 254 nm) 8.8 min (major), 26.5 min (minor). Configuration assignment: The absolute configuration was assigned as (1*R*,2*R*) by analogy with compound **4ac**.

Benzyl 2-((1*R*,2*R*)-2-Benzamido-2-(4-chlorophenyl)-1-cyanoethyl)-1*H*-benzo[*d*]imidazole-1-carboxylate [*anti*-(1*R*,2*R*)-4ac]

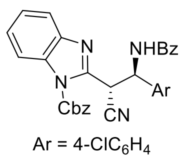

White solid (99% combined yield of diastereomers, 0.2 mmol, 107.0 mg, dr = >95/5); mp 159.8-164.8 °C; R_f = 0.40 (hexane/EtOAc= 2/1); $[\alpha]^{22.8}_{D}$ = -15.6 (c 1.31, $CHCl_3$, 98% ee); ^{1}H NMR (600 MHz, $CDCl_3$) δ 8.89 (d, J = 8.4 Hz, 1H), 7.95-7.93 (m, 2H), 7.78 (m, 2H), 7.57-7.55 (m, 1H), 7.51-7.45 (m, 7H), 7.39 (dt, *J* = 7.2 Hz, 1.2 Hz, 1H), 7.36 (dt, *J* = 8.4 Hz, 1.8 Hz, 1H), 7.16-7.12 (m, 4H), 6.11 (dd, J = 8.4, 4.8 Hz, 1H), 5.80 (d, *J* = 4.8 Hz, 1H), 5.47 (d, *J* = 11.4 Hz, 1H), 5.44 (d, *J* = 11.4 Hz, 1H); ^{13}C NMR (151 MHz, $CDCl_3$) δ 166.8, 149.8, 146.2, 141.1, 135.8, 134.2, 133.6, 133.2, 132.0, 131.8, 129.6, 129.12, 129.09, 128.9, 128.7, 127.9, 127.3, 126.5, 125.4, 120.5, 115.7, 115.4, 70.8, 53.9, 37.0; IR (ATR) 3446, 3005, 2989, 1739, 1699, 1647, 1598, 1576, 1541, 1490, 1451, 1384, 1315, 1297, 1275, 1260, 1203, 1141, 1123, 1091, 1026, 1014, 970, 934, 896, 820, 750, 694, 628 cm^{-1}; HRMS (FD+) m/z: $[M]^+$ Calcd for $C_{31}H_{23}ClN_4O_3$, 534.1459; found, 534.1457; HPLC analysis CHIRALPAK IA (hexane/EtOH = 80/20, 1.0 mL/min, 40 °C, 254 nm) 9.1 min (major), 28.2 min (minor). Configuration assignment: The absolute configuration was assigned as (1*R*,2*R*) by single-crystal X-ray analysis of *syn*-(1*S*,2*R*)-**4ac** after epimerization at the C1 position of *anti*-(1*R*,2*R*)-**4ac** (see **section 6** for stereochemical determination).

Benzyl 2-((1*S*,2*R*)-2-benzamido-2-(4-chlorophenyl)-1-cyanoethyl)-1*H*-benzo[*d*]imidazole-1-carboxylate [*syn*-(1*S*,2*R*)-4ac]

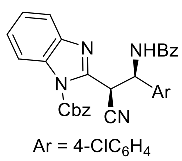

White solid; mp 74.4-76.8 °C; R_f = 0.40 (hexane/EtOAc= 2/1); $[\alpha]^{22.8}_{D}$ = 45.7 (c 0.55, $CHCl_3$, 99% ee); ^{1}H NMR (600 MHz, $CDCl_3$) δ 7.83-7.82 (m, 1H), 7.73-7.72 (m, 1H), 7.64 (dd, J = 8.4, 1.2 Hz, 2H), 7.56-7.53 (m, 4H), 7.51-7.47 (m, 4H), 7.43-7.39 (m, 4H), 7.34-7.33 (m, 2H), 7.04 (d, J = 8.6 Hz, 1H), 6.02 (dd, J = 8.8, 3.3 Hz, 1H), 5.66 (d, J = 11.7 Hz, 1H), 5.59-5.57 (m, 2H); ^{13}C NMR (151 MHz, $CDCl_3$) δ 167.7, 150.8, 146.9, 141.8, 136.1, 134.8, 133.7, 133.6, 132.5, 132.2, 129.7, 129.5, 129.3, 129.2, 128.8, 127.9, 127.2, 126.4, 125.4, 120.8, 115.6, 71.0, 53.1, 41.8, one carbon was not found due to overlapping; IR (ATR) 3262, 1758, 1636, 1579, 1518, 1490, 1451, 1376, 1318, 1257, 1200, 1119, 1081, 1013, 893, 764, 693 cm^{-1}; HRMS (FD+) m/z: $[M+Na]^+$ Calcd for $C_{31}H_{23}ClN_4O_3$, 557.1351; found, 557.1350; HPLC analysis CHIRALPAK IA (hexane/EtOH = 80/20, 1.0 mL/min, 40 °C, 254 nm) 11.8 min (major), 20.0 min (minor). Configuration assignment: The absolute configuration was assigned as (1*S*,2*R*)-**4ac** by single-crystal X-ray analysis (see **section 6** for stereochemical determination).

Benzyl 2-((1*R*,2*R*)-2-Benzamido-2-(4-bromophenyl)-1-cyanoethyl)-1*H*-benzo[*d*]imidazole-1-carboxylate (4ad)

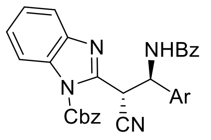

Ar = 4-BrC_6H_4

White solid (99% combined yield of diastereomers, 0.2 mmol, 115.5 mg, dr = >95/5); mp 158.4-162.4 °C; R_f = 0.43 (hexane/EtOAc = 2/1); $[\alpha]^{25.3}_D$ = -44.6 (c 0.82, $CHCl_3$, 98% ee); ^{1}H NMR (600 MHz, $CDCl_3$) δ 8.93 (d, *J* = 8.6 Hz, 1H), 7.96-7.94 (m, 2H), 7.80-7.76 (m, 2H), 7.58 (tt, *J* = 7.2 Hz, 1.8 Hz, 1H), 7.51 (t, *J* = 8.4 Hz, 2H), 7.49-7.46 (m, .5H), 7.40 (dt, *J* = 7.2 Hz, 1.2 Hz, 1H), 7.37 (dt, *J* = 7.8 Hz, 1.2 Hz, 1H), 7.31 (dt, *J* = 8.4 Hz, 1.8 Hz, 2H), 7.08-7.06 (m, 2H), 6.09 (dd, *J* = 8.6 Hz, 4.5 Hz, 1H), 5.80 (d, *J* = 4.5 Hz, 1H), 5.48 (d, *J* = 11.7 Hz, 1H), 5.44 (d, *J* = 11.7 Hz, 1H); ^{13}C NMR (151 MHz, $CDCl_3$) δ 166.8, 149.8, 146.2, 141.0, 136.4, 133.6, 133.2, 132.0, 131.84, 131.77, 129.6, 129.1, 128.7, 128.2, 127.3, 126.5, 125.5, 122.4, 120.5, 115.7, 115.4, 70.8, 53.9, 36.8, one carbon was not found due to overlapping; IR (ATR) 3325, 3064, 3031, 1749, 1658, 1646, 1602, 1579, 1540, 1523, 1487, 1453, 1387, 1346, 1314, 1296, 1262, 1199, 1124, 1093, 1077, 1011, 894, 828, 801, 758, 697 cm^{-1}; HRMS (FD+) m/z: $[M]^+$ Calcd for $C_{31}H_{23}BrN_4O_3$, 578.0954; found, 578.0952; HPLC analysis CHIRALPAK IC-3 (hexane/EtOH = 95/5, 1.0 mL/min, 40 °C, 254 nm) 20.6 min (minor), 22.4 min (major). Configuration assignment: The absolute configuration was assigned as (1*R*,2*R*) by analogy with compound **4ac**.

Benzyl 2-((1*R*,2*R*)-2-Benzamido-1-cyano-2-(4-(trifluoromethyl)phenyl)ethyl)-1*H*-benzo[*d*]imidazole-1-carboxylate (4ae)

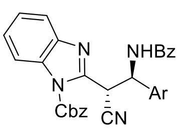

Ar = 4-$CF_3C_6H_4$

White solid (99% combined yield of diastereomers, 0.2 mmol, 113.7 mg, dr = >95/5); mp 152.4-156.4 °C; R_f = 0.43 (hexane/EtOAc= 2/1); $[\alpha]^{22.0}_D$ = -39.1 (c 2.26, $CHCl_3$, 99% ee); ^{1}H NMR (600 MHz, $CDCl_3$) δ 9.04 (d, *J* = 8.2 Hz, 1H), 7.97-7.95 (m, 2H), 7.79 (d, *J* = 7.2 Hz, 1H), 7.77 (d, *J* = 8.4 Hz, 1H), 7.58 (t, *J*= 7.2 Hz, 1H), 7.51 (t, *J* = 7.8 Hz, 2H), 7.48-7.45 (m, 7H), 7.42-7.37 (m, 2H), 7.35-7.34 (m, 2H), 6.19 (dd, *J* = 8.2 Hz, 4.5 Hz, 1H), 5.83 (d, *J* = 4.5 Hz, 1H), 5.48 (d, *J* = 11.7 Hz, 1H), 5.41 (d, *J* = 11.7 Hz, 1H); ^{13}C NMR (151 MHz, $CDCl_3$) δ 166.9, 149.8, 146.1, 141.3, 141.0, 133.5, 133.2, 132.1, 131.8, 130.5 (q, *J* = 33.2 Hz), 129.6, 129.13, 129.11, 128.8, 127.3, 127.0, 126.6, 125.7 (q, *J* = 2.9 Hz), 125.5, 123.7 (q, *J* = 265.9 Hz), 120.5, 115.6, 115.4, 70.9, 54.1, 36.8; ^{19}F NMR (565 MHz, $CDCl_3$) δ -62.6 (s, 3F); IR (ATR) 3323, 3033, 2964, 1751, 1651, 1530, 1509, 1486, 1453, 1388, 1346, 1313, 1296, 1262, 1219, 1124, 1092, 835, 772, 698 cm^{-1}; HRMS (FD+) m/z: $[M]^+$ Calcd for $C_{32}H_{23}F_3N_4O_3$, 568.1722; found, 568.1721; HPLC analysis CHIRALPAK AD-3 (hexane/^{i}PrOH = 80/20, 1.0 mL/min, 30 °C, 254 nm) 9.6 min (major), 30.9 min (minor). Configuration assignment: The absolute configuration was assigned as (1*R*,2*R*) by analogy with compound **4ac**.

Benzyl 2-((1*R*,2*R*)-2-Benzamido-2-(4-methylphenyl)-1-cyanoethyl)-1*H*-benzo[*d*]imidazole-1-carboxylate (4af)

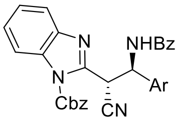

Ar = 4-MeC_6H_4

Colorless oil (99% combined yield of diastereomers, 0.2 mmol, 103.1 mg, dr = 94/6); R_f = 0.42 (hexane/EtOAc= 2/1); $[\alpha]^{24.0}_D$ = -53.9 (c 0.44, $CHCl_3$, 99% ee); ^{1}H NMR (600 MHz, $CDCl_3$) δ 8.71 (d, *J* = 8.4 Hz, 1H), 7.91 (d, *J* = 7.2 Hz, 2H), 7.78 (d, *J* = 8.9 Hz, 2H), 7.53 (dt, *J* = 7.2 Hz, 1.8 Hz, 1H), 7.49-7.43 (m, 7H), 7.39-7.32 (m, 2H), 7.08 (d, *J* = 8.2 Hz, 2H), 6.99 (d, *J* = 8.2 Hz, 2H), 6.10 (dd, *J* = 8.4, 4.8 Hz, 1H), 5.78 (d, *J* = 4.8 Hz, 1H), 5.46 (d, *J* = 11.4 Hz, 1H), 5.43 (d, *J* = 11.4 Hz, 1H), 2.21 (s, 3H); ^{13}C NMR (151 MHz, $CDCl_3$) δ 166.8, 149.7, 146.6, 141.2, 138.0, 134.2, 133.9, 133.4, 132.0, 131.8, 129.5, 129.4, 129.05, 129.02, 128.6, 127.2, 126.4, 126.2, 125.2, 120.5, 116.0, 115.3, 70.6, 54.2, 37.5, 21.0; IR (ATR) 3337, 2959, 2925, 1751, 1666, 1609, 1580, 1509, 1486, 1454, 1386, 1346, 1313, 1297, 1249, 1200, 1177, 1146, 1124, 1105, 1039, 973, 912, 861, 831, 748, 698 cm^{-1}; HRMS (FD+) m/z: $[M]^+$ Calcd for $C_{32}H_{26}N_4O_3$, 514.2005; found, 514.2003; HPLC analysis CHIRALPAK IA (hexane/EtOH = 80/20, 1.0 mL/min, 40 °C, 254 nm) 9.4 min (major), 23.4 min (minor). Configuration assignment: The absolute configuration was assigned as (1*R*,2*R*) by analogy with compound **4ac**.

Benzyl 2-((1*R*,2*R*)-2-Benzamido-1-cyano-2-(*m*-tolyl)ethyl)-1*H*-benzo[*d*]imidazole-1-carboxylate (4ag)

Colorless oil (99% combined yield of diastereomers, 0.2 mmol, 102.6 mg, dr = >95/5); R_f = 0.42 (hexane/EtOAc= 2/1); $[\alpha]^{23.4}_D$ = -57.6 (c 1.25, $CHCl_3$, 91% ee); ^{1}H NMR (600 MHz, $CDCl_3$) δ 8.71 (br, 1H), 7.93 (d, *J* = 7.2 Hz, 2H), 7.78 (t, *J* = 8.4 Hz, 2H), 7.54 (t, *J* = 7.2 Hz, 1H), 7.49-7.44 (m, 7H), 7.40-7.33 (m, 2H), 7.07 (t, *J* = 7.8 Hz, 1H), 6.98-6.96 (m, 3H),

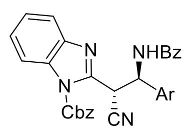

Ar = 3-MeC$_6$H$_4$

6.09 (dd, J = 8.4, 4.8 Hz, 1H), 5.79 (d, J = 4.8 Hz, 1H), 5.44 (d, J = 11.4 Hz, 1H), 5.41 (d, J = 11.4 Hz, 1H), 2.17 (s, 3H); ^{13}C NMR (151 MHz, CDCl$_3$) δ 166.7, 149.7, 146.6, 141.2, 138.4, 137.1, 133.9, 133.4, 132.0, 131.8, 129.5, 129.1, 129.04, 128.98, 128.65, 128.58, 127.24, 127.2, 126.3, 125.3, 123.4, 120.5, 116.0, 115.3, 70.6, 54.5, 37.4, 21.3; IR (ATR) 3330, 3063, 3032, 1749, 1646, 1604, 1579, 1521, 1486, 1452, 1386, 1345, 1312, 1295, 1262, 1197, 1123, 1091, 907, 789, 761, 728, 696, 647 cm^{-1}; HRMS (FD+) m/z: [M]$^+$ Calcd for $C_{32}H_{26}N_4O_3$, 514.2005; found, 514.2002; HPLC analysis CHIRALPAK IC-3 (hexane/^{i}PrOH = 80/20, 1.0 mL/min, 40 °C, 254 nm) 47.9 min (minor), 60.9 min (major). Configuration assignment: The absolute configuration was assigned as (1R,2R) by analogy with compound **4ac**.

Benzyl 2-((1*R*,2*R*)-2-Benzamido-1-cyano-2-(3-fluorophenyl)ethyl)-1*H*-benzo[*d*]imidazole-1-carboxylate (4ah)

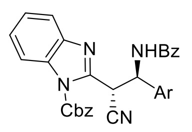

Ar = 3-FC$_6$H$_4$

Colorless oil (99% combined yield of diastereomers, 0.2 mmol, 103.7 mg, dr = 91/9); R$_f$ = 0.43 (hexane/EtOAc= 2/1); $[\alpha]^{23.0}_D$ = -40.1 (c 1.60, CHCl$_3$, 92% ee);^{1}H NMR (400 MHz, CDCl$_3$) δ 8.95 (d, J = 8.4 Hz, 1H), 7.95 (d, J = 6.2 Hz, 2H), 7.81-7.77 (m, 2H), 7.56 (tt, J = 7.6 Hz, 1.6 Hz, 1H), 7.52-7.43 (m, 7H), 7.42-7.33 (m, 2H), 7.17-7.11 (m, 1H), 6.99-6.92 (m, 2H), 6.85 (dt, J = 8.0 Hz, 1.6 Hz, 1H), 6.14 (dd, J = 8.4, 4.8 Hz, 1H), 5.79 (d, J = 4.8 Hz, 1H), 5.48 (d, J = 11.6, 1H), 5.45 (d, J = 11.6 Hz, 1H); ^{13}C NMR (100 MHz, CDCl$_3$) δ 166.9, 162.7 (d, J = 246 Hz), 149.7, 146.2, 141.1, 139.9 (d, J = 6.7 Hz), 133.6, 133.2, 132.0, 131.8, 130.4 (d, J = 8.6 Hz), 129.5, 129.09, 129.05, 128.7, 127.2, 126.5, 125.4, 122.1 (d, J = 2.9 Hz), 120.5, 115.6 (d, J = 28.5 Hz), 115.3, 115.2, 113.8 (d, J = 22.9 Hz), 70.8, 53.9, 37.0; ^{19}F NMR (377 MHz, CDCl$_3$) δ -111.5 to -111.6 (m, 1F); IR (ATR) 3320, 3063, 3034, 1751, 1651, 1615, 1593, 1581, 1527, 1486, 1453, 1387, 1346, 1313, 1295, 1263, 1218, 1201, 1124, 1093, 1027, 956, 903, 772, 696, 631 cm^{-1}; HRMS (FD+) m/z: [M]$^+$ Calcd for $C_{31}H_{23}FN_4O_3$, 518.1754; found, 518.1754; HPLC analysis CHIRALPAK AD-3 (hexane/EtOH = 80/20, 1.0 mL/min, 40 °C, 254 nm) 11.2 min (major), 25.1 min (minor). Configuration assignment: The absolute configuration was assigned as (1R,2R) by analogy with compound **4ac**.

Benzyl 2-((1*R*,2*R*)-2-Benzamido-1-cyano-2-(2-fluorophenyl)ethyl)-1*H*-benzo[*d*]imidazole-1-carboxylate (4ai)

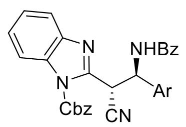

Ar = 2-FC$_6$H$_4$

Colorless oil (99% combined yield of diastereomers, 0.2 mmol, 103.5 mg, dr = >95/5); R$_f$ = 0.38 (hexane/EtOAc= 2/1); $[\alpha]^{23.4}_D$ = -31.9 (c 3.04, CHCl$_3$, 99% ee); ^{1}H NMR (400 MHz, CDCl$_3$) δ 8.74 (d, J = 9.2 Hz, 1H), 7.94-7.90 (m, 2H), 7.81-7.77 (m, 2H), 7.56 (tt, J = 7.2 Hz, 1.2 Hz, 1H), 7.52-7.42 (m, 7H), 7.41-7.33 (m, 2H), 7.22-7.11 (m, 2H), 7.02-6.89 (m, 2H), 6.38 (dd, J = 9.2, 5.2 Hz, 1H), 5.86 (d, J = 5.2 Hz, 1H), 5.48 (d, J = 12.0 Hz, 1H), 5.45 (d, J = 12.0 Hz, 1H); ^{13}C NMR (100 MHz, CDCl$_3$) δ 166.7, 162.7 (d, J = 256 Hz), 149.7, 146.4, 141.1, 133.6, 133.5, 132.0 (d, J = 9.0 Hz), 130.2 (d, J = 7.6 Hz), 129.5, 129.1, 129.0, 128.7, 128.42, 128.39, 127.2, 126.4, 125.3, 124.4 (d, J = 3.8 Hz), 124.2 (d, J = 13.3 Hz), 120.5, 115.7, 115.6 (d, J = 20.4 Hz), 115.3, 70.7, 49.7, 36.2; ^{19}F NMR (377 MHz, CDCl$_3$) δ -117.2--117.2 (m, 1F); IR (ATR) 3342, 3065, 3030, 1752, 1667, 1617, 1602, 1580, 1520, 1487, 1453, 1388, 1347, 1313, 1296, 1264, 1198, 1156, 1124, 1092, 1027, 1000, 944, 904, 840, 802, 754, 712, 696 cm^{-1}; HRMS (FD+) m/z: [M]$^+$ Calcd for $C_{31}H_{23}FN_4O_3$, 518.1754; found, 518.1752; HPLC analysis CHIRALPAK AD-3 (hexane/^{i}PrOH = 80/20, 1.0 mL/min, 40 °C, 254 nm) 11.2 min (minor), 26.9 min (major). Configuration assignment: The absolute configuration was assigned as (1R,2R) by analogy with compound **4ac**.

Benzyl 2-((1*R*,2*R*)-2-Benzamido-1-cyano-2-(naphthalen-2-yl)ethyl)-1*H*-benzo[*d*]imidazole-1-carboxylate (4aj)

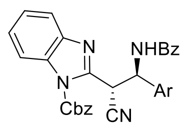

Ar = 2-naphthyl

Colorless oil (99% combined yield of diastereomers, 0.2 mmol, 110.2 mg, dr = >95/5); R$_f$ = 0.41 (hexane/EtOAc= 2/1); $[\alpha]^{24.8}_D$ = -17.1 (c 0.67, CHCl$_3$, 98% ee); ^{1}H NMR (600 MHz, CDCl$_3$) δ 8.96 (d, J = 8.4 Hz, 1H), 7.99-7.98 (m, 2H), 7.81-7.79 (m, 1H), 7.74-7.72 (m, 1H), 7.70-7.65 (m, 4H), 7.57 (tt, J = 7.2 Hz, 1.2 Hz, 1H), 7.51 (t, J = 7.8 Hz, 2H), 7.43-7.35 (m, 8H), 7.33-7.30 (m, 1H), 7.29-7.27 (m, 1H), 6.30 (dd, J = 8.4, 4.8 Hz, 1H), 5.94 (d, J = 4.8 Hz, 1H), 5.34 (d, J = 12.0 Hz, 1H), 5.21 (d, J = 12.0 Hz, 1H); ^{13}C NMR (151 MHz, CDCl$_3$) δ 166.9, 149.7, 146.5, 141.2, 134.7, 133.9, 133.2, 133.0, 131.92, 131.90, 129.4, 129.02, 128.97, 128.72, 128.67, 128.0, 127.5, 127.3, 126.43, 126.36, 126.0, 125.3, 123.9, 120.5, 116.0, 115.3, 70.6, 54.7, 37.2, two carbons were

not found due to overlapping; IR (ATR) 3337, 3060, 1750, 1662, 1524, 1486, 1453, 1387, 1315, 1296, 1263, 1201, 1124, 1093, 751, 698 cm^{-1}; HRMS (FD+) m/z: [M]$^+$ Calcd for $C_{35}H_{26}N_4O_3$, 550.2005; found, 550.2001; HPLC analysis CHIRALPAK IC-3 (hexane/EtOH = 80/20, 1.0 mL/min, 40 °C, 254 nm) 38.1 min (minor), 44.4 min (major). Configuration assignment: The absolute configuration was assigned as (1*R*,2*R*) by analogy with compound **4ac**.

Benzyl 2-((1*R*,2*R*)-2-Benzamido-1-cyano-2-(thiophen-2-yl)ethyl)-1*H*-benzo[*d*]imidazole-1-carboxylate (4ak)

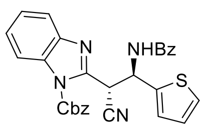

Colorless oil (99% combined yield of diastereomers, 0.2 mmol, 101.2 mg, dr = 83/17); R_f = 0.41 (hexane/EtOAc= 2/1); $[\alpha]^{21.9}_D$ = -14.0 (c 0.82, $CHCl_3$, 92% ee); ^{1}H NMR (600 MHz, $CDCl_3$) δ 8.92 (d, *J* = 9.0 Hz, 1H), 7.94-7.92 (m, 2H), 7.86-7.84 (m, 1H), 7.80-7.78 (m, 1H), 7.55 (tt, *J* = 7.2 Hz, 1.2 Hz, 1H), 7.52-7.44 (m, 7H), 7.42-7.36 (m, 2H), 7.07 (dd, *J* = 5.0, 1.2 Hz, 1H), 6.98 (dt, *J* = 3.6, 1.3 Hz, 1H), 6.82 (dd, *J* = 5.0, 3.6 Hz, 1H), 6.45 (dd, *J* = 9.0, 4.5 Hz, 1H), 5.77 (d, *J* = 4.5 Hz, 1H), 5.55 (d, *J* = 11.4 Hz, 1H), 5.53 (d, *J* = 11.4 Hz, 1H); ^{13}C NMR (151 MHz, $CDCl_3$) δ 166.7, 149.8, 146.4, 141.2, 140.7, 133.7, 133.4, 132.1, 132.0, 129.5, 129.2, 129.1, 128.7, 127.3, 127.0, 126.4, 126.0, 125.4, 125.4, 120.6, 115.5, 115.4, 70.8, 50.5, 37.8; IR (ATR) 3350, 3061, 2963, 1752, 1666, 1653, 1514, 1484, 1453, 1386, 1345, 1316, 1294, 1263, 1199, 1123, 1093, 749, 699 cm^{-1}; HRMS (FD+) m/z: [M]$^+$ Calcd for $C_{29}H_{22}N_4O_3S$, 506.1413; found, 506.1411; HPLC analysis CHIRALPAK IC-3 (hexane/EtOH = 90/10, 1.0 mL/min, 40 °C, 254 nm) 29.8 min (minor), 41.6 min (major). Configuration assignment: The absolute configuration was assigned as (1*R*,2*R*) by analogy with compound **4ac**.

Benzyl 2-((1*R*,2*R*)-2-(4-Bromobenzamido)-1-cyano-2-phenylethyl)-1*H*-benzo[*d*]imidazole-1-carboxylate (4al)

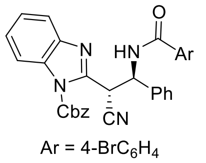

Colorless oil (99% combined yield of diastereomers, 0.2 mmol, 115.5 mg, dr = 94/6); R_f = 0.39 (hexane/EtOAc= 2/1); $[\alpha]^{24.5}_D$ = -35.8 (c 5.10, $CHCl_3$, 99% ee); ^{1}H NMR (600 MHz, $CDCl_3$) δ 8.87 (d, J = 8.6 Hz, 1H), 7.81-7.76 (m, 4H), 7.62 (dt, *J* = 9.0 Hz, 1.8 Hz, 2H), 7.46-7.44 (m, 5H), 7.41-7.34 (m, 2H), 7.18-7.15 (m, 5H), 6.11 (dd, *J* = 8.6, 4.8 Hz, 1H), 5.80 (d, *J* = 4.8 Hz, 1H), 5.44 (d, *J* = 12.0 Hz, 1H), 5.41 (d, *J* = 12.0 Hz, 1H); ^{13}C NMR (151 MHz, $CDCl_3$) δ 165.9, 149.7, 146.5, 141.1, 137.0, 133.3, 132.7, 131.92, 131.87, 129.5, 129.07, 129.03, 128.8, 128.7, 128.4, 126.6, 126.42, 126.38, 125.4, 120.5, 115.9, 115.3, 70.7, 54.5, 37.2; IR (ATR) 3319, 3062, 3034, 2962, 1750, 1659, 1590, 1525, 1480, 1454, 1387, 1347, 1313, 1296, 1262, 1199, 1123, 1091, 1071, 1010, 844, 751, 699 cm^{-1}; HRMS (FD+) m/z: [M]$^+$ Calcd for $C_{31}H_{23}BrN_4O_3$, 578.0954; found, 578.0951; HPLC analysis CHIRALPAK IA (hexane/EtOH = 80/20, 1.0 mL/min, 40 °C, 254 nm) 11.4 min (major), 22.6 min (minor). Configuration assignment: The absolute configuration was assigned as (1*R*,2*R*) by analogy with compound **4ac**.

Benzyl 2-((1*R*,2*R*)-1-Cyano-2-(4-methylbenzamido)-2-phenylethyl)-1*H*-benzo[*d*]imidazole-1-carboxylate (4am)

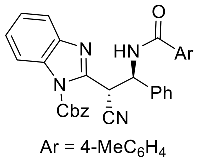

Colorless oil (99% combined yield of diastereomers, 0.2 mmol, 103.0 mg, dr = 93/7); R_f = 0.41 (hexane/EtOAc= 2/1); $[\alpha]^{25.8}_D$ = -48.6 (c 4.11, $CHCl_3$, 95% ee); ^{1}H NMR (600 MHz, $CDCl_3$) δ 8.70 (d, *J* = 8.6 Hz, 1H), 7.83 (d, *J* = 8.4 Hz, 2H), 7.78 (t, *J* = 7.8 Hz, 2H), 7.47-7.44 (m, 5H), 7.38 (dt, *J* = 7.2 Hz, 1.2 Hz, 1H). 7.35 (dt, *J* = 7.8 Hz, 1.2 Hz, 1H), 7.29 (d, *J* = 7.8 Hz, 2H), 7.18-7.16 (m, 5H), 6.12 (dd, *J* = 8.6, 4.8 Hz, 1H), 5.81 (d, *J* = 4.8 Hz, 1H), 5.45 (d, *J* = 11.7 Hz, 1H), 5.42 (d, *J* = 11.7 Hz, 1H), 2.42 (s, 3H); ^{13}C NMR (151 MHz, $CDCl_3$) δ 166.8, 149.7, 146.5, 142.4, 141.2, 137.3, 133.4, 132.0, 131.0, 129.5, 129.3, 129.1, 128.7, 128.3, 127.3, 126.5, 126.3, 125.3, 120.5, 116.0, 115.3, 70.6, 54.4, 37.4, 21.5, one carbon was not found due to overlapping; IR (ATR) 3446, 3005, 2989, 1698, 1632, 1613, 1570, 1541, 1497, 1475, 1453, 1411, 1382, 1313, 1275, 1261, 1215, 1194, 1118, 1085, 1031, 1019, 834, 763, 749, 698, 633 cm^{-1}; HRMS (FD+) m/z: [M]$^+$ Calcd for $C_{32}H_{26}N_4O_3$, 514.2005; found, 514.2004; HPLC analysis CHIRALPAK IA (hexane/EtOH = 80/20, 1.0 mL/min, 40 °C, 254 nm) 10.1 min (major), 22.7 min (minor). Configuration assignment: The absolute configuration was assigned as (1*R*,2*R*) by analogy with compound **4ac**.

Benzyl 2-((1*R*,2*S*)-2-Benzamido-1-cyano-2-cyclohexylethyl)-1*H*-benzo[d]imidazole-1-carboxylate (4an)

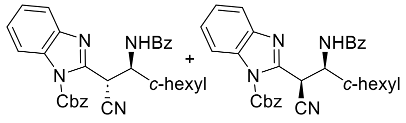

anti-**4an** major *syn*-**4an** minor

Colorless oil (63% combined yield of diastereomers, 0.1 mmol, 30.1 mg, dr = 87/13); R_f = 0.51(hexane/EtOAc= 2/1); $[\alpha]^{23.3}_D$ = -25.8 (c 0.50, $CHCl_3$, mixture of diastereomers (*anti*/*syn* = 29% ee/ 18% ee)); ^{1}H NMR (600 MHz, $CDCl_3$) (for mixture of diastereomers) δ 7.92-7.91 (m, 0.15H (minor)), 7.87 (d, *J* = 7.2 Hz, 0.30H (minor)), 7.80 (dd, *J* = 7.4, 1.5 Hz, 1H), 7.60 (dd, *J* = 7.4, 1.2 Hz, 1H), 7.55-7.52 (m, 2H), 7.48-7.40 (m, 7H), 7.31 (t, *J* = 7.7 Hz, 2H), 7.28-7.23 (m, 3H), 6.17 (d, *J* = 10.7 Hz, 1H), 5.63 (d, *J* = 11.7 Hz, 1H), 5.60 (d, *J* = 11.7 Hz, 0.15H (minor)), 5.55 (d, *J* = 11.7 Hz, 0.15H (minor)), 5.53 (d, *J* = 3.0 Hz, 1H), 5.51 (d, *J* = 11.7 Hz, 1H), 5.38 (d, *J* = 4.1 Hz, 0.15H (minor)), 4.87 (td, *J* = 9.4, 4.4 Hz, 0.15H (minor)), 4.70 (td, *J* = 10.3, 3.1 Hz, 1H), 2.00 (t, *J* = 16.0 Hz, 2H), 1.90-1.85 (m, 2H), 1.79 (d, *J* = 11.3 Hz, 1H), 1.72 (d, *J* = 12.4 Hz, 1H), 1.68-1.58 (m, 0.75H (minor)), 1.45 (m, 0.15H (minor)), 1.41-1.34 (m, 1H), 1.31-1.18 (m, 4H), 1.15-1.08 (m, 2H), 1.06-1.03 (m, 0.30H (minor)); ^{13}C NMR (151 MHz, $CDCl_3$) (for mixture of diastereomers) δ 168.3, 167.3 (minor), 150.6, 150.0 (minor), 147.8, 147.0 (minor), 141.8, 141.3 (minor), 134.3, 133.8, 133.5 (minor), 132.7, 132.2 (minor), 131.7 (minor), 131.5, 129.6 (minor), 129.4, 129.3 (minor), 129.2 (minor), 129.1, 129.0, 128.7 (minor), 128.5, 127.2 (minor), 126.9, 126.5 (minor), 125.8, 125.4 (minor), 124.9, 120.6 (minor), 120.4, 116.5 (minor), 116.4 (minor), 115.5, 70.9 (minor), 70.5, 55.3 (minor), 54.6, 40.9, 39.9 (minor), 37.7, 30.3 (minor), 30.1, 29.5, 29.3 (minor), 26.0, 25.9, 25.7, some carbons were not found due to overlapping; IR (ATR) 3361, 2928, 2853, 1747, 1658, 1580, 1522, 1488, 1454, 1389, 1319, 1297, 1262, 1195, 1122, 1091, 893, 789, 761, 697, 628 cm^{-1}; HRMS (FD+) m/z: $[M+Na]^+$ Calcd for $C_{31}H_{30}N_4O_3$, 529.2210; found, 529.2210; HPLC analysis CHIRALPAK IA (hexane/EtOH = 95/5, 0.5 mL/min, 40 °C, 254 nm) *anti*-**4an**: 24.6 min (minor), 28.4 min (major); *syn*-**4an**: 26.1 min (minor), 47.6 min (major). Configuration assignment: The absolute configuration was assigned as (1*R*,2*S*) by analogy with compound **4ac**.

Benzyl 2-((1*R*,2*S*)-1-Benzamido-3-ethoxy-3-oxo-1-phenylpropan-2-yl)-1*H*-benzo[*d*]imidazole-1-carboxylate (4ca)

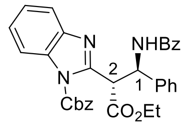

White solid (99% combined yield of diastereomers, 0.2 mmol, 109.5 mg, dr = 80/20); mp 150.4-154.8 °C; R_f = 0.46 (hexane/EtOAc= 2/1); $[\alpha]^{24.6}_D$ = -46.3 (c 3.85, $CHCl_3$, 85% ee); ^{1}H NMR (600 MHz, $CDCl_3$) δ 9.69 (d, *J* = 9.6 Hz, 1H), 7.95 (d, *J* = 7.4 Hz, 2H), 7.81 (d, *J* = 8.2 Hz, 1H), 7.74 (d, *J* = 8.2 Hz, 1H), 7.53-7.51 (m, 1H), 7.49-7.42 (m, 9H), 7.34 (t, *J* = 7.2 Hz, 1H), 7.30 (t, *J* = 7.8 Hz, 1H), 7.19 (t, *J* = 7.4 Hz, 2H), 7.12 (t, *J* = 7.4 Hz, 1H), 6.40 (d, *J* = 9.0 Hz, 1H), 5.45 (d, *J* = 12.0 Hz, 1H), 5.43 (d, *J* = 12.0 Hz, 1H), 5.38-5.37 (m, 1H), 4.23-4.17 (m, 1H), 4.17-4.11 (m, 1H), 1.20 (t, *J* = 7.2 Hz, 3H); ^{13}C NMR (151 MHz, $CDCl_3$) δ 169.1, 166.7, 150.4, 150.2, 141.4, 139.8, 134.6, 133.8, 131.9, 131.4, 129.2, 129.0, 128.9, 128.6, 128.3, 127.2, 127.1, 126.6, 125.4, 124.7, 120.1, 115.2, 70.0, 62.1, 53.6, 52.0, 14.1; IR (ATR) 3325, 3063, 3030, 2982, 1737, 1660, 1602, 1579, 1517, 1485, 1454, 1385, 1319, 1296, 1263, 1208, 1179, 1122, 1087, 1028, 932, 905, 801, 747, 695 cm^{-1}; HRMS (FD+) m/z: $[M]^+$ Calcd for $C_{33}H_{29}N_3O_5$, 547.2107; found, 547.2106; HPLC analysis CHIRALPAK IC-3 (hexane/EtOH = 90/10, 1.0 mL/min, 40 °C, 254 nm) 42.0 min (minor), 52.1 min (major). Configuration assignment: The absolute configuration was assigned as (1*R*,2*S*) by analogy with compound **4ac**.

5. Gram Scale Experiment

Experimental procedure for the Mannich-type reaction of benzimidazole derivative **1a** with imine **1d** catalyzed by (*R*)-**3a** on a gram scale (2.0 mmol).

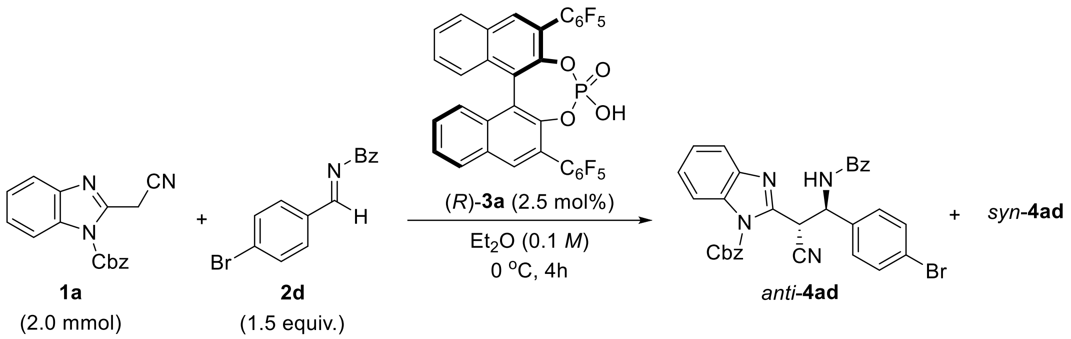

To a mixture of **1a** (583 mg, 2.00 mmol), **2d** (864 mg, 3.00 mmol) and (*R*)-**3a** (34.0 mg, 0.050 mmol) was added Et_2O (20 mL) at 0 °C. The reaction mixture was stirred at 0 °C for 4 h. The mixture was directly passed through short path silica-gel column chromatography (Hexane/EtOAc = 2/1) to remove catalyst (*R*)-**3a**. Diastereomeric ratio was determined by ^{1}H NMR analysis using this mixture. Further purification was performed by flash column chromatography (Hexane/EtOAc = from 4/1 to 1/1), giving **4ad** quantitatively as a white solid (1.159 g, 2.00 mmol). The enantiomeric excess was determined by chiral stationary phase (CHIRALPAK IC-3) HPLC analysis.

6. Control Experiments

Confirmation of retro reaction (Scheme 2b)

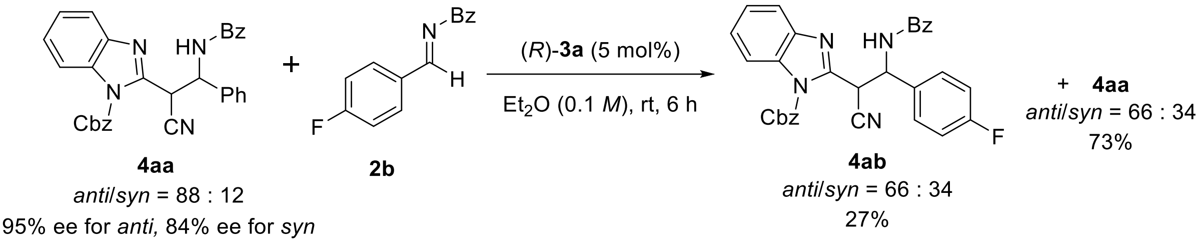

To a mixture of **4aa** (50.0 mg, 0.10 mmol), **2b** (22.7 mg, 0.10 mmol) and (*R*)-**3a** (3.4 mg, 5 μmol) was added Et_2O (1.0 mL) at room temperature. The reaction mixture was stirred at that temperature for 6 h. The mixture was directly passed through short silica-gel column chromatography (Hexane/EtOAc = 2/1) to give a mixture of **4aa** and **4ab** as a colorless oil. The ratio of **4aa** and **4ab** was determined by ^{1}H NMR analysis.

Confirmation of tautomerization (Scheme 2c)

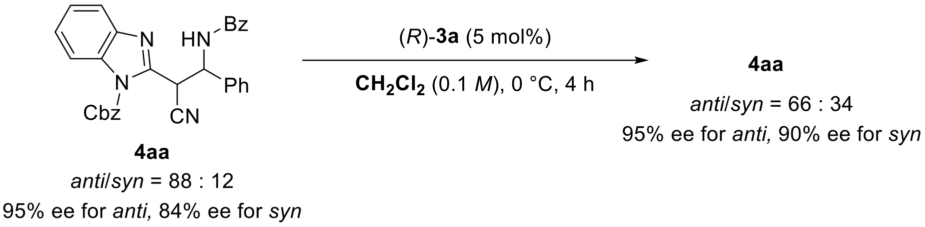

To a mixture of **4aa** (50.0 mg, 0.10 mmol) and (*R*)-**3a** (3.4 mg, 5 μmol) was added dichloromethane (1.0 mL) at 0 °C. The reaction mixture was stirred at 0 °C for 4 h. The mixture was directly passed through short silica-gel column chromatography (Hexane/EtOAc = 2/1) to give a diastereomeric mixture of **4aa** as a colorless oil. The diastereomeric ratio of **4aa** was determined by ^{1}H NMR analysis.

7. Determination of Relative and Absolute Configurations

Preparation of single crystal for X-ray analysis

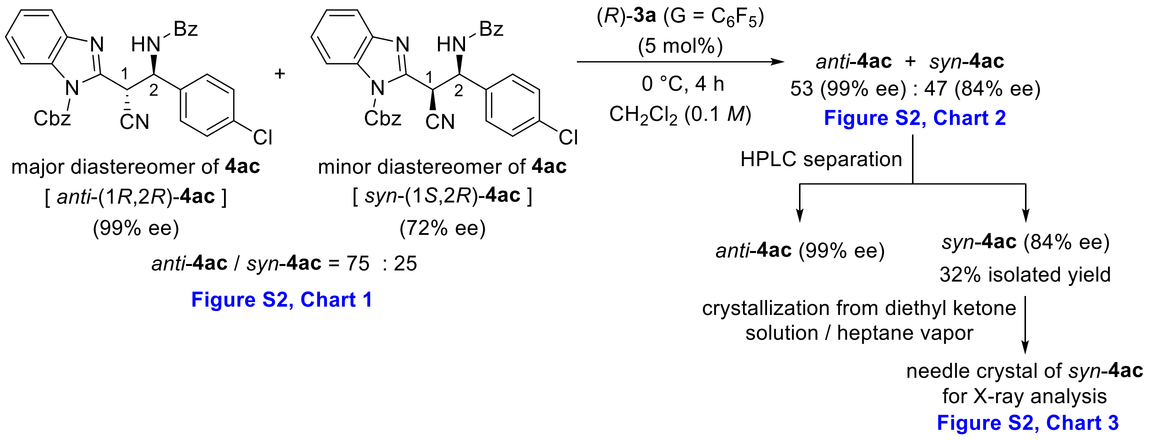

Figure S1. Preparation of the single crystal sample for X-ray crystallographic analysis.

Initially, we tried to crystallize the major diastereomer of **4ac** (*anti*-(1*R*,2*R*)-**4ac**), however all attempts were unsuccessful for generating X-ray grade single crystals. We therefore turned our attention to using the minor diastereomer (*syn*-(1*S*,2*R*)-**4ac**) for crystallization. The preparation of the minor diastereomer (*syn*-(1*S*,2*R*)-**4ac**) was performed in accordance with the following scheme (**Figure S1**). In order to increase the amount of the minor diastereomer (*syn*-(1*S*,2*R*)-**4ac**), we isomerized the major diastereomer of **4ac** (*anti*-(1*R*,2*R*)-**4ac**) using (*R*)-**3a** in dichloromethane at 0 C, according to Scheme 2c. After isomerization, the minor diastereomer (*syn*-(1*S*,2*R*)-**4ac**) was isolated by preparative HPLC. Crystallization of the collected single diastereomer (*syn*-(1*S*,2*R*)-**4ac**) was performed in diethyl ketone under heptane vapor. As a result, a single crystal that can be analyzed by X-ray structure was obtained, although it was a needle-like crystal.

Chart 1: HPLC chart of *anti*-**4ac** / *syn*-**4ac** = 75 : 25

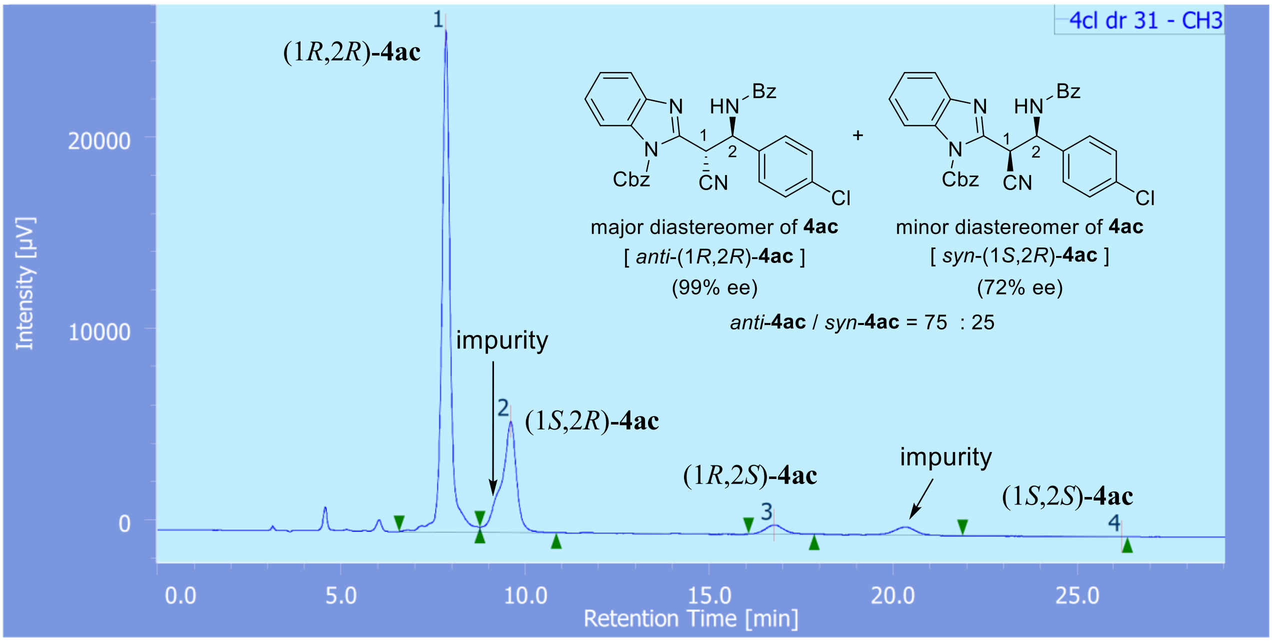

CHIRALPAK IA	Retention time	Area	% Area
anti-(1*R*,2*R*)-**4ac** (major)	7.8	414796	68.900
syn-(1*S*,2*R*)-**4ac** (major)	9.6	166183	27.604
syn-(1*R*,2*S*)-**4ac** (minor)	16.8	18160	3.016
anti-(1*S*,2*S*)-**4ac** (minor)	26.2	2888	0.480

Chart 2: HPLC chart of *anti*-**4ac** / *syn*-**4ac** = 53 : 47

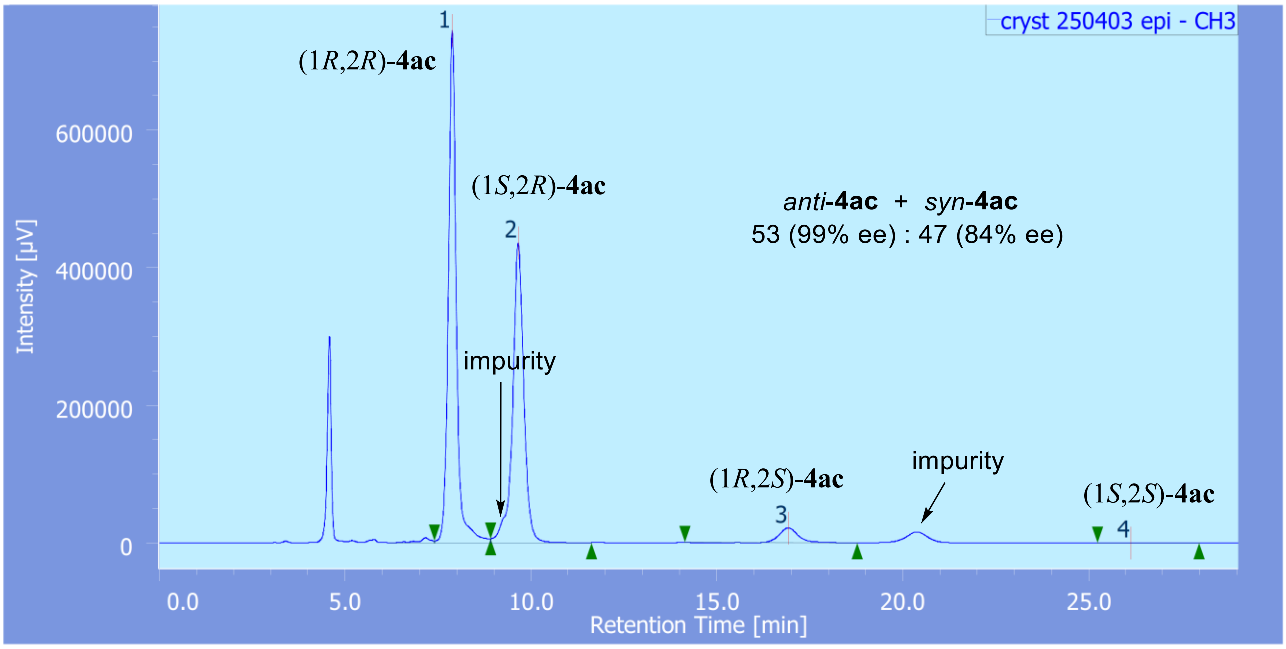

CHIRALPAK IA	Retention time	Area	% Area
anti-(1*R*,2*R*)-**4ac** (major)	7.9	11139424	52.943
syn-(1*S*,2*R*)-**4ac** (major)	9.7	9133718	43.410
syn-(1*R*,2*S*)-**4ac** (minor)	16.9	766908	3.645
anti-(1*S*,2*S*)-**4ac** (minor)	26.1	331	0.002

Chart 3: HPLC chart of the sample *syn*-(1*S*,2*R*)-**4ac** only after crystallization.

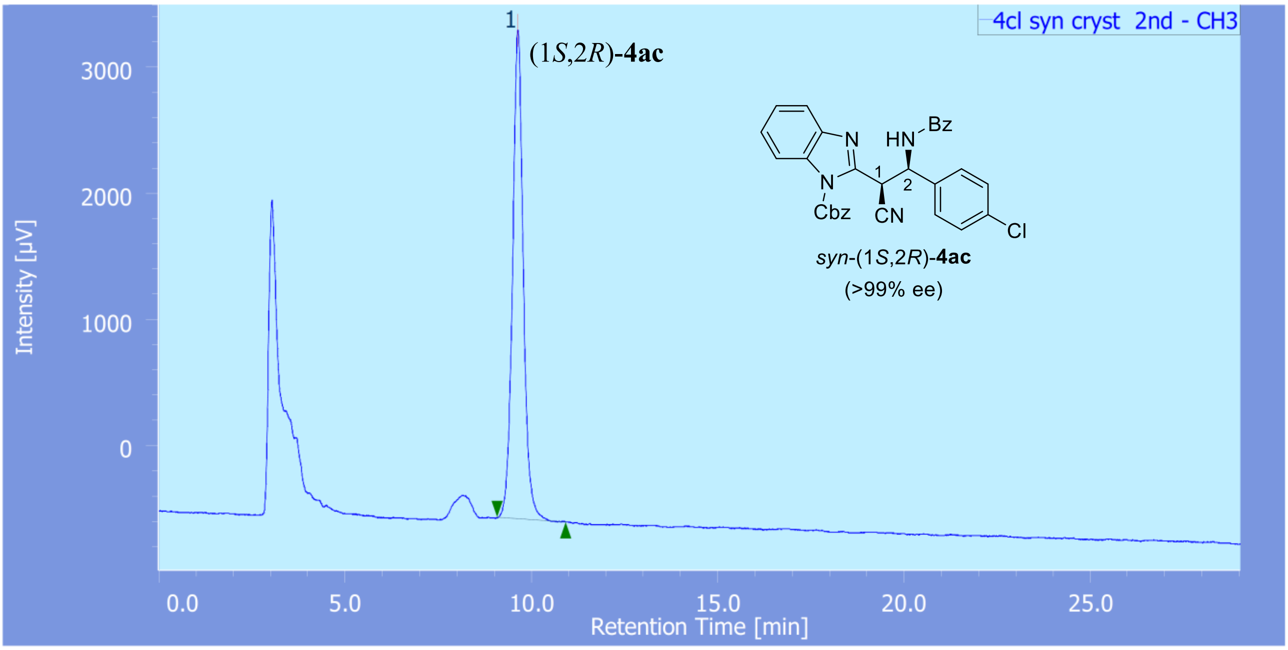

CHIRALPAK IA	Retention time	Area
syn-(1*S*,2*R*)-**4ac** (only)	9.6	77589

Figure S2. HPLC analysis of each step for the preparation of the single crystal X-ray sample. HPLC analysis CHIRALPAK IA (hexane/EtOH = 80/20, 1.0 mL/min, 40 °C, 254 nm) 7.8 min (1*R*,2*R*), 9.6 min (1*S*,2*R*), 16.8 min (1*R*,2*S*), 26.2 min (1*S*,2*S*).

X-ray analysis of **4ac** (minor diastereomer)

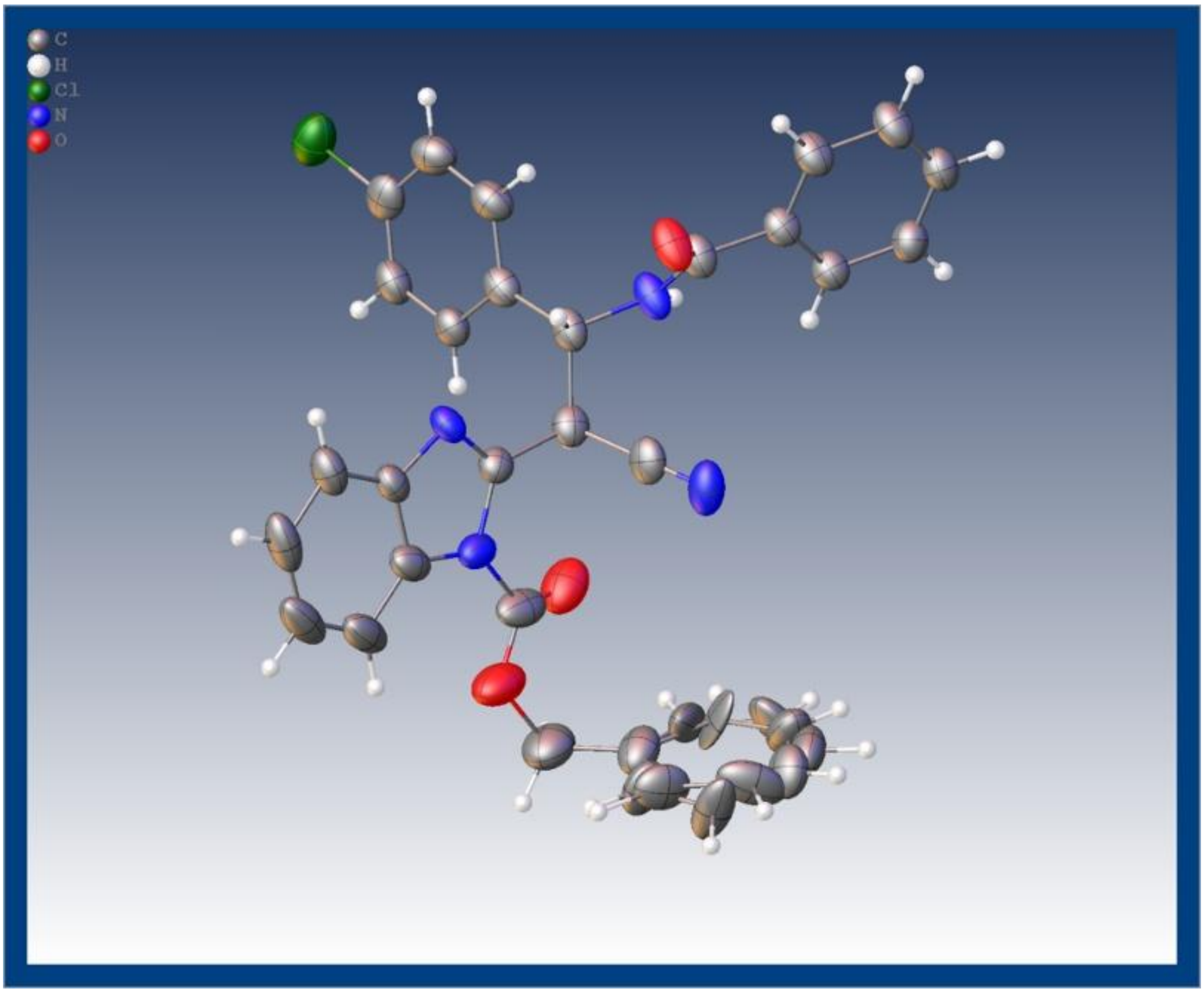

Figure S3. X-ray crystallographic analysis of **4ac** (minor diastereomer). Absolute configuration was determined to be (1*S*,2*R*)-isomer. Therefore, the major diastereomer was assigned to be (1*R*,2*R*)-isomer, because the stereogenic center at the C1 position was epimerized under acidic conditions as shown in **Figure S1**. The ellipsoids were drawn with 30% probability level.

Crystal data and structure refinement for **4ac** (minor diastereomer) (CCDC 2440757)

Identification code	250321-Cu_auto
Empirical formula	$C_{31}H_{23}ClN_4O_3$
Formula weight	534.98
Temperature/K	99.90(14)
Crystal system	monoclinic
Space group	$P2_1$
a/Å	12.2148(5)
b/Å	5.0101(2)
c/Å	21.3442(8)
α/°	90
β/°	101.312(4)

γ/°	90
Volume/Å^3	1280.83(9)
Z	2
ρ_{calc}g/cm^3	1.387
μ/mm^{-1}	1.662
F(000)	556.0
Crystal size/mm^3	0.2 × 0.02 × 0.02
Radiation	Cu Kα (λ = 1.54184)
2Θ range for data collection/°	7.38 to 153.062
Index ranges	$-15 \le h \le 15$, $-6 \le k \le 5$, $-26 \le l \le 26$
Reflections collected	32225
Independent reflections	4960 [R_{int} = 0.1007, R_{sigma} = 0.0465]
Data/restraints/parameters	4960/1/407
Goodness-of-fit on F^2	1.033
Final R indexes [I>=2σ (I)]	R_1 = 0.0658, wR_2 = 0.1633
Final R indexes [all data]	R_1 = 0.0776, wR_2 = 0.1716
Largest diff. peak/hole / e Å^{-3}	0.77/-0.40
Flack parameter	0.066(13)

8. Theoretical Studies

Computational studies were conducted using the Gaussian 16 program package[1] with density functional theory (DFT). Geometry optimizations were performed at the CPCM(ether)/ωB97X-D/def2-SVP level. Single-point energy calculations were subsequently carried out at the SMD(ether)/ωB97M-V/def2-SVPD level using the ORCA program package.[2] Intrinsic reaction coordinate (IRC) calculations were performed for all optimized transition states to confirm their connectivity to the corresponding reactants and products.

(1) Gaussian 16, Revision C.02, M. J. Frisch, G. W. Trucks, H. B. Schlegel, G. E. Scuseria, M. A. Robb, J. R. Cheeseman, G. Scalmani, V. Barone, G. A. Petersson, H. Nakatsuji, X. Li, M. Caricato, A. V. Marenich, J. Bloino, B. G. Janesko, R. Gomperts, B. Mennucci, H. P. Hratchian, J. V. Ortiz, A. F. Izmaylov, J. L. Sonnenberg, D. Williams-Young, F. Ding, F. Lipparini, F. Egidi, J. Goings, B. Peng, A. Petrone, T. Henderson, D. Ranasinghe, V. G. Zakrzewski, J. Gao, N. Rega, G. Zheng, W. Liang, M. Hada, M. Ehara, K. Toyota, R. Fukuda, J. Hasegawa, M. Ishida, T. Nakajima, Y. Honda, O. Kitao, H. Nakai, T. Vreven, K. Throssell, J. A. Montgomery, Jr., J. E. Peralta, F. Ogliaro, M. J. Bearpark, J. J. Heyd, E. N. Brothers, K. N. Kudin, V. N. Staroverov, T. A. Keith, R. Kobayashi, J. Normand, K. Raghavachari, A. P. Rendell, J. C. Burant, S. S. Iyengar, J. Tomasi, M. Cossi, J. M. Millam, M. Klene, C. Adamo, R. Cammi, J. W. Ochterski, R. L. Martin, K. Morokuma, O. Farkas, J. B. Foresman, and D. J. Fox, Gaussian, Inc., Wallingford CT, 2019.

(2) Neese, F. *WIREs Comput. Mol. Sci.* **2022**, *12*, e1606.

Table S6. Energy profile of the Mannich type reaction

	charge	mult.	E_{opt} (au)	G_{corr} (au)	E_{sp} (au)	G (au)	ΔG (kcal/mol)	$\Delta G^{\neq}$ (kcal/mol)
(*R*)-**3a**	0	1	-2863.96698259	0.298220	-2864.264096495489	-2863.965876		
**2a**	0	1	-669.375006380	0.173973	-669.401495935241	-669.2275229		
**1a**	0	1	-969.326123372	0.223699	-969.400927593984	-969.1772286		
sum of **1a** / **2a** / (*R*)-**3a**						-4502.370628	0.0	
**IM1**$_{(RR)}$	0	1	-4502.81078219	0.753711	-4503.159300027009	-4502.405589	-21.9	5.8
**TS**$_{CRR}$	0	1	-4502.80479738	0.757405	-4503.153727365356	-4502.396322	-16.1	--
**PRD**$_{RR}$	0	1	-4502.83433907	0.759791	-4503.183454413563	-4502.423663	-33.3	17.2
**IM2**	0	1	-4502.81421678	0.755159	-4503.165267296610	-4502.410108	-24.8	10.1
**TS**$_{TU}$	0	1	-4502.79478455	0.749571	-4503.143693449151	-4502.394122	-14.7	--
**IM3**	0	1	-4502.79777957	0.752727	-4503.154740785348	-4502.402014	-19.7	5.0
**IM1**$_{(SS)}$	0	1	-4502.80461518	0.755144	-4503.155183094208	-4502.400039	-18.5	4.2
**TS**$_{CSS}$	0	1	-4502.79973286	0.756820	-4503.150173676924	-4502.393354	-14.3	--
**PRD**$_{SS}$	0	1	-4502.82806740	0.756032	-4503.180137948698	-4502.424106	-33.6	19.3

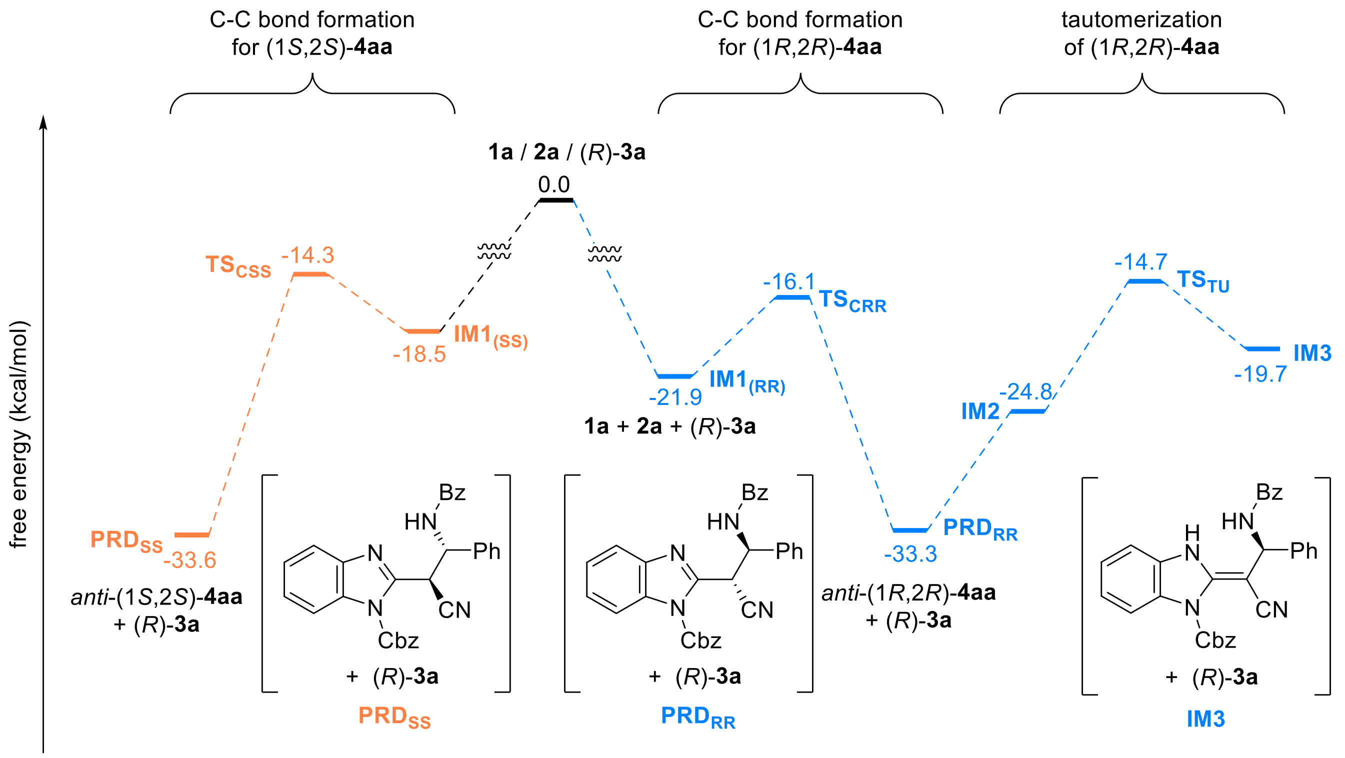

Figure S4. Energy diagram of the reaction of **1a** with **2a** catalyzed by (*R*)-**3a**. The relative free energy (kcal/mol) of the sum of **1a**, **2a**, and (*R*)-**3a** is set to zero.

Cartesian coordinates

IM1$_{(RR)}$

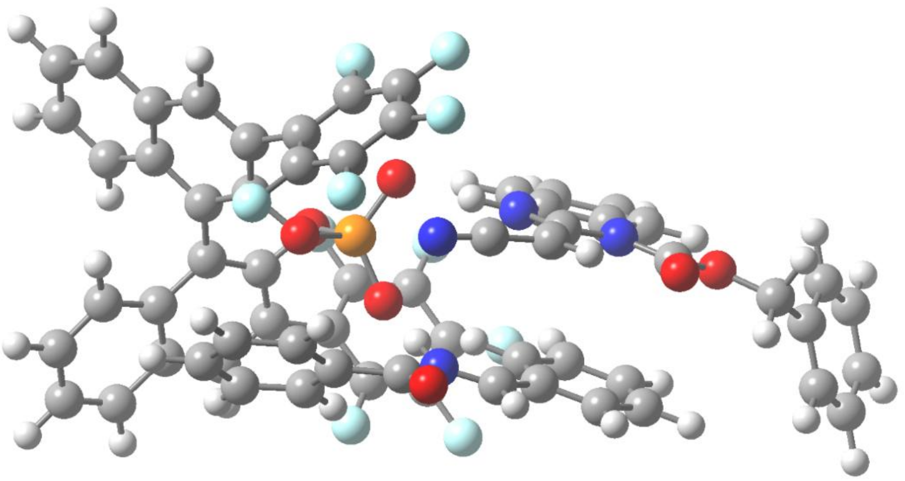

C	2.76418300	0.16278100	-1.90252700	C	5.22287100	2.17383300	-0.19274300
C	4.11299600	0.45133700	-1.66853800	O	6.33009300	1.52608500	-0.51329900
C	5.11325900	-0.35554900	-2.20003100	O	5.16559200	3.12579500	0.54166900
H	6.16495400	-0.15349700	-2.02125400	C	7.55770300	1.95582400	0.09092400
H	0.96209000	1.11790700	-1.30425800	H	7.37110600	2.22485700	1.13941800
N	2.00363600	1.10111800	-1.23737600	H	7.90444800	2.85702600	-0.43747000
N	4.12060400	1.58566500	-0.81157600	C	8.53822600	0.82258500	-0.02895500
C	2.79165900	1.96155200	-0.55093400	C	9.05527900	0.20176100	1.11062400
C	2.35341900	2.96725800	0.28347600	C	8.92681500	0.36015700	-1.29270900
H	3.05693300	3.63546200	0.76793800	C	9.94105800	-0.87081400	0.99341200

H	8.77004300	0.56239000	2.10253500	P	-1.22180100	-0.09936700	-0.53803500
C	9.80352900	-0.71500400	-1.41192700	C	-4.47772300	-0.51309000	-0.84103600
H	8.53671500	0.84671000	-2.19061700	C	-5.63883700	-0.47247400	-1.68250100
C	10.31168000	-1.33383900	-0.26752300	C	-3.73086200	0.64059700	-0.67444600
H	10.33913000	-1.34671500	1.89198200	C	-6.37945900	-1.63925300	-2.02513100
H	10.09670300	-1.07004400	-2.40214300	C	-6.05389500	0.77287500	-2.23568700
H	11.00079700	-2.17581600	-0.36116500	C	-4.13156000	1.88316100	-1.24025900
C	0.97555100	3.08866400	0.53857400	C	-7.48851200	-1.56100900	-2.83235000
N	-0.15965000	3.13476900	0.79393800	H	-6.05325300	-2.60969100	-1.64965300
C	2.78519200	-0.81612600	1.27923200	C	-7.21337900	0.82480300	-3.05767600
C	3.87836600	-1.48331700	0.74639100	C	-5.28413300	1.93762400	-1.98268500
C	5.17202100	-1.17971400	1.17902800	C	-7.92162600	-0.31501000	-3.34638600
C	5.38422900	-0.20027700	2.14905100	H	-8.03752900	-2.47033500	-3.08572800
C	4.30021600	0.48245600	2.68398200	H	-7.52339600	1.79018000	-3.46472500
C	2.99333700	0.17925400	2.25559600	H	-5.60361000	2.89003300	-2.41175800
H	1.77547400	-1.04046000	0.92432000	H	-8.80802000	-0.26714500	-3.98201300
H	3.73643400	-2.23605900	-0.02906500	O	-2.58088100	0.61132100	0.06316000
H	6.02484200	-1.70455900	0.74367800	C	2.36003400	-0.94398100	-2.64005700
H	6.39659700	0.03181900	2.48158500	H	1.29927800	-1.15052300	-2.78838300
H	4.45708400	1.25931200	3.43490700	C	3.36327700	-1.76176300	-3.16143700
C	1.92212100	0.91886300	2.87012200	H	3.08675600	-2.64690500	-3.73557500
H	2.17334200	1.66008800	3.63870300	C	4.71344500	-1.46657300	-2.94861400
C	-0.30893800	1.58042000	3.36821900	H	5.48014300	-2.12096300	-3.36720500
O	0.05914000	2.53512100	3.99613900	O	-0.38753000	-0.53581500	0.64067300
C	-1.69826900	1.08235600	3.28425700	H	0.26410300	0.18590300	1.83801600
C	-2.74451400	2.00797000	3.37243500	N	0.65396700	0.77792800	2.64101500
C	-1.97562300	-0.28735500	3.18172900	C	-6.11589200	-2.08699200	1.13430700
C	-4.06188400	1.56430200	3.34202000	H	-6.51766800	-1.15335300	0.73956300
H	-2.51529900	3.07321100	3.43448700	C	-0.76343200	-3.70576800	-0.11377300
C	-3.29490400	-0.72487500	3.17656300	C	0.17828900	-3.74510800	0.91330700
H	-1.17049800	-1.01844400	3.10623200	C	-0.31189500	-3.93681100	-1.41372000
C	-4.33643500	0.19946500	3.24727000	C	1.52043800	-4.01513300	0.66327900
H	-4.87844400	2.28607900	3.38505300	C	1.02747700	-4.18936700	-1.68907400
H	-3.51195500	-1.79185300	3.10584400	C	1.94547800	-4.23012200	-0.64439000
H	-5.37081000	-0.15031400	3.22356500	C	-3.29918900	3.09530000	-1.02452000
O	-0.61651600	0.69707000	-1.64983200	C	-2.35749400	3.50168500	-1.96760200
C	-6.34137900	-4.02217300	2.57687900	C	-3.44529700	3.86979100	0.12297400
C	-5.09187600	-4.45824800	2.21220300	C	-1.57230300	4.63208300	-1.76457900
C	-4.30042300	-3.71535600	1.29310700	C	-2.67171000	5.00140700	0.34593500
C	-4.82532500	-2.52000300	0.72067100	C	-1.73092500	5.38103000	-0.60408600
C	-6.85071200	-2.81637300	2.03784300	F	-2.20664400	2.82140900	-3.09297800
H	-2.57862000	-5.02990500	1.43859300	F	-0.66346400	4.99006800	-2.66115100
H	-6.93838500	-4.59446700	3.28957000	F	-0.96560000	6.43862900	-0.39167800
H	-4.67882100	-5.37695700	2.63541700	F	-2.80503300	5.69787000	1.46668900
C	-2.97516200	-4.11972700	0.98372000	F	-4.34665300	3.53076300	1.03755400
C	-4.01475700	-1.76878300	-0.19325100	F	-1.16504700	-3.91918400	-2.42487700
H	-7.83511700	-2.45929700	2.34741700	F	1.42821000	-4.40361800	-2.93353000
C	-2.72153900	-2.18815700	-0.42752800	F	3.22360400	-4.46442400	-0.89571500
C	-2.18086000	-3.36601300	0.15652100	F	2.39401800	-4.05444600	1.65872100
O	-1.90327400	-1.43578000	-1.22282800	F	-0.18450100	-3.50508000	2.16622800

TS$_{CRR}$

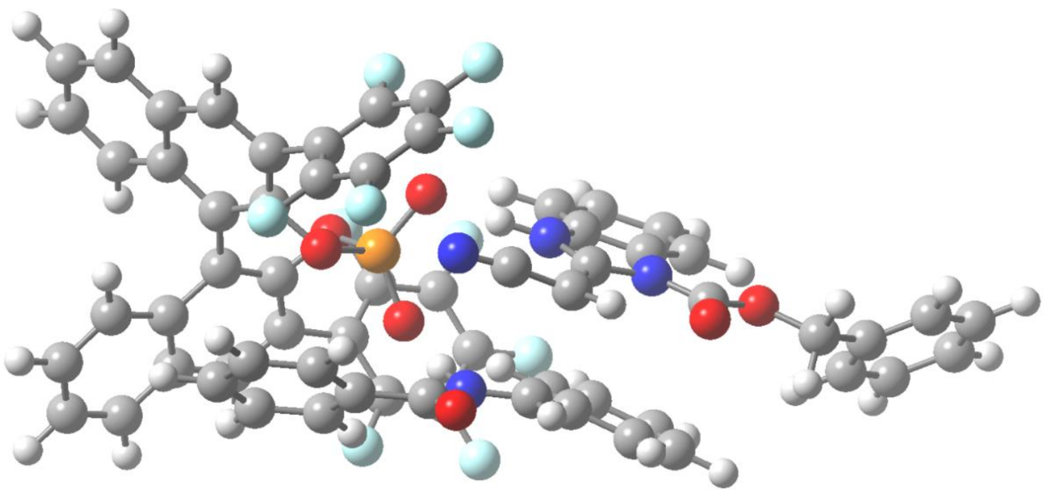

C	2.82652000	-0.01028900	-1.73122700	H	6.33291600	-0.47436400	2.75384500
C	4.15756600	0.22642600	-1.37946000	H	4.51628200	1.11714100	3.32608600
C	5.18158600	-0.56408500	-1.89802900	C	1.99007400	1.02998900	2.57901100
H	6.22327700	-0.39535200	-1.64068900	H	2.28253700	1.83327600	3.26039900
H	0.98686800	0.92065300	-1.17281500	C	-0.21806500	1.60989400	3.26972300
N	2.03919500	0.89261200	-1.04049600	O	0.17312900	2.58670500	3.86276100
N	4.11733700	1.28837800	-0.44070700	C	-1.63361000	1.15456800	3.23389100
C	2.79222500	1.64470800	-0.23543500	C	-2.64118600	2.12423600	3.27630800
C	2.29473400	2.51974200	0.75925800	C	-1.97261300	-0.20354600	3.18551900
H	2.98505300	3.21348800	1.23191000	C	-3.97740800	1.73783300	3.25066700
C	5.20641300	1.91365700	0.19473400	H	-2.36715300	3.18053000	3.29994600
O	6.33517900	1.35716700	-0.18750500	C	-3.31033700	-0.58496200	3.17359700
O	5.10072300	2.82410400	0.97065000	H	-1.19913300	-0.97110600	3.15203300
C	7.54400400	1.83551500	0.42249900	C	-4.31210300	0.38434800	3.19834700
H	7.41899600	1.78708900	1.51536100	H	-4.76132000	2.49617500	3.26170600
H	7.69544600	2.88766000	0.14394000	H	-3.57293700	-1.64399100	3.13324000
C	8.67590000	0.96644200	-0.04906100	H	-5.36057500	0.07937700	3.17334400
C	8.59274800	-0.42639300	0.07060900	O	-0.51107400	0.60579500	-1.61717000
C	9.82857100	1.53607100	-0.59411400	C	-6.49473500	-3.81079100	2.56386200
C	9.64391700	-1.23486800	-0.35349200	C	-5.28199800	-4.31674600	2.16698700
H	7.69209700	-0.87940000	0.49077600	C	-4.46604100	-3.61009900	1.24060400
C	10.88788000	0.72719600	-1.00884400	C	-4.92884700	-2.37761300	0.69523300
H	9.90051100	2.62162100	-0.69850700	C	-6.94099800	-2.56906400	2.05083500
C	10.79629600	-0.65839700	-0.89197700	H	-2.82758200	-5.02898100	1.33088500
H	9.56604400	-2.32010400	-0.26026700	H	-7.11047200	-4.35545700	3.28225800
H	11.78467200	1.18379700	-1.43268000	H	-4.91698700	-5.26455200	2.56966900
H	11.62221400	-1.29197300	-1.22211200	C	-3.17569900	-4.08996900	0.89573300
C	0.93380500	2.88538200	0.66693200	C	-4.09304000	-1.66606300	-0.22750700
N	-0.20938000	3.08786000	0.67129500	H	-7.89519800	-2.15738700	2.38614200
C	2.72968200	-1.06708300	1.39778100	C	-2.83066800	-2.15519900	-0.49014500
C	3.76425900	-1.92606900	1.04442400	C	-2.35003500	-3.37250300	0.06600600
C	5.05655400	-1.71698600	1.52718600	O	-1.98139900	-1.42822200	-1.27903700
C	5.32561600	-0.63423600	2.36394900	P	-1.22108100	-0.16531100	-0.54309800
C	4.30497900	0.24996800	2.69717200	C	-4.50024100	-0.38433300	-0.86009100
C	3.00251700	0.04228700	2.21603900	C	-5.67643500	-0.28625000	-1.67568500
H	1.72128500	-1.23320000	1.00747300	C	-3.69492300	0.73145200	-0.70245200
H	3.57471500	-2.76518100	0.37701200	C	-6.47603300	-1.41626900	-2.00989500
H	5.85560600	-2.40637700	1.24655300	C	-6.05003800	0.98036300	-2.20829700

C	-4.05615400	1.99491100	-1.24937300	C	-0.95376700	-3.78455900	-0.20964800
C	-7.59944200	-1.28175100	-2.78925900	C	-0.04463600	-3.96721200	0.83254000
H	-6.18518300	-2.40351200	-1.64956600	C	-0.47964000	-3.94157100	-1.51339400
C	-7.22529100	1.09151400	-3.00157200	C	1.28251800	-4.30863700	0.59355100
C	-5.22369900	2.10628300	-1.96217100	C	0.85290700	-4.24182300	-1.77603200
C	-7.98968900	-0.01328000	-3.28251500	C	1.73433700	-4.43211600	-0.71682200
H	-8.19398100	-2.16375100	-3.03649200	C	-3.18329900	3.18170200	-1.05124900
H	-7.50209300	2.07356500	-3.39245200	C	-2.20675100	3.52158400	-1.98501300
H	-5.51111900	3.07665600	-2.37340600	C	-3.35136200	4.02014200	0.04699600
H	-8.88818800	0.07916000	-3.89584400	C	-1.41360700	4.65286500	-1.82367500
O	-2.53761400	0.64880500	0.01737400	C	-2.56871600	5.15346300	0.22930800
C	2.44756500	-1.04880300	-2.57606400	C	-1.59665400	5.46893700	-0.71281700
H	1.39566600	-1.21665900	-2.81012900	F	-2.03632900	2.77949700	-3.06795100
C	3.46805000	-1.85049600	-3.07868100	F	-0.47731400	4.95144500	-2.71422000
H	3.21841500	-2.68781100	-3.73151700	F	-0.82881000	6.53278500	-0.54132400
C	4.80859200	-1.60302800	-2.74980400	F	-2.72905100	5.91669800	1.30195700
H	5.58765700	-2.24607900	-3.16296100	F	-4.28685800	3.74867700	0.94953000
O	-0.45146700	-0.67846900	0.64355500	F	-1.30041500	-3.80662800	-2.54197300
H	0.26908500	0.09756200	1.88040000	F	1.28001200	-4.36578900	-3.02436500
N	0.68542200	0.77576900	2.56608500	F	3.00438100	-4.72065000	-0.95495900
C	-6.18222000	-1.87314900	1.14077100	F	2.11950600	-4.49596400	1.60355300
H	-6.53435000	-0.91151600	0.76634400	F	-0.42442200	-3.80342500	2.09205000

PRD$_{RR}$

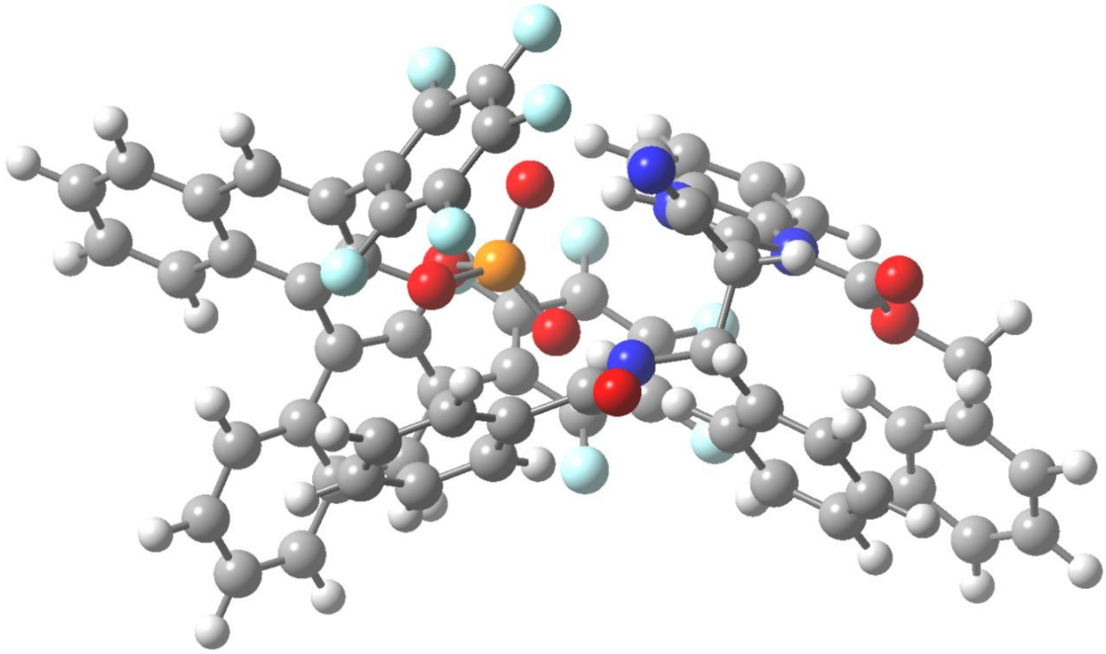

C	2.11960600	0.48015700	-2.55989800	O	5.62386700	1.33011600	-0.69840400
C	3.47409100	0.62735200	-2.25868100	O	4.84320400	3.43298400	-0.52612100
C	4.43886400	-0.19124100	-2.85173900	C	6.89441700	1.63126800	-0.11275700
H	5.49899500	-0.08463000	-2.63483600	H	6.74847000	2.38149700	0.67768400
H	0.35345600	1.43399500	-1.87781400	H	7.53557200	2.06955100	-0.89306000
N	1.43347200	1.46337300	-1.86940200	C	7.48447500	0.36214100	0.44336000
N	3.54334200	1.72137800	-1.37914500	C	6.86768400	-0.87995600	0.28146900
C	2.27774100	2.18313000	-1.16651600	C	8.67915500	0.44497200	1.16660600
C	1.91087100	3.30347400	-0.23306400	C	7.43662100	-2.02468000	0.84049900
H	2.73388400	4.03107800	-0.27345000	H	5.92493700	-0.95903800	-0.25957100
C	4.73504500	2.27857000	-0.81657800	C	9.24715700	-0.69914200	1.72208500

H	9.16627300	1.41419200	1.30412700	C	-4.09598500	-1.47604200	-0.23164900
C	8.62573400	-1.93918100	1.56173000	C	-5.37329500	-1.91060100	-0.71654800
H	6.93571700	-2.98646400	0.71263600	C	-3.75424400	-0.14224300	-0.35431200
H	10.17813200	-0.62148100	2.28764300	C	-5.75381900	-3.28188700	-0.74842700
H	9.06829900	-2.83548300	2.00134700	C	-6.29281700	-0.94396200	-1.21735000
C	0.70043100	3.97362600	-0.70958200	C	-4.66207900	0.82545700	-0.86730600
N	-0.25279500	4.51284100	-1.07176400	C	-6.98900300	-3.66122600	-1.21561800
C	3.01211500	0.73185400	1.68402500	H	-5.04959100	-4.04044300	-0.40441000
C	4.10635000	0.03972100	2.19842500	C	-7.56802100	-1.36626500	-1.68439500
C	5.13869700	0.72832200	2.83517600	C	-5.91109000	0.42257500	-1.26601700
C	5.07136100	2.11495300	2.96064400	C	-7.91370500	-2.69479500	-1.67956500
C	3.97384500	2.80883100	2.45133000	H	-7.25810200	-4.71938600	-1.23480600
C	2.93858400	2.12551600	1.80822200	H	-8.26547300	-0.61212400	-2.05662800
H	2.20692300	0.17560300	1.19406100	H	-6.61864800	1.16058000	-1.65043400
H	4.15082900	-1.04411900	2.09681200	H	-8.89351800	-3.01039300	-2.04337800
H	5.99863200	0.18140100	3.22752600	O	-2.52358000	0.28972900	0.05075000
H	5.87277700	2.66078800	3.46317800	C	1.64568000	-0.49593300	-3.43714300
H	3.92178300	3.89537800	2.55538500	H	0.57817200	-0.59275000	-3.63454500
C	1.73715000	2.89279100	1.28258100	C	2.60236800	-1.30776800	-4.02549200
H	1.66757300	3.85196500	1.81464100	H	2.28931800	-2.08949900	-4.71847000
C	-0.40710800	2.70958000	2.42113800	C	3.97274500	-1.15120300	-3.73992700
O	-0.35880200	3.85312600	2.84867500	H	4.69277400	-1.81321600	-4.22243600
C	-1.47210600	1.74374500	2.85205900	O	-0.01041300	-0.14434100	0.21024900
C	-2.73744200	2.24349000	3.17123100	H	0.34237200	1.27882400	1.12402200
C	-1.21286200	0.37543700	2.99676500	N	0.50377100	2.20942900	1.53718300
C	-3.74333000	1.38057500	3.59904400	C	-4.60059100	-3.07105700	2.25942100
H	-2.92966000	3.31253100	3.06147800	H	-5.37147100	-2.38786800	1.90209400
C	-2.21680500	-0.48407000	3.43365000	C	0.51369800	-3.34990800	-0.32586600
H	-0.22784300	-0.02788100	2.76132500	C	1.66292400	-2.94773300	0.35526900
C	-3.48496000	0.01587400	3.72829400	C	0.66921700	-3.82513300	-1.62894200
H	-4.73567300	1.77421300	3.82575600	C	2.92194100	-3.03821200	-0.23148900
H	-2.01118600	-1.55154300	3.53975800	C	1.91990700	-3.92782800	-2.22886200
H	-4.27299600	-0.66390700	4.05999600	C	3.05227200	-3.53805800	-1.52164200
O	-1.01804700	0.74837900	-1.99976400	C	-4.22765500	2.24412300	-0.94585800
C	-3.81381500	-4.66794300	3.90486000	C	-3.59453500	2.74621700	-2.08177700
C	-2.61701700	-4.78463800	3.24258900	C	-4.41555600	3.10762500	0.13001400
C	-2.36499600	-4.03790400	2.05873200	C	-3.13593100	4.05844800	-2.13479100
C	-3.37845300	-3.18157100	1.53996400	C	-3.94955400	4.41791800	0.10522500
C	-4.81015200	-3.79264700	3.40978800	C	-3.30953200	4.89369600	-1.03504300
H	-0.33221800	-4.75975100	1.81994400	F	-3.43681300	1.97678400	-3.14662600
H	-3.99589500	-5.23804500	4.81793600	F	-2.53169600	4.51445600	-3.21993300
H	-1.83304400	-5.44318100	3.62386500	F	-2.87450200	6.13847500	-1.07507600
C	-1.10183200	-4.09945000	1.41425400	F	-4.11807200	5.21109800	1.15454000
C	-3.10559500	-2.41414700	0.35987700	F	-5.06213600	2.68676800	1.20879100
H	-5.75199500	-3.68428300	3.95158300	F	-0.39124100	-4.21515100	-2.31943300
C	-1.84367200	-2.47515800	-0.19450400	F	2.04041400	-4.39791000	-3.46169400
C	-0.82401800	-3.32221700	0.31732700	F	4.25081400	-3.64747300	-2.06786100
O	-1.52860300	-1.63653800	-1.23585800	F	4.00897600	-2.66987200	0.43636600
P	-1.15127400	-0.10500400	-0.76214500	F	1.58547500	-2.47981100	1.59236900

IM2

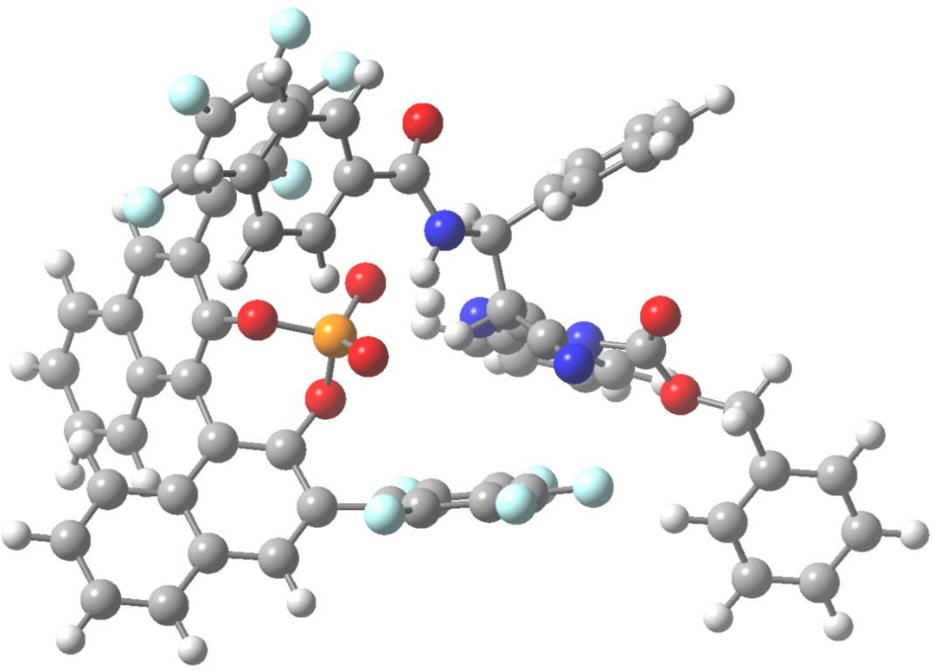

C	-2.33397600	-0.31358000	2.56331400	C	3.95378800	5.05590600	-2.90559600
C	-3.67875700	-0.14199300	2.22417300	H	2.48630700	5.90685600	-1.56233400
C	-4.70217500	-0.62533700	3.04182300	C	4.42329900	3.88816800	-3.50813200
H	-5.75089500	-0.50569200	2.78018600	H	4.15134500	1.75630900	-3.72840400
N	-1.58670700	0.26622700	1.55612800	H	4.46551000	6.00492300	-3.07738800
N	-3.67786600	0.55977100	1.00416200	H	5.30407400	3.92215600	-4.15294600
C	-2.38802700	0.77470300	0.63928700	C	-1.93715500	-0.98076100	3.72235600
C	-1.78227100	1.39981800	-0.58334100	H	-0.88251100	-1.11236700	3.96344700
H	-0.97205600	0.69944300	-0.87757200	C	-2.95175600	-1.46439600	4.53364900
C	-4.83711200	1.03473800	0.30399200	H	-2.69617400	-1.99695600	5.45102900
O	-5.82132100	0.19840600	0.50290400	C	-4.30823700	-1.28475100	4.19800800
O	-4.83911200	2.04061600	-0.33434600	H	-5.07618700	-1.68224900	4.86363800
C	-7.07070500	0.46059100	-0.15951400	H	0.19464700	2.17748300	-1.84661600
H	-6.85910700	0.61362500	-1.22889500	N	-0.00783100	2.94075100	-1.20727300
H	-7.50033800	1.38923800	0.24204500	H	-0.51117000	0.32544900	1.45615200
C	-7.97184000	-0.72053400	0.06994900	O	0.92319700	0.55031700	1.00056400
C	-7.46227500	-2.02358100	0.02265200	C	4.58461400	-6.06500200	-2.94871500
C	-9.33624400	-0.52491200	0.29832500	C	3.23756100	-5.80082000	-2.96233400
C	-8.31042400	-3.11382800	0.20317800	C	2.68060700	-4.80739100	-2.11022600
H	-6.39598500	-2.18201700	-0.14785000	C	3.53240600	-4.09863000	-1.21118600
C	-10.18603800	-1.61826100	0.46784000	C	5.43717600	-5.33389000	-2.08730400
H	-9.74040100	0.48956500	0.34595200	H	0.66499600	-5.01701000	-2.89151700
C	-9.67444300	-2.91426200	0.42263100	H	5.00289900	-6.82637400	-3.60995800
H	-7.90354900	-4.12672300	0.16964900	H	2.57252300	-6.34314200	-3.63852900
H	-11.25106400	-1.45438800	0.64473700	C	1.29822500	-4.48619500	-2.17752600
H	-10.33796800	-3.77042100	0.56174800	C	2.96119700	-3.10906400	-0.34390800
C	-2.60735900	1.46835200	-1.78689400	H	6.51164400	-5.52810300	-2.09789800
N	-3.14304400	1.50043200	-2.80918100	C	1.61640300	-2.82646000	-0.47176500
C	-1.05949700	2.74192600	-0.23402900	C	0.76494800	-3.49499300	-1.39248500
C	0.99957800	3.81444800	-0.92068300	O	1.06835100	-1.83079600	0.28667800
O	0.93166800	4.58683900	0.02180200	P	1.33531100	-0.27859500	-0.19334600
C	2.17741600	3.79816800	-1.84971900	C	3.75577200	-2.32007100	0.64079400
C	2.64675900	2.62850700	-2.45840400	C	4.49697300	-2.93165200	1.70672800
C	2.84719100	5.00783500	-2.06435200	C	3.72052100	-0.93907400	0.57690100
C	3.77650800	2.67585300	-3.27452500	C	4.48756500	-4.33603800	1.93868300
H	2.15529500	1.66784000	-2.28630100	C	5.24017000	-2.10630000	2.60146800

C	4.43512500	-0.10703600	1.47849100	C	4.96138000	3.44587500	0.25734500
C	5.20959900	-4.88735400	2.96949800	C	4.07377200	4.15244700	1.06395300
H	3.89011000	-4.98085100	1.29343900	H	-0.58830800	2.58829200	0.74949700
C	5.98756200	-2.70808500	3.65140300	C	-2.03740800	3.89206900	-0.11661000
C	5.19760600	-0.69417900	2.45585300	C	-2.63452400	4.15168300	1.11924200
C	5.97986000	-4.06903600	3.83011100	C	-2.40073100	4.65393000	-1.22832100
H	5.18412100	-5.96715800	3.13004200	C	-3.59552000	5.15293700	1.24210400
H	6.56114600	-2.06324200	4.32137700	H	-2.33782900	3.57268400	1.99885600
H	5.76072500	-0.06913900	3.15241400	C	-3.35785400	5.65948700	-1.10564300
H	6.55374400	-4.52139100	4.64127300	H	-1.92435300	4.46094900	-2.19207400
O	2.96113500	-0.31545200	-0.37945400	C	-3.96042300	5.90840500	0.12775000
O	0.73399000	-0.00664200	-1.53971600	H	-4.05411200	5.35014700	2.21341800
C	4.92725200	-4.37678500	-1.24340200	H	-3.63424600	6.25335100	-1.97948500
H	5.59883800	-3.81424900	-0.59380100	H	-4.70978900	6.69728900	0.22217200
C	-0.63681500	-3.02033100	-1.51781800	F	-1.15098400	-3.59076600	0.71251600
C	-1.08391200	-2.43045300	-2.70082400	F	-3.61805900	-2.53187900	0.51561500
C	-1.51744400	-3.04292300	-0.43510100	F	-4.41632200	-1.35955000	-1.78815500
C	-2.35714400	-1.87553100	-2.81240500	F	-2.75360100	-1.33011600	-3.94708500
C	-2.79062000	-2.48994200	-0.52241100	F	-0.28688400	-2.36608400	-3.75362800
C	-3.20704800	-1.89692000	-1.71122000	F	2.74802800	1.49219300	3.09763700
C	4.31432900	1.36902900	1.34175000	F	2.47335200	4.14994000	2.78768600
C	3.44801900	2.10101000	2.15308300	F	3.97809400	5.46358500	0.94406800
C	5.05165400	2.06413500	0.38509600	F	5.72209700	4.09070600	-0.61315700
C	3.30749900	3.47885400	2.01045300	F	5.87714000	1.40609300	-0.41867300

TS$_{TU}$

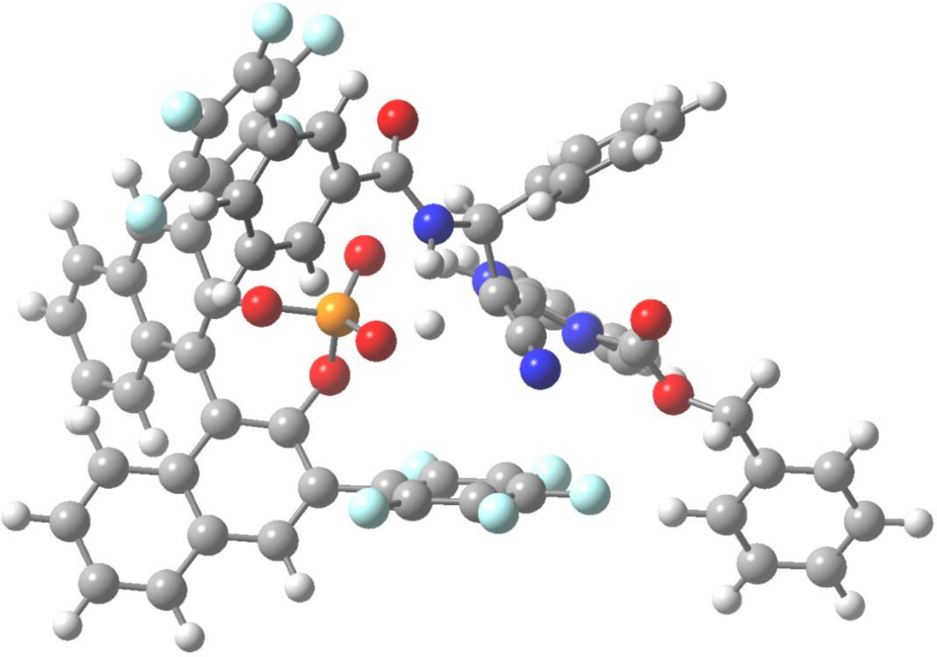

C	-2.45909800	-0.05043800	2.74169900	O	-5.71450100	0.26350500	0.27090900
C	-3.73520100	-0.06228500	2.17590400	O	-4.68455000	2.13299200	-0.42427900
C	-4.81652500	-0.62969800	2.84780100	C	-6.92534800	0.59689400	-0.42047900
H	-5.81231800	-0.65230600	2.41202500	H	-6.69491500	0.65739200	-1.49559700
N	-1.62006600	0.57587500	1.83634700	H	-7.27062900	1.58721500	-0.09086700
N	-3.60821600	0.59121600	0.92728200	C	-7.94101600	-0.47291200	-0.12813900
C	-2.29439800	0.94490500	0.74461500	C	-7.55950600	-1.81664800	-0.03782400
C	-1.63731800	1.53780900	-0.39861300	C	-9.28779200	-0.13154600	0.02235000
H	-0.49886600	0.61750900	-0.77148500	C	-8.51553100	-2.80184900	0.20144800
C	-4.70749500	1.09800400	0.16876300	H	-6.50853800	-2.08808000	-0.14776700

C	-10.24504000	-1.12009800	0.25073800	C	4.37468000	-4.49942400	1.91787300
H	-9.59288300	0.91644000	-0.03670000	C	5.29390100	-2.29802900	2.45891700
C	-9.86053500	-2.45711300	0.34273300	C	4.49176200	-0.28035300	1.36102500
H	-8.20747000	-3.84702900	0.27553700	C	5.16044000	-5.06702100	2.89141700
H	-11.29465400	-0.84163700	0.36621700	H	3.69081700	-5.12367500	1.34154900
H	-10.60839700	-3.23106100	0.52823600	C	6.10454400	-2.91703100	3.45019400
C	-2.31697900	1.55437200	-1.65655700	C	5.31488100	-0.89058100	2.27421700
N	-2.72320300	1.54261600	-2.74445200	C	6.04476800	-4.27220100	3.65980500
C	-0.83499400	2.82338900	-0.07577700	H	5.09840800	-6.14098700	3.07828900
C	1.31497400	3.72148200	-0.81137800	H	6.76920000	-2.29061500	4.04949900
O	1.48602800	4.30825600	0.24629800	H	5.98266800	-0.28263000	2.88827000
C	2.33376700	3.72041000	-1.91066500	H	6.66741900	-4.73834500	4.42586500
C	2.65100700	2.54536500	-2.60182000	O	2.77415900	-0.45114400	-0.29851200
C	2.99793600	4.91159000	-2.21671300	O	0.42978200	0.03151100	-1.05467900
C	3.62342800	2.56926900	-3.60032000	C	4.61139600	-4.55193200	-1.27821800
H	2.15728000	1.60552500	-2.34163200	H	5.33647800	-4.02472100	-0.65787900
C	3.94277600	4.93770000	-3.23846600	C	-0.88560200	-2.94648000	-1.38162200
H	2.76173900	5.81437700	-1.65053400	C	-1.25830700	-2.34516600	-2.58649500
C	4.25832900	3.76670700	-3.92841200	C	-1.82758300	-2.97136800	-0.35371300
H	3.88480100	1.64738900	-4.12366700	C	-2.52315000	-1.79887000	-2.77767200
H	4.44908500	5.87258200	-3.48704900	C	-3.09936400	-2.43017700	-0.52310300
H	5.01225800	3.78549700	-4.71833500	C	-3.44194300	-1.83550500	-1.73428600
C	-2.19323300	-0.60977100	3.98877800	C	4.47422500	1.19462500	1.18395200
H	-1.18735200	-0.59798000	4.40860100	C	3.86541400	2.03537800	2.11563900
C	-3.26902800	-1.18034200	4.65868100	C	5.08876400	1.78286100	0.08033500
H	-3.11002700	-1.63445400	5.63817600	C	3.86373100	3.41713900	1.95350500
C	-4.55649200	-1.18402400	4.09747900	C	5.13218800	3.16296600	-0.07964500
H	-5.37873500	-1.64052800	4.65107900	C	4.50210400	3.98036700	0.85311800
H	0.05877100	2.62612700	-2.00382300	H	-0.30709800	2.65971000	0.87514900
N	0.18561400	3.01862300	-1.07872000	C	-1.76037100	4.01696900	0.10195800
H	-0.57631800	0.59312000	1.87139200	C	-2.34268800	4.24944900	1.35160100
O	1.02539800	0.43802800	1.43802700	C	-2.09994200	4.84185500	-0.97181500
C	4.12333600	-6.20540300	-2.98320400	C	-3.26178400	5.28233200	1.52337400
C	2.78885000	-5.88961600	-2.93120100	H	-2.07028700	3.61878000	2.20295400
C	2.31316300	-4.88616400	-2.04219500	C	-3.01802800	5.87766100	-0.80147800
C	3.23045100	-4.22455100	-1.17436800	H	-1.64165400	4.67484300	-1.94914400
C	5.04289300	-5.51665900	-2.15616300	C	-3.60427200	6.09837100	0.44449000
H	0.26962500	-4.96914400	-2.76287300	H	-3.70680500	5.45580100	2.50563000
H	4.48098200	-6.97378700	-3.67126000	H	-3.27669200	6.51632700	-1.64891200
H	2.07196900	-6.39537600	-3.58211200	H	-4.32267700	6.91024800	0.57700400
C	0.94992900	-4.49406500	-2.05298000	F	-1.53244700	-3.53402000	0.80762700
C	2.73282500	-3.23339400	-0.26456700	F	-3.99524200	-2.50330400	0.45204200
H	6.10754300	-5.75011000	-2.22094000	F	-4.64900400	-1.31949900	-1.90147600
C	1.39421900	-2.90275100	-0.31503000	F	-2.85391000	-1.25929500	-3.93488900
C	0.48654900	-3.49422700	-1.23269500	F	-0.39643800	-2.27412200	-3.58883100
O	0.91448500	-1.92796400	0.52952400	F	3.28088200	1.52479100	3.18919000
P	1.23590900	-0.36852200	0.20188300	F	3.26649300	4.19488900	2.84025700
C	3.62226500	-2.47200800	0.65510900	F	4.51905100	5.29049600	0.69594500
C	4.43117100	-3.10165000	1.65810100	F	5.76955500	3.69805000	-1.10814800
C	3.64303900	-1.09390600	0.56815300	F	5.66800300	1.01934500	-0.83767200

IM3

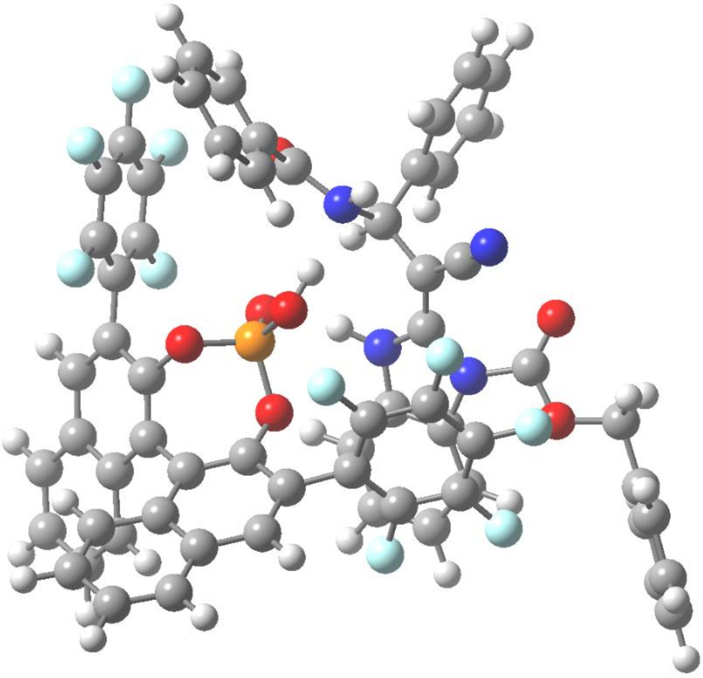

C	-1.87303100	-0.04144300	2.33754500
C	-3.15272600	0.13865100	1.80552400
C	-4.18244300	-0.74319700	2.10876000
H	-5.18188600	-0.61398200	1.70342700
N	-1.07011000	0.99260800	1.88161800
N	-3.08177100	1.31221900	1.00039200
C	-1.75461500	1.76413600	0.98832900
C	-1.20045900	2.74958800	0.20024500
H	0.90817300	1.44046400	-1.11151700
C	-4.18626300	2.12367400	0.68182500
O	-5.26386700	1.36975000	0.56112800
O	-4.14639300	3.31497200	0.55527200
C	-6.51278400	2.03534900	0.31321500
H	-6.47318600	2.49343900	-0.68441200
H	-6.63730600	2.83412700	1.05850900
C	-7.59556200	1.00024600	0.41421400
C	-8.18869700	0.47053900	-0.73386500
C	-7.98689100	0.52157700	1.66991300
C	-9.16342900	-0.52170800	-0.63051200
H	-7.87727600	0.82995600	-1.71713800
C	-8.95352400	-0.47576100	1.77519100
H	-7.52613600	0.93235700	2.57233600
C	-9.54461300	-0.99811600	0.62313700
H	-9.62271700	-0.92836800	-1.53399800
H	-9.25144700	-0.84407900	2.75920500
H	-10.30540200	-1.77754400	0.70445300
C	-1.86404700	3.30309000	-0.92103000
N	-2.27608500	3.77498900	-1.90080300
C	0.18895400	3.30607900	0.47071800
C	2.43366900	3.31516200	-0.51547800
O	2.91196300	3.43148600	0.59130100
C	3.26348000	3.34883300	-1.75810900
C	2.90582300	2.67986500	-2.93567900
C	4.46683700	4.06095600	-1.70393900
C	3.74920700	2.72577500	-4.04388200
H	1.98782000	2.09269800	-2.99528000
C	5.30048000	4.11362600	-2.81550700
H	4.73856800	4.56751400	-0.77747000
C	4.94352500	3.44341300	-3.98598800
H	3.47316900	2.19106900	-4.95456200
H	6.23769200	4.67076900	-2.76520300
H	5.60156400	3.47644900	-4.85683000
C	-1.56059600	-1.11785300	3.15624600
H	-0.54950300	-1.25281300	3.54170200
C	-2.58844600	-2.01655700	3.44751700
H	-2.37999300	-2.88041600	4.08150400
C	-3.87566600	-1.82500000	2.94009200
H	-4.66381700	-2.53996000	3.18225200
H	0.68914100	3.46833700	-1.57262900
N	1.06193600	3.10811600	-0.69392800
H	-0.04797200	0.90112100	1.84831900
O	1.50276500	0.32580600	1.17921400
C	0.96929500	-7.15965900	-2.91230500
C	-0.07785300	-6.27272900	-2.90659700
C	-0.01862100	-5.08447300	-2.12727600
C	1.12698800	-4.82784100	-1.32101400
C	2.12802800	-6.88581300	-2.14670200
H	-1.93683800	-4.32564300	-2.80342300
H	0.91905600	-8.06748500	-3.51648300
H	-0.96884600	-6.45969600	-3.51028900
C	-1.06723400	-4.13073400	-2.17212700
C	1.16537000	-3.64018400	-0.51456200
H	2.97113000	-7.57893800	-2.17612100
C	0.14107000	-2.72155300	-0.64810500
C	-0.98904100	-2.94921000	-1.47752500
O	0.16039600	-1.54331700	0.06839700
P	1.29615100	-0.40575500	-0.09519600
C	2.29710200	-3.37060300	0.41560000
C	2.66318600	-4.26606900	1.47679400
C	2.99913000	-2.19268200	0.29318800
C	1.91946400	-5.43934100	1.78168900
C	3.78960600	-3.94703700	2.29082500

C	4.10241800	-1.84117400	1.11249400	C	5.93351700	1.09994700	-0.43903200
C	2.30139400	-6.26810300	2.80906000	C	5.89943500	2.02228800	0.60255000
H	1.02961800	-5.67716400	1.19812200	H	0.65969900	2.74165400	1.28390300
C	4.16826400	-4.83311300	3.33676500	C	0.10469600	4.76002700	0.92296500
C	4.49684500	-2.73372000	2.07765600	C	-0.20486600	5.02224300	2.26185500
C	3.44512200	-5.97243000	3.58827500	C	0.26848100	5.83172500	0.04363100
H	1.71317300	-7.16103000	3.02989000	C	-0.34693000	6.33094200	2.71415300
H	5.04014600	-4.58231800	3.94517700	H	-0.33579700	4.18900700	2.95778700
H	5.34668400	-2.49603900	2.72064400	C	0.12591600	7.14502700	0.49511300
H	3.74037700	-6.64400500	4.39669700	H	0.51280700	5.65867500	-1.00686500
O	2.56284600	-1.24986400	-0.62478800	C	-0.18204900	7.39875900	1.82968900
O	0.89826300	0.46308000	-1.33525500	H	-0.58350700	6.51958300	3.76354400
C	2.20699800	-5.75325300	-1.37317200	H	0.25764400	7.97294600	-0.20476800
H	3.11310800	-5.55471800	-0.80030100	H	-0.29128700	8.42639200	2.18265300
C	-2.05720200	-1.92329700	-1.61847500	F	-3.50210100	-3.07849700	-0.16167300
C	-1.89152800	-0.84645900	-2.48968400	F	-5.50495200	-1.34236300	-0.62764100
C	-3.28738600	-2.05651400	-0.97660200	F	-5.09507900	0.79849200	-2.26139300
C	-2.90083000	0.08890500	-2.69527300	F	-2.72471000	1.09177000	-3.53637900
C	-4.32721000	-1.15731300	-1.20010100	F	-0.77230500	-0.72915500	-3.18551200
C	-4.12151000	-0.06978800	-2.04384300	F	4.11605800	0.15757300	3.11232200
C	4.74079300	-0.51248100	0.93934600	F	5.21975500	2.58700100	2.77854800
C	4.70506600	0.43900200	1.95988800	F	6.46642500	3.20681500	0.45589500
C	5.35584900	-0.15289100	-0.26024700	F	6.53181000	1.40770100	-1.57890500
C	5.27007800	1.69976300	1.79999800	F	5.40790800	-1.02082500	-1.26107200

IM1$_{(SS)}$

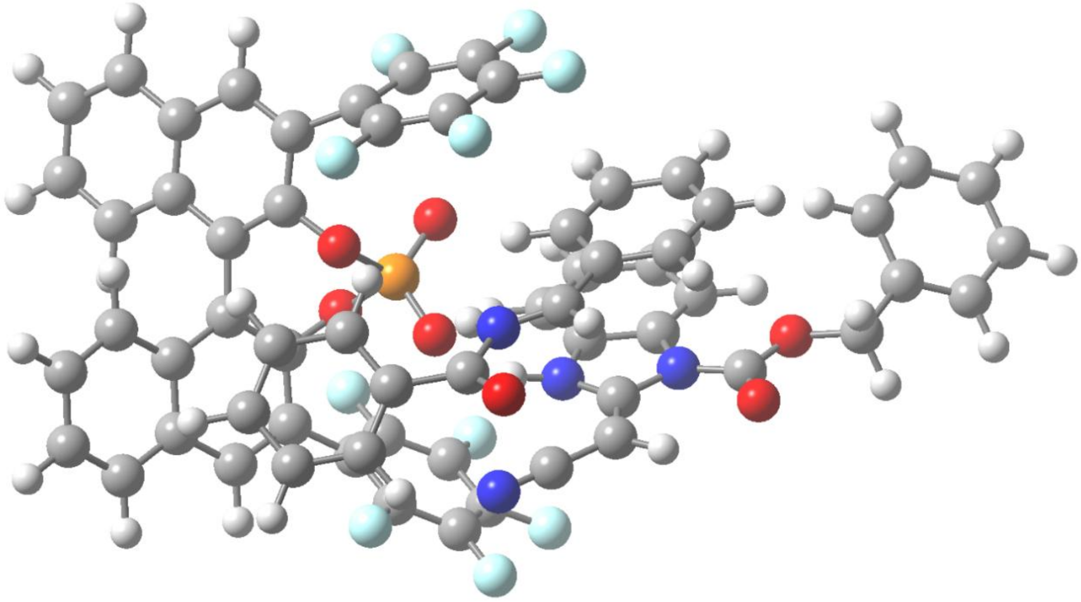

C	2.49942600	-0.96468100	-1.46724300
C	3.87876100	-0.85012600	-1.26264100
C	4.69776300	-0.26523600	-2.22170100
H	5.77077500	-0.17740100	-2.07922500
H	0.95371100	-1.49862200	-0.10379600
N	1.95195700	-1.58718400	-0.35506300
N	4.13110800	-1.40331500	0.02226900
C	2.92276200	-1.87704000	0.55320900
C	2.74061000	-2.50760600	1.76155800
H	3.58276000	-2.67986400	2.42324300
C	5.33572600	-1.41262100	0.72442700
O	6.29660300	-0.84895700	0.01208800
O	5.47505200	-1.86085400	1.83297800
C	7.58549700	-0.74315000	0.62515900
H	8.01391000	-1.74879300	0.74381700
H	7.46012500	-0.31182900	1.63067400
C	8.45236900	0.12636200	-0.24341500
C	9.77542000	-0.23584500	-0.50741200
C	7.95629000	1.32555400	-0.76803600
C	10.59427300	0.59082400	-1.27792300
H	10.17102900	-1.17421200	-0.11034600
C	8.76912900	2.14612800	-1.54612800
H	6.92035800	1.60997600	-0.57385300
C	10.09262300	1.78167800	-1.80038200
H	11.62680500	0.29652800	-1.47736700
H	8.36835000	3.07619300	-1.95479100
H	10.73099900	2.42541300	-2.40917800
C	1.47366600	-3.01241200	2.10765900
N	0.42586500	-3.41004600	2.41960400
C	2.03838000	1.38834300	0.90185500
C	2.90611000	2.06901000	0.06259300
C	4.23108400	2.27610900	0.45015400
C	4.69482300	1.80105700	1.67966700
C	3.83004200	1.12158900	2.52783300
C	2.49094600	0.90462300	2.14378900
H	1.01487800	1.24508600	0.56643300
H	2.53952400	2.43350700	-0.89789800
H	4.90673500	2.82061200	-0.21285000
H	5.73097100	1.96928900	1.97833600
H	4.18788100	0.74198000	3.48695700
C	1.65153900	0.17853200	3.06031400
H	2.04659200	-0.05437800	4.05681300
C	-0.27328200	-0.95697200	3.90206400
O	0.31340800	-1.28096000	4.89629800
C	-1.70077700	-1.20587400	3.60576500
C	-2.27708000	-2.42419800	3.98814000
C	-2.47466200	-0.22833900	2.97647500
C	-3.61431400	-2.67094200	3.69830600
H	-1.65737500	-3.18224700	4.46833900
C	-3.81874200	-0.47207100	2.71059600
H	-2.04413000	0.73141700	2.69703500
C	-4.38540000	-1.69674900	3.05671400
H	-4.05914400	-3.63115100	3.96603000
H	-4.41717900	0.29472900	2.21578000
H	-5.43317500	-1.89598800	2.81798100
O	-0.55099600	-0.60259000	0.37459200
C	-7.12375400	3.62748300	-3.04713800
C	-6.10665200	4.21502600	-2.33596400
C	-5.16099200	3.42533000	-1.62576700
C	-5.29075900	2.00347800	-1.63443600
C	-7.23056800	2.21711100	-3.08874600
H	-3.96981800	5.12387800	-0.97422500
H	-7.84195600	4.24364200	-3.59163300
H	-6.00201000	5.30236600	-2.31358900
C	-4.07121200	4.03698900	-0.94616700
C	-4.34215500	1.21099100	-0.90547200
H	-8.02550000	1.75208600	-3.67544100
C	-3.29946000	1.86265900	-0.28053300
C	-3.13535200	3.27592600	-0.29531600
O	-2.34546400	1.13143400	0.36360800
P	-1.17789300	0.42169000	-0.55639200
C	-4.37853700	-0.27895300	-0.84194100
C	-5.49076700	-1.00255500	-0.29756400
C	-3.25647000	-0.99107700	-1.22687100
C	-6.61802400	-0.35349100	0.27939100
C	-5.44395700	-2.42648800	-0.26329100

C	-3.18769200	-2.41265200	-1.17244400	C	-1.89040500	3.79877700	0.32440400
C	-7.65106400	-1.07925000	0.82268800	C	-0.86278900	4.31942700	-0.46022000
H	-6.65300600	0.73627800	0.30502100	C	-1.61656300	3.56920900	1.67296900
C	-6.53839200	-3.15068100	0.28483300	C	0.39726600	4.58082900	0.07103200
C	-4.28472000	-3.10442400	-0.72103200	C	-0.36110300	3.80090900	2.22018500
C	-7.62158500	-2.49465400	0.81482500	C	0.64940900	4.31643300	1.41242100
H	-8.50097200	-0.55874000	1.26878600	C	-1.90746500	-3.08114000	-1.51642800
H	-6.49153800	-4.24213100	0.29335200	C	-1.26917700	-3.91857100	-0.59603100
H	-4.25813600	-4.19452900	-0.67122300	C	-1.25126200	-2.84074700	-2.72628100
H	-8.45253000	-3.05866700	1.24285700	C	-0.01472900	-4.46691100	-0.85212400
O	-2.14329200	-0.32360200	-1.65692000	C	0.01319400	-3.35676200	-2.98961000
C	1.88632500	-0.48808900	-2.61767000	C	0.63791800	-4.16139700	-2.04246100
H	0.80901400	-0.55838100	-2.75827600	F	-1.82197800	-2.09978300	-3.66368400
C	2.70459800	0.10880900	-3.57664700	F	0.62292300	-3.08675800	-4.13422100
H	2.25524800	0.49703900	-4.49231900	F	1.84363200	-4.64660200	-2.27908200
C	4.08416500	0.21279700	-3.38314400	F	0.56708500	-5.27149100	0.02081100
H	4.70402800	0.67712900	-4.15223600	F	-1.85396200	-4.20585200	0.55510000
O	-0.34219900	1.41941100	-1.27378300	F	-1.05212800	4.52909500	-1.75201800
H	-0.01142400	-0.21593900	1.89714200	F	1.37165500	5.03718000	-0.70284000
N	0.43205700	-0.22726100	2.85694800	F	1.85151700	4.53495200	1.91652000
C	-6.33880900	1.42673900	-2.40389500	F	-0.11596800	3.52116700	3.49270600
H	-6.42753800	0.34085600	-2.45493000	F	-2.55725700	3.05930500	2.45910300

TS$_{CSS}$

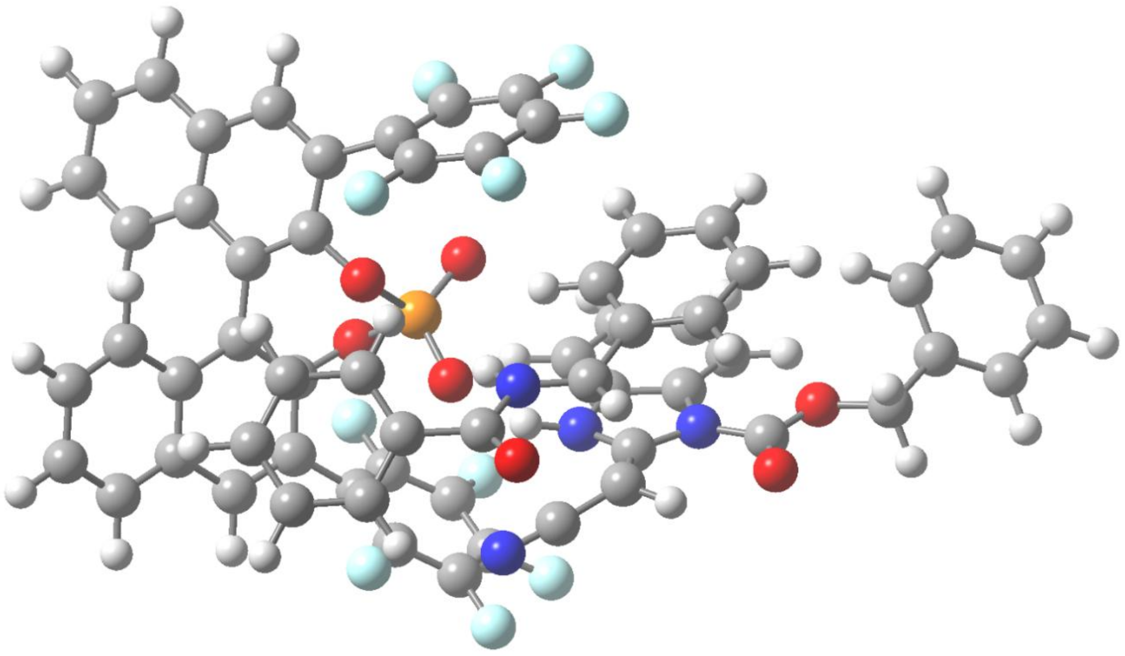

C	2.49953200	-1.06286000	-1.45427700	C	5.29066500	-1.38298600	0.83140300
C	3.87014700	-0.92273000	-1.21884900	O	6.25493000	-0.86407000	0.10411000
C	4.71671400	-0.41533400	-2.20199200	O	5.40449600	-1.80064700	1.95129100
H	5.78464600	-0.30544300	-2.03911800	C	7.54504600	-0.72441600	0.71660700
H	0.90782100	-1.49092200	-0.09027500	H	7.97736400	-1.72253800	0.87455900
N	1.92707200	-1.58448100	-0.30246900	H	7.40914300	-0.25347700	1.70256500
N	4.07907600	-1.36755000	0.11229300	C	8.40251500	0.11591100	-0.18858100
C	2.86390800	-1.76890600	0.63895600	C	9.72004300	-0.25950900	-0.46085600
C	2.60762800	-2.18572000	1.96055400	C	7.90021800	1.29934500	-0.74320400
H	3.45762700	-2.37555900	2.61075400	C	10.52895700	0.53976600	-1.26981700

H	10.11914200	-1.18602400	-0.04024700	P	-1.16337900	0.36132400	-0.64018600
C	8.70346300	2.09146000	-1.55966800	C	-4.39084900	-0.25778300	-0.79079500
H	6.86815000	1.59466000	-0.54238400	C	-5.49771400	-0.94465500	-0.19098600
C	10.02197900	1.71470400	-1.82214300	C	-3.30168700	-1.00176300	-1.20586500
H	11.55755300	0.23630100	-1.47552500	C	-6.59059600	-0.25880400	0.40883000
H	8.29910500	3.00930500	-1.99166300	C	-5.47968300	-2.36826200	-0.12731100
H	10.65257100	2.33682500	-2.46075800	C	-3.26058600	-2.42288800	-1.12184600
C	1.42976200	-2.93374600	2.18744600	C	-7.61975400	-0.94997400	1.00225800
N	0.43401400	-3.48962500	2.40415200	H	-6.60216100	0.83175900	0.40988600
C	2.07571400	1.34749700	0.85332600	C	-6.56894300	-3.05628900	0.47500400
C	2.87807600	2.14388700	0.04562900	C	-4.35257800	-3.08112300	-0.61220200
C	4.19565000	2.41158400	0.41510900	C	-7.61957100	-2.36548600	1.02607300
C	4.71341800	1.87876700	1.59829700	H	-8.44324600	-0.40163800	1.46427500
C	3.92056000	1.06538600	2.39986700	H	-6.54466700	-4.14806100	0.50689300
C	2.59250500	0.78681700	2.03106400	H	-4.34606600	-4.17013400	-0.53776000
H	1.05631600	1.15611500	0.52864300	H	-8.44673600	-2.90191000	1.49494500
H	2.46122800	2.55307100	-0.87581500	O	-2.19460200	-0.36608500	-1.69456400
H	4.81938200	3.04628400	-0.21786200	C	1.91064300	-0.68722600	-2.65570200
H	5.74077300	2.09796600	1.89600600	H	0.83650100	-0.76725700	-2.81303300
H	4.33227200	0.62734000	3.31149100	C	2.75409600	-0.17456900	-3.63621400
C	1.82814300	-0.11792900	2.88074600	H	2.33212800	0.13425300	-4.59390000
H	2.26082700	-0.40298100	3.84354200	C	4.13087600	-0.04769600	-3.41259200
C	-0.17958600	-0.95840100	3.86121800	H	4.76794900	0.35372900	-4.20258700
O	0.41217000	-1.32661500	4.84522300	O	-0.31313100	1.30971600	-1.40555200
C	-1.63502200	-1.13582300	3.62042200	H	0.01550100	-0.19850000	1.90038100
C	-2.24976000	-2.32617300	4.02760000	N	0.50919700	-0.28572600	2.81188900
C	-2.38768800	-0.13621200	3.00035600	C	-6.36455400	1.45907800	-2.31399700
C	-3.60285300	-2.52515500	3.77331800	H	-6.47711200	0.37456300	-2.34123700
H	-1.64890400	-3.10189200	4.50436100	C	-1.79461900	3.79850900	0.23742000
C	-3.74595500	-0.33188800	2.76616200	C	-0.77864400	4.31248000	-0.56603000
H	-1.92728900	0.80374100	2.70086200	C	-1.51327900	3.61195500	1.59082100
C	-4.35172700	-1.52978300	3.13897200	C	0.47511600	4.61868000	-0.04404100
H	-4.07719900	-3.46474200	4.06327700	C	-0.26292700	3.88798600	2.12947000
H	-4.32572600	0.45184300	2.27500900	C	0.73382600	4.40365600	1.30513000
H	-5.41151400	-1.69143700	2.92654900	C	-2.00551500	-3.12061200	-1.49749600
O	-0.54889700	-0.67297800	0.28797400	C	-1.34076800	-3.93807500	-0.57843000
C	-7.12743300	3.66367100	-2.97086300	C	-1.39240500	-2.91619600	-2.73596700
C	-6.07423700	4.24274300	-2.30697500	C	-0.09866500	-4.50067900	-0.86376800
C	-5.12015300	3.44632000	-1.61572000	C	-0.14099000	-3.44697900	-3.03007100
C	-5.27797300	2.02780600	-1.59321400	C	0.51196500	-4.23193400	-2.08526800
C	-7.26403000	2.25518000	-2.98178000	F	-1.99165300	-2.19533000	-3.67065100
H	-3.87848800	5.13082600	-1.02547400	F	0.43166300	-3.20736300	-4.20067300
H	-7.85201300	4.28455300	-3.50137800	F	1.70403500	-4.73512300	-2.35367100
H	-5.94704400	5.32790500	-2.30835000	F	0.51006800	-5.28668000	0.00776800
C	-3.99732400	4.04616200	-0.98194500	F	-1.88633100	-4.18888600	0.59974600
C	-4.31979500	1.22856300	-0.88464400	F	-0.97649500	4.48509600	-1.86241300
H	-8.08858200	1.79610900	-3.53110800	F	1.43750300	5.07638500	-0.83272100
C	-3.23852300	1.86695000	-0.31224600	F	1.92910400	4.67114000	1.80048000
C	-3.05368700	3.27663400	-0.35225000	F	-0.01363500	3.65920200	3.41021800
O	-2.27204100	1.12999200	0.30641700	F	-2.44470800	3.11212400	2.39417400

PRD$_{SS}$

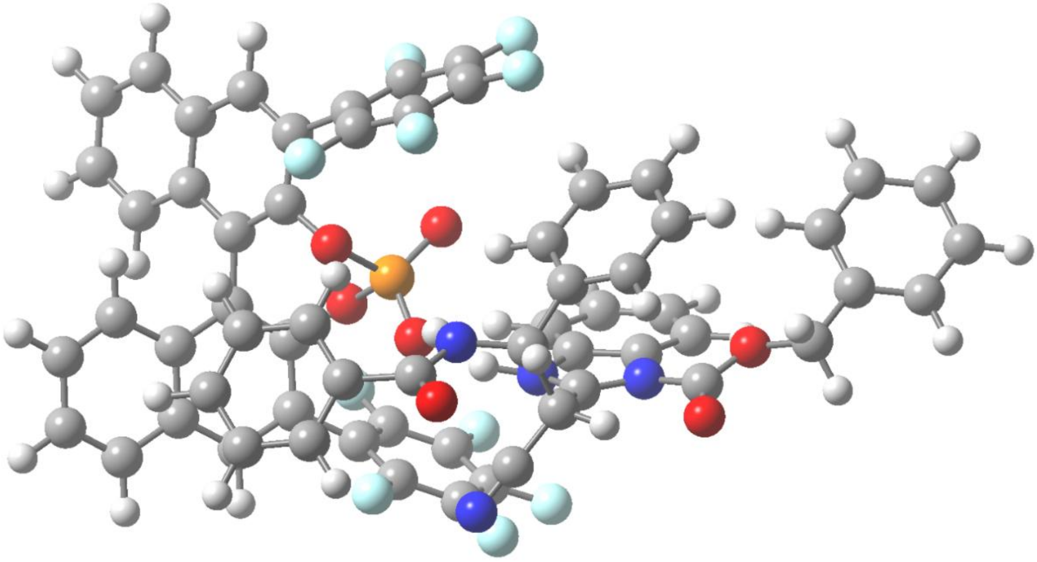

C	2.39009200	-1.37281400	-1.32793500	H	4.85466500	3.34607300	-0.05368900
C	3.77673200	-1.36017400	-1.16262000	H	5.81611000	2.35663100	2.02221600
C	4.63508200	-1.24305100	-2.25938500	H	4.47389600	0.77882900	3.38425800
H	5.71590000	-1.23633400	-2.14638700	C	2.01898200	-0.21659500	2.86023600
H	0.77392500	-1.32921400	0.12185000	H	2.32323800	-0.19820000	3.91481000
N	1.82578100	-1.53335800	-0.07180500	C	-0.10090700	-0.54889100	3.97690600
N	3.99285200	-1.50698700	0.22194600	O	0.44908700	-0.80656800	5.03380300
C	2.77670100	-1.60228000	0.83289700	C	-1.57565700	-0.70227200	3.77199700
C	2.54321500	-1.62314100	2.31787700	C	-2.20901800	-1.83246700	4.30075200
H	3.51213400	-1.82058000	2.79133400	C	-2.31480500	0.23492100	3.04836500
C	5.24707100	-1.42287600	0.90382000	C	-3.56753500	-2.03690200	4.07310100
O	6.12521600	-0.85629100	0.12223100	H	-1.62106700	-2.55814300	4.86611600
O	5.41942600	-1.80904000	2.02342900	C	-3.67756800	0.03737200	2.83877900
C	7.43283200	-0.58947100	0.65715500	H	-1.83216600	1.12422600	2.64552300
H	7.94752400	-1.54424200	0.83443300	C	-4.30327000	-1.10252600	3.34041400
H	7.30384000	-0.08339500	1.62653700	H	-4.05602300	-2.93166000	4.46478900
C	8.17134200	0.27242200	-0.32853500	H	-4.24778400	0.77327700	2.26790500
C	9.54365000	0.09246600	-0.52003800	H	-5.36691100	-1.26685600	3.15204500
C	7.50630800	1.28387500	-1.03091200	O	-0.56066100	-0.71502600	0.31954700
C	10.24678100	0.92016200	-1.39546200	C	-7.20396300	3.26376200	-3.20003800
H	10.06916500	-0.70167800	0.01656600	C	-6.14737700	3.92209900	-2.62095600
C	8.20794400	2.10327100	-1.91267600	C	-5.16191200	3.21182700	-1.88145500
H	6.43233800	1.42428800	-0.89126200	C	-5.29171100	1.79979500	-1.71998200
C	9.58027700	1.92573800	-2.09440300	C	-7.31323700	1.85834500	-3.07361900
H	11.31914300	0.77134000	-1.53774500	H	-3.94236200	4.96585800	-1.47538600
H	7.67918400	2.88647600	-2.46003800	H	-7.95296300	3.81837200	-3.76865600
H	10.12941000	2.56949400	-2.78473000	H	-6.04218900	5.00427100	-2.72824100
C	1.65322400	-2.71830500	2.70350300	C	-4.03771100	3.88736100	-1.33286300
N	0.95078500	-3.56733300	3.04651900	C	-4.30246700	1.08837800	-0.96324600
C	2.20241800	1.45299900	0.94090200	H	-8.14118600	1.33489300	-3.55616800
C	2.96260200	2.32964000	0.16859900	C	-3.21749100	1.79441700	-0.48460800
C	4.26310500	2.65447600	0.54967900	C	-3.06452500	3.19660900	-0.65799200
C	4.80143900	2.09876200	1.71060300	O	-2.22031100	1.13711200	0.17822500
C	4.04308900	1.21743900	2.48042100	P	-1.13341700	0.27016700	-0.69438300
C	2.74007100	0.87770400	2.09730300	C	-4.34457600	-0.38167400	-0.72386000
H	1.19503300	1.21003300	0.60316600	C	-5.41447400	-1.02832700	-0.02026800
H	2.52836800	2.75681700	-0.73696500	C	-3.25501300	-1.14062300	-1.10319100

C	-6.51484200	-0.31087300	0.52582100	H	-6.47431900	0.06142600	-2.27654600
C	-5.34960200	-2.43488200	0.20254500	C	-1.81775000	3.80655400	-0.13053200
C	-3.17347300	-2.54214700	-0.87309200	C	-0.80772800	4.24669900	-0.98321200
C	-7.50943200	-0.96017100	1.21745700	C	-1.55482700	3.80955400	1.23906600
H	-6.56086000	0.77196900	0.40508600	C	0.42158300	4.67408800	-0.48915700
C	-6.40041100	-3.08027800	0.91032600	C	-0.32944700	4.21401100	1.75384000
C	-4.21834400	-3.16875600	-0.24333300	C	0.66040300	4.65850000	0.88094100
C	-7.46164300	-2.36243700	1.40386300	C	-1.92586200	-3.24133100	-1.27203800
H	-8.34086900	-0.38745200	1.63328500	C	-1.06024600	-3.77468100	-0.31613200
H	-6.34044500	-4.15993300	1.06614100	C	-1.51894400	-3.27389000	-2.60638800
H	-4.17370300	-4.24306300	-0.05303700	C	0.17989500	-4.30005200	-0.67346800
H	-8.26047300	-2.86602800	1.95159900	C	-0.28513700	-3.79273300	-2.98358300
O	-2.17443000	-0.53185200	-1.68679000	C	0.56920200	-4.29908800	-2.00923300
C	1.78662300	-1.23251100	-2.57798900	F	-2.30116800	-2.77005000	-3.54841600
H	0.70464600	-1.19551200	-2.68822000	F	0.09850700	-3.77046800	-4.25099000
C	2.63632800	-1.11291000	-3.66546300	F	1.76197900	-4.75557500	-2.34818800
H	2.21161100	-1.00384300	-4.66424500	F	1.01073200	-4.76983500	0.24433000
C	4.03471500	-1.12597400	-3.50573200	F	-1.39033500	-3.75410500	0.96318500
H	4.67081400	-1.03180000	-4.38737200	F	-0.99099200	4.24043200	-2.29342400
O	-0.23153500	1.10828100	-1.52287900	F	1.38035000	5.06289600	-1.31889700
H	0.08199000	-0.17567600	1.97035000	F	1.82941600	5.05370600	1.35188000
N	0.58775800	-0.13878400	2.86168900	F	-0.09964000	4.17856000	3.05685700
C	-6.38324500	1.14546900	-2.35574100	F	-2.48198900	3.38193600	2.08691400

9. NMR Spectra

^{1}H NMR (600 MHz, $CDCl_3$) and ^{13}C NMR (151 MHz, $CDCl_3$) spectra of *anti*-**4aa**

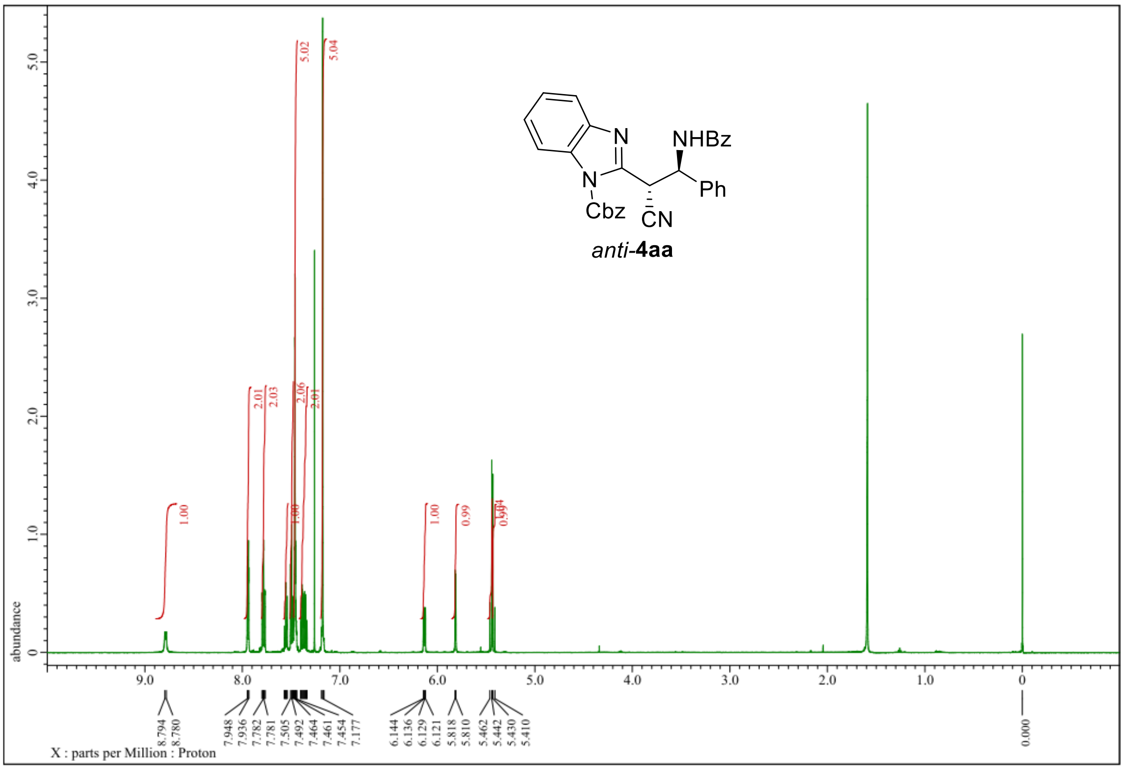

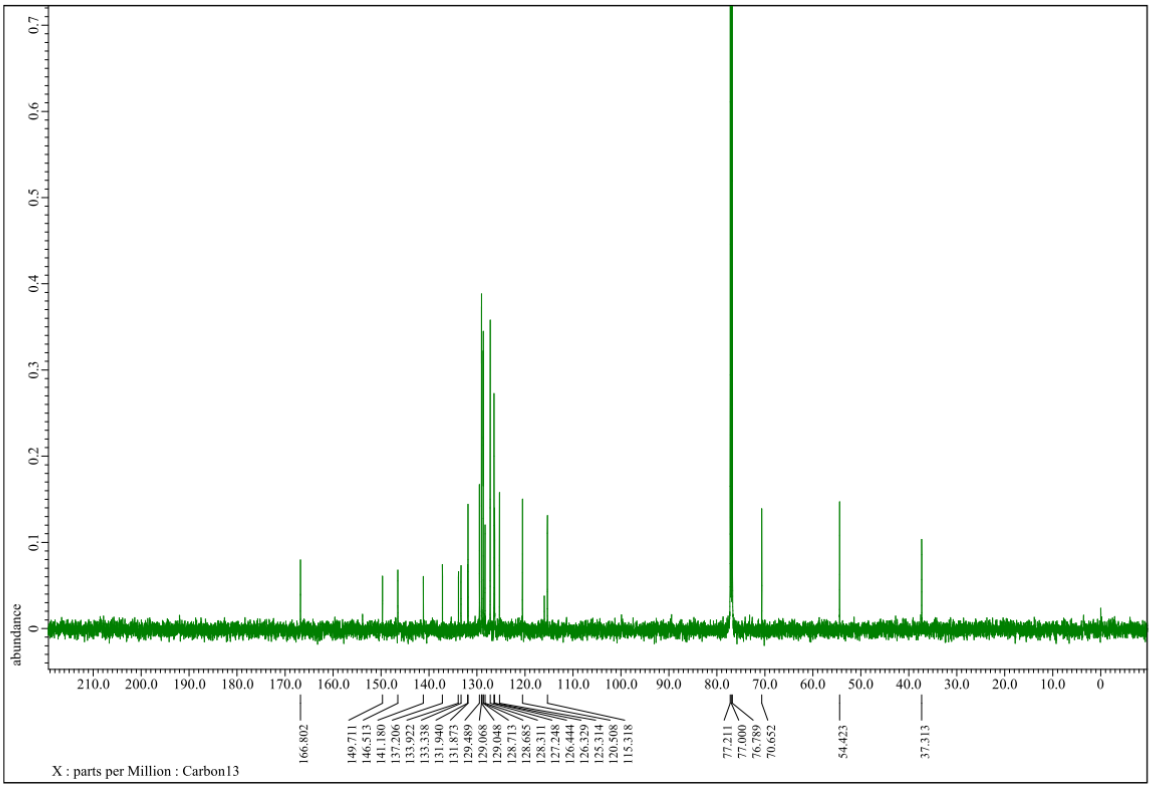

^{1}H NMR (600 MHz, $CDCl_3$) and ^{13}C NMR (151 MHz, $CDCl_3$) spectra of *syn*-**4aa**

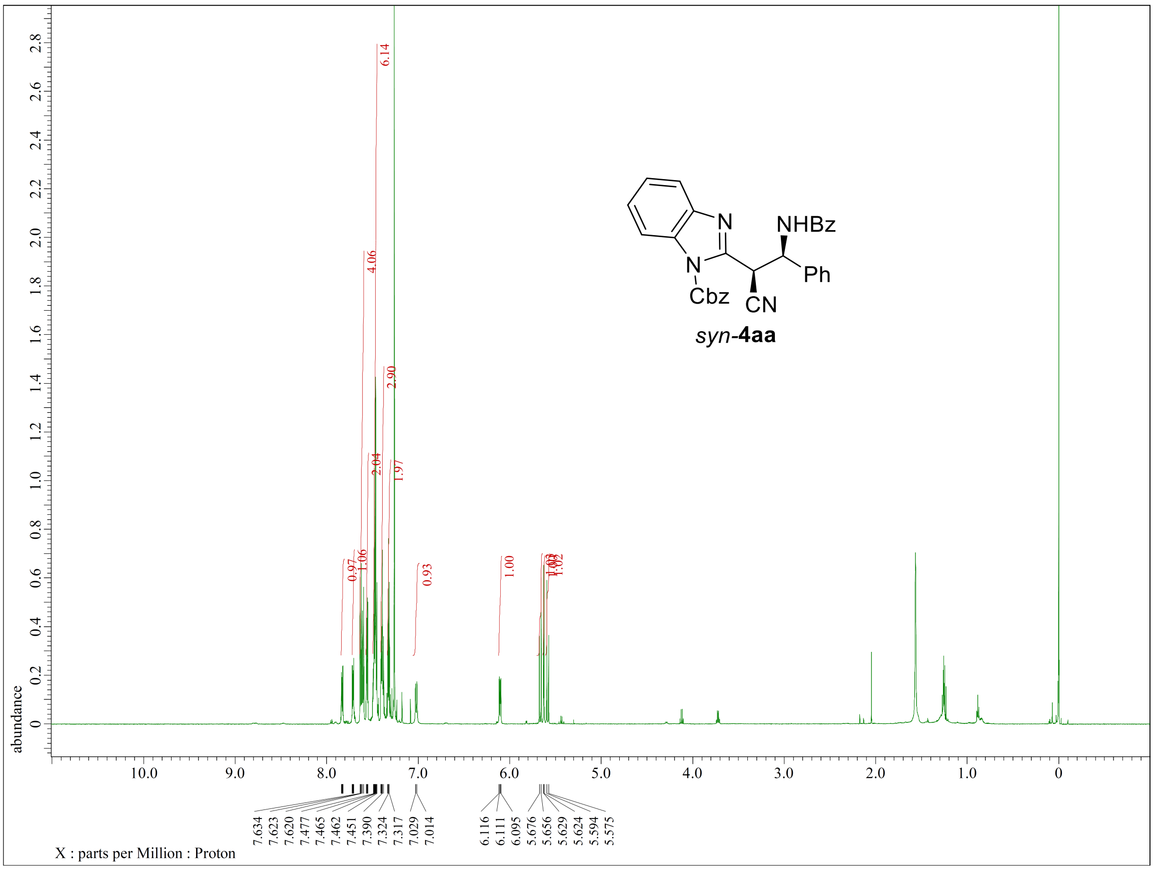

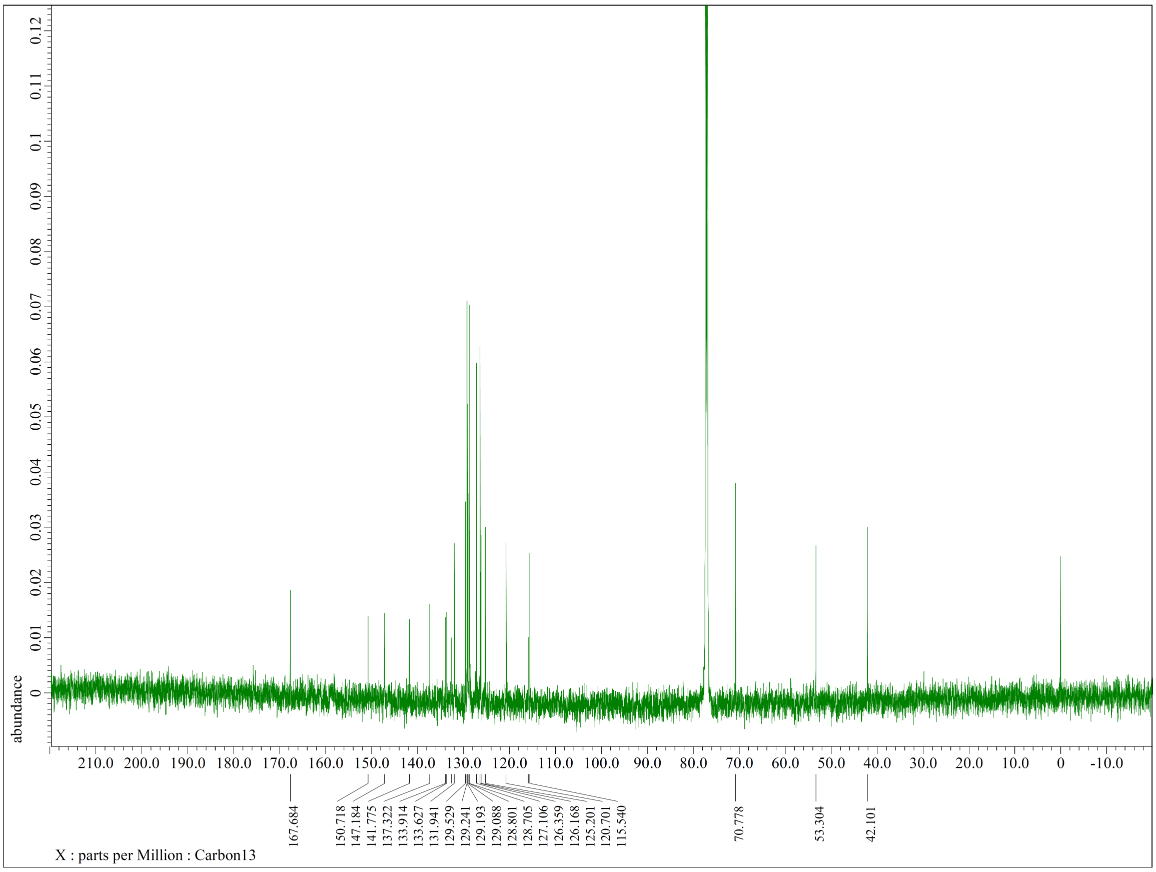

^{1}H NMR (600 MHz, $CDCl_3$) and ^{13}C NMR (151 MHz, $CDCl_3$) spectra of *anti*-**4ba**

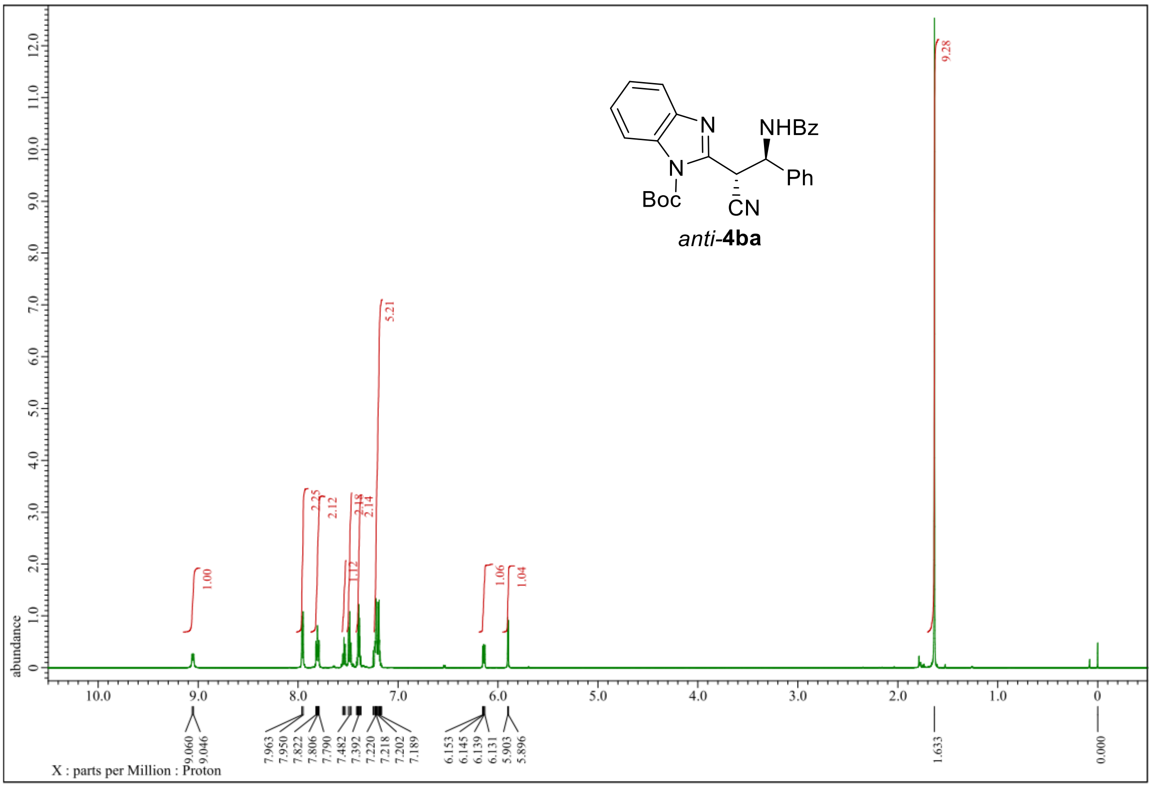

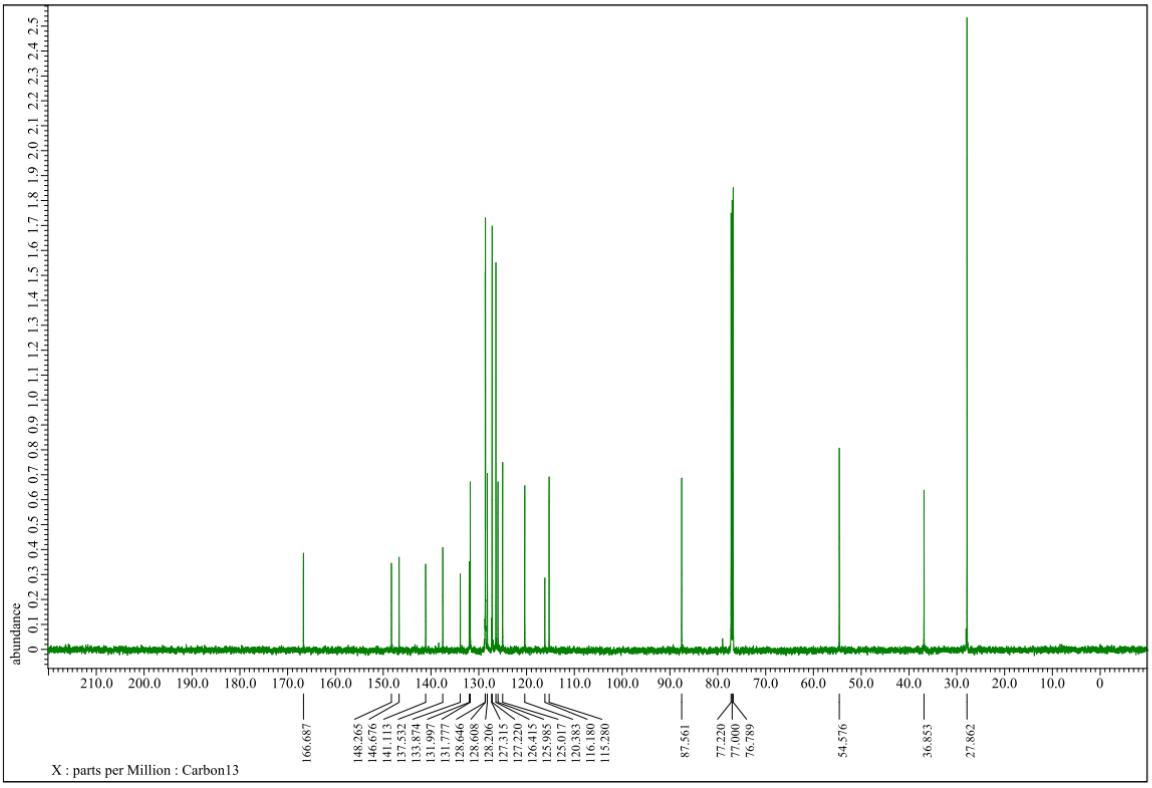

^{1}H NMR (600 MHz, $CDCl_3$) and ^{13}C NMR (151 MHz, $CDCl_3$) spectra of *anti*-**4ab**

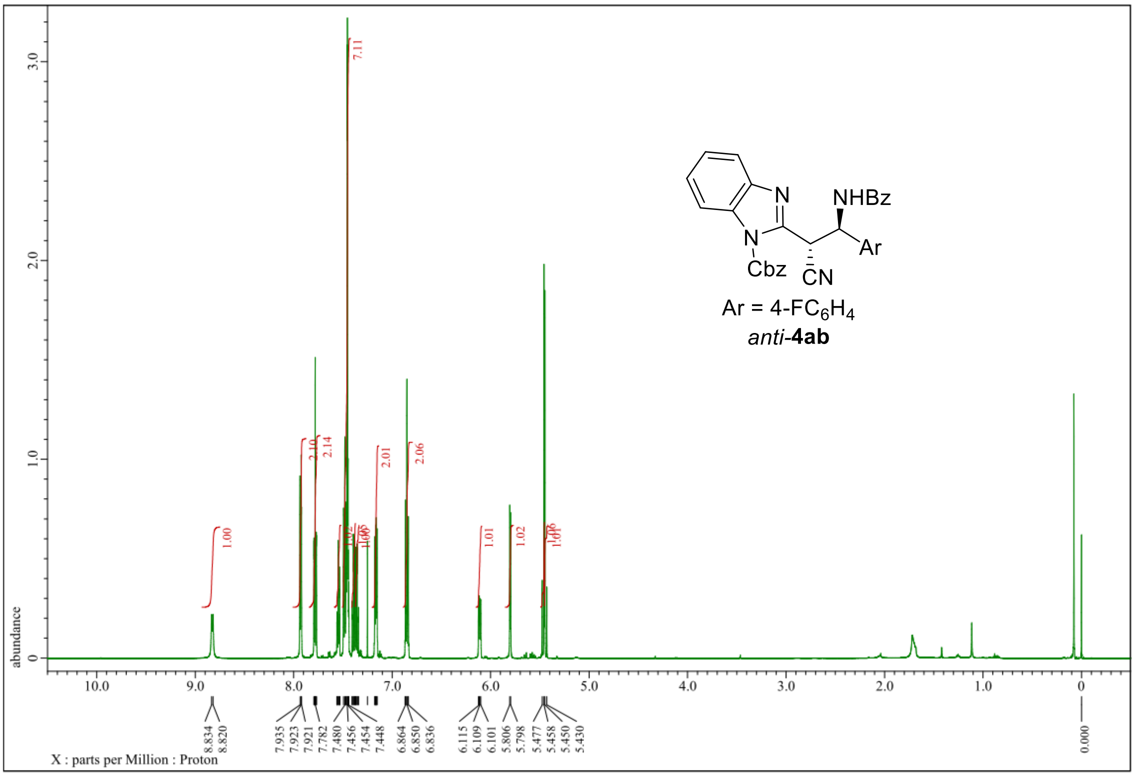

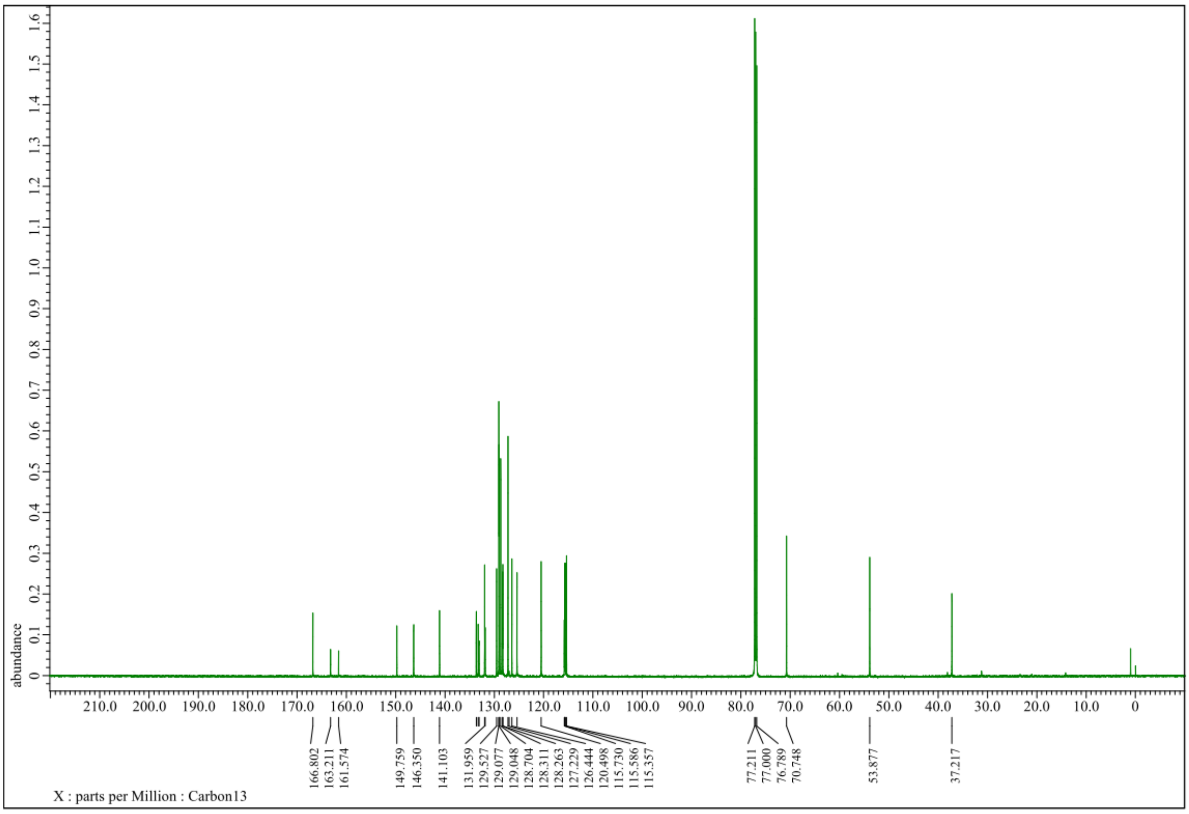

^{19}F NMR (565 MHz, $CDCl_3$) spectra of *anti*-**4ab**

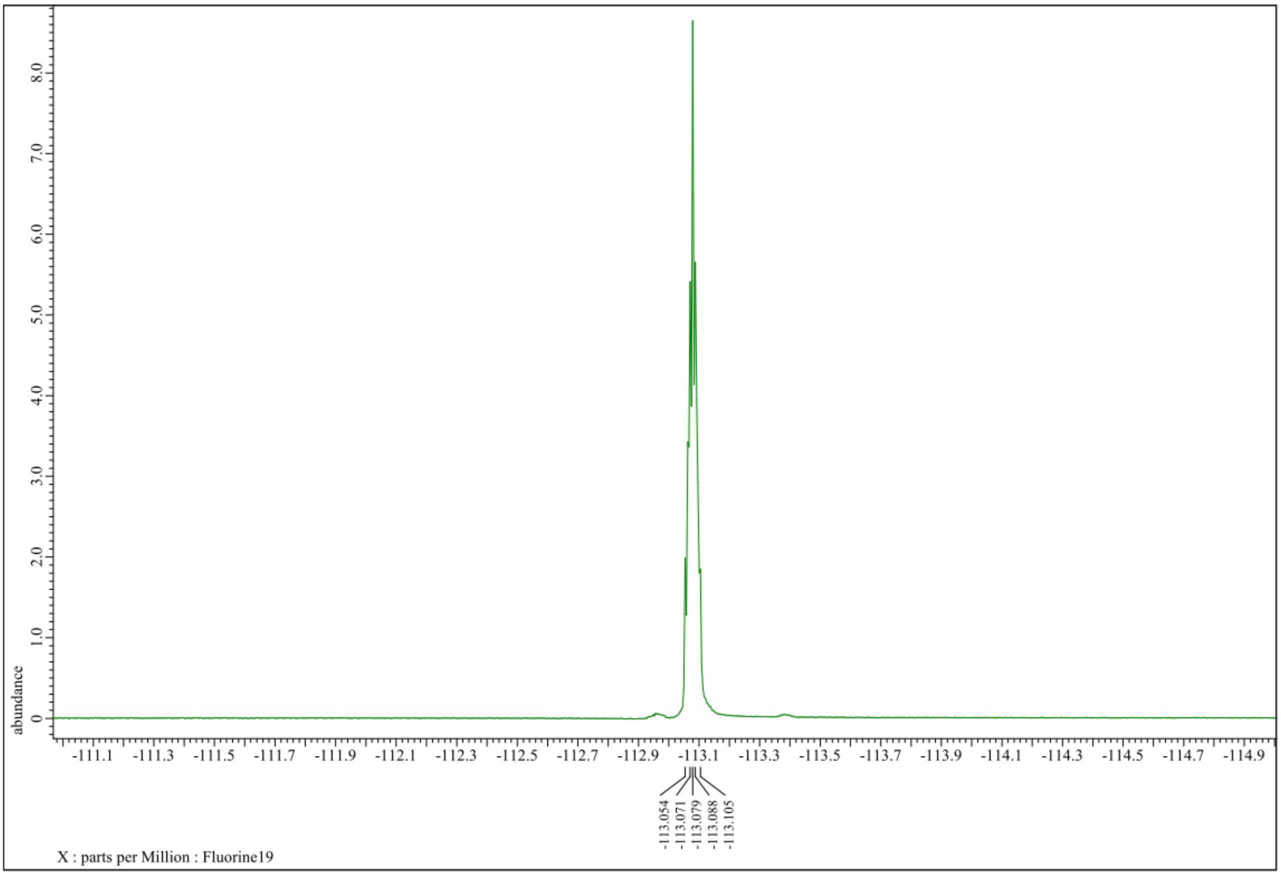

^{1}H NMR (600 MHz, $CDCl_3$) and ^{13}C NMR (151 MHz, $CDCl_3$) spectra of *anti*-**4ac**

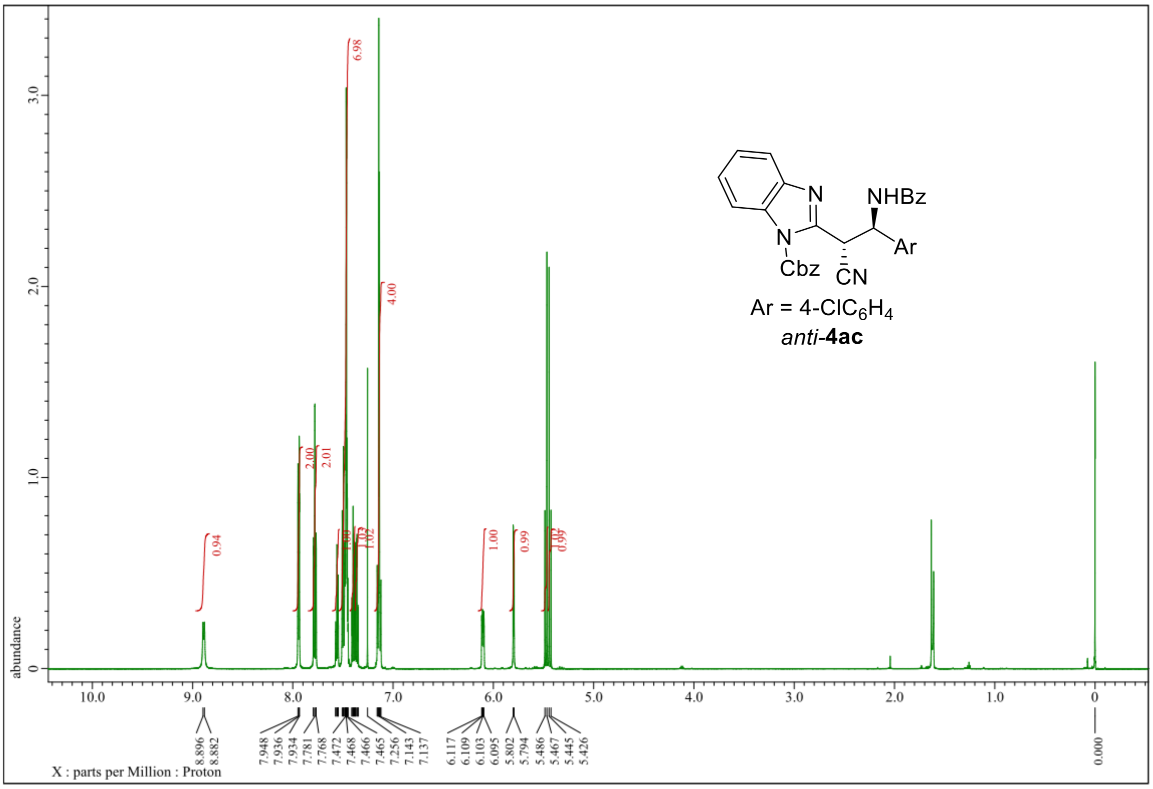

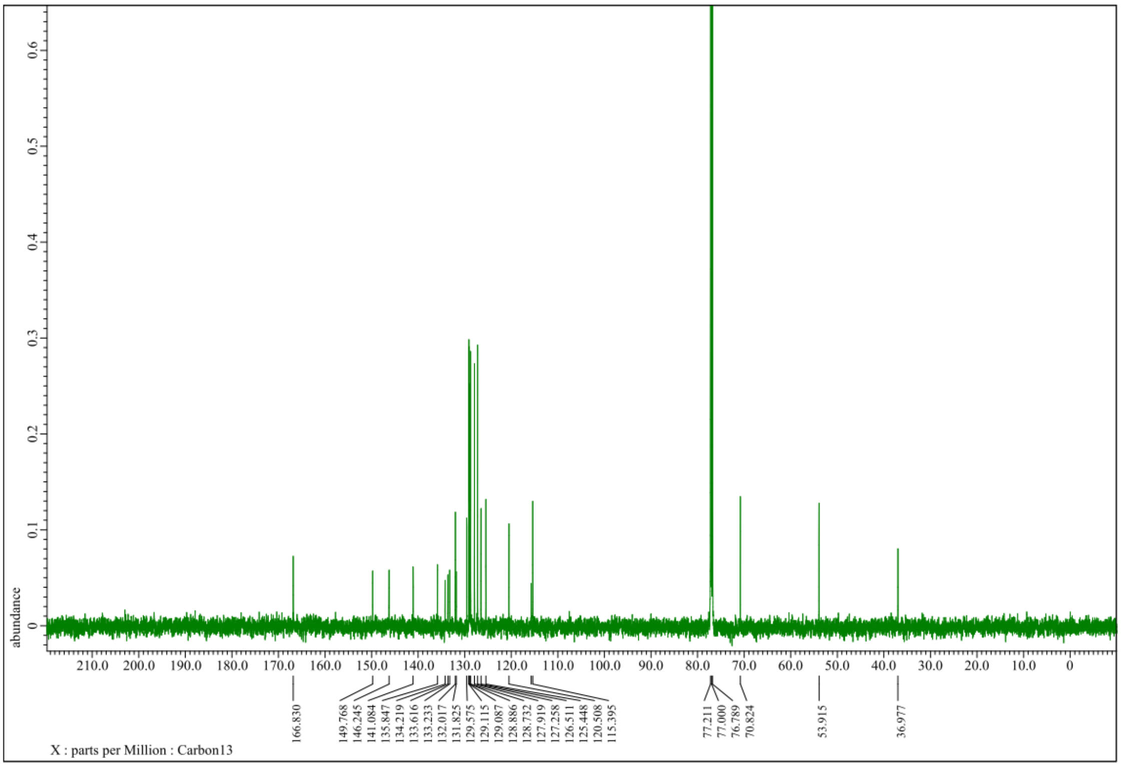

^{1}H NMR (600 MHz, $CDCl_3$) and ^{13}C NMR (151 MHz, $CDCl_3$) spectra of *syn*-**4ac**

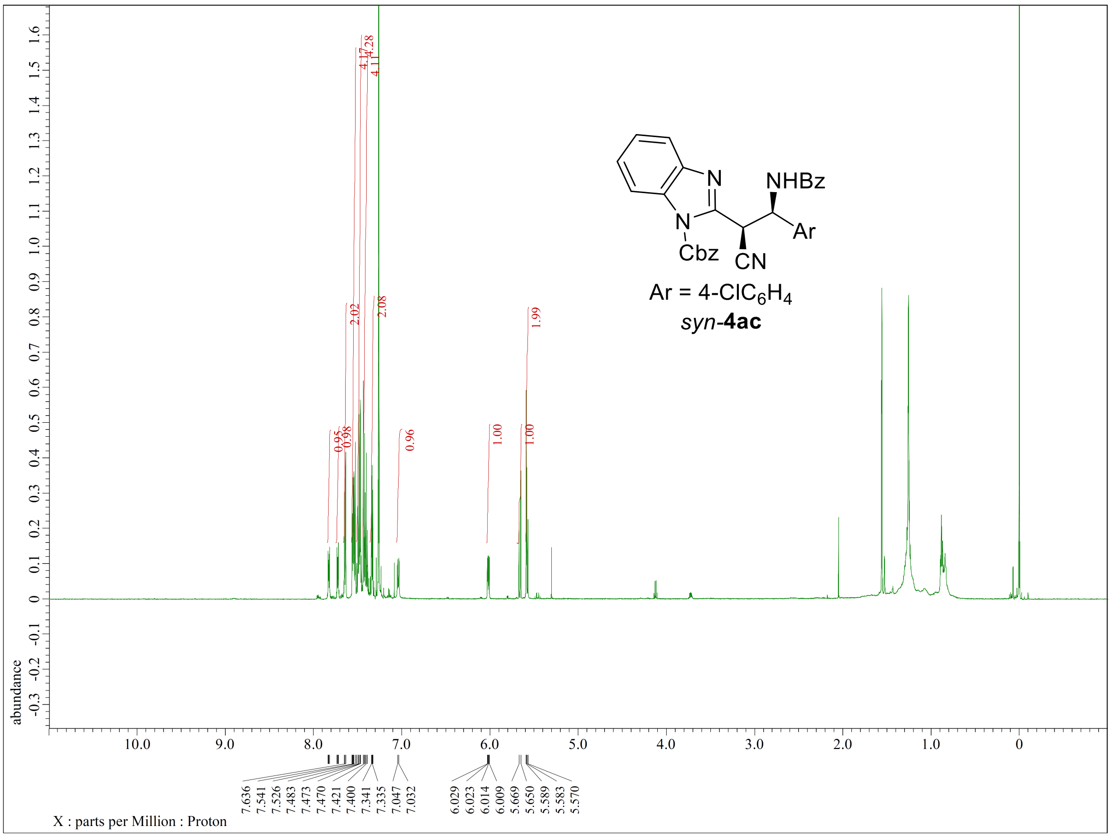

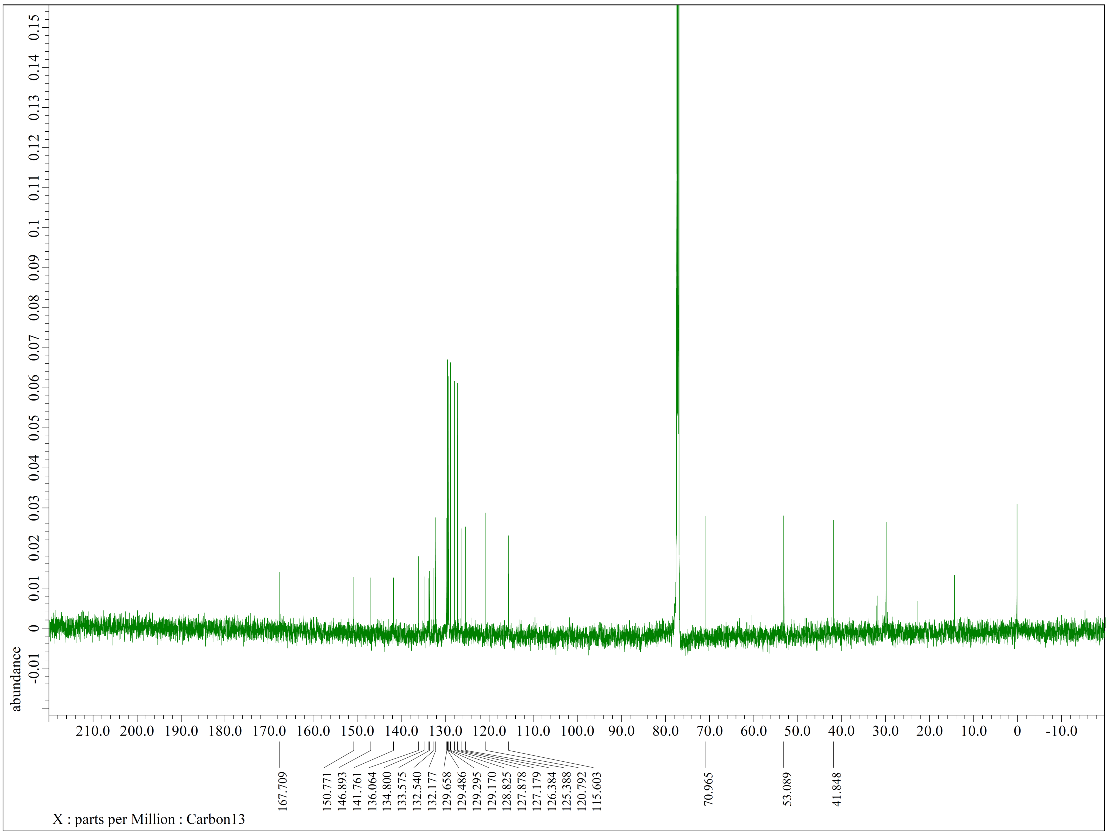

^{1}H NMR (600 MHz, $CDCl_3$) and ^{13}C NMR (151 MHz, $CDCl_3$) spectra of *anti*-**4ad**

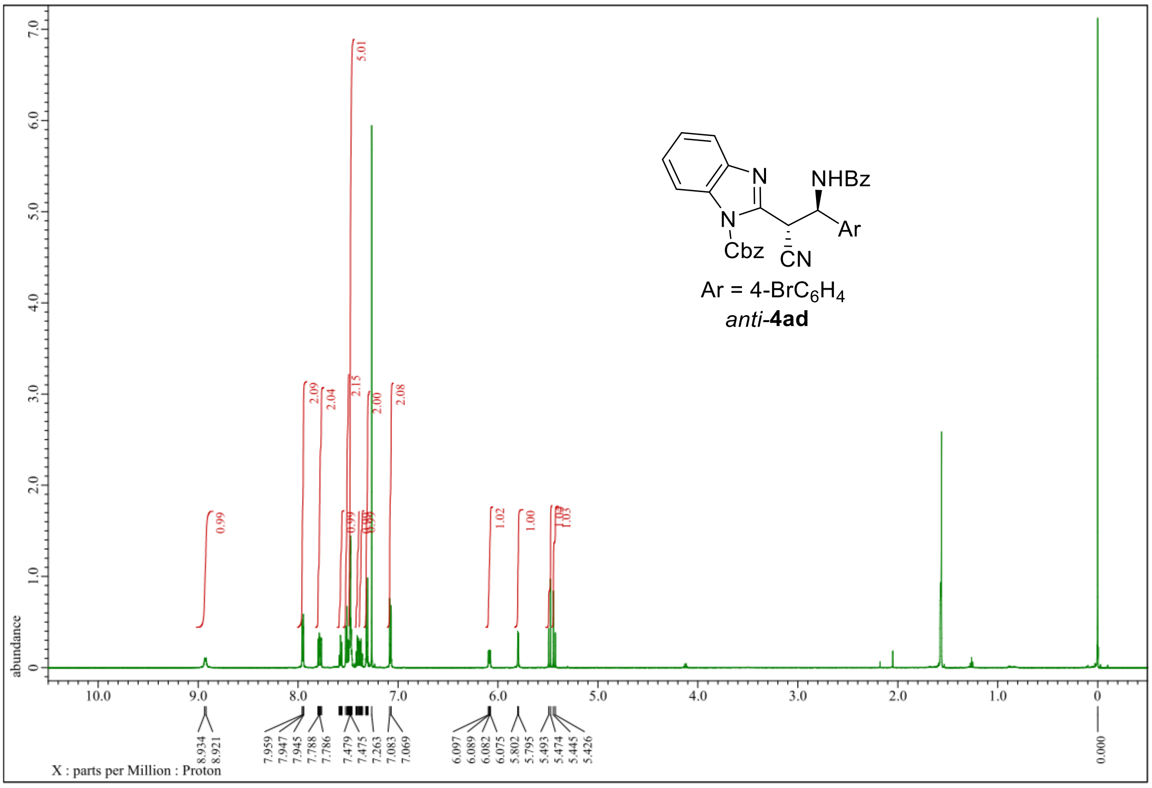

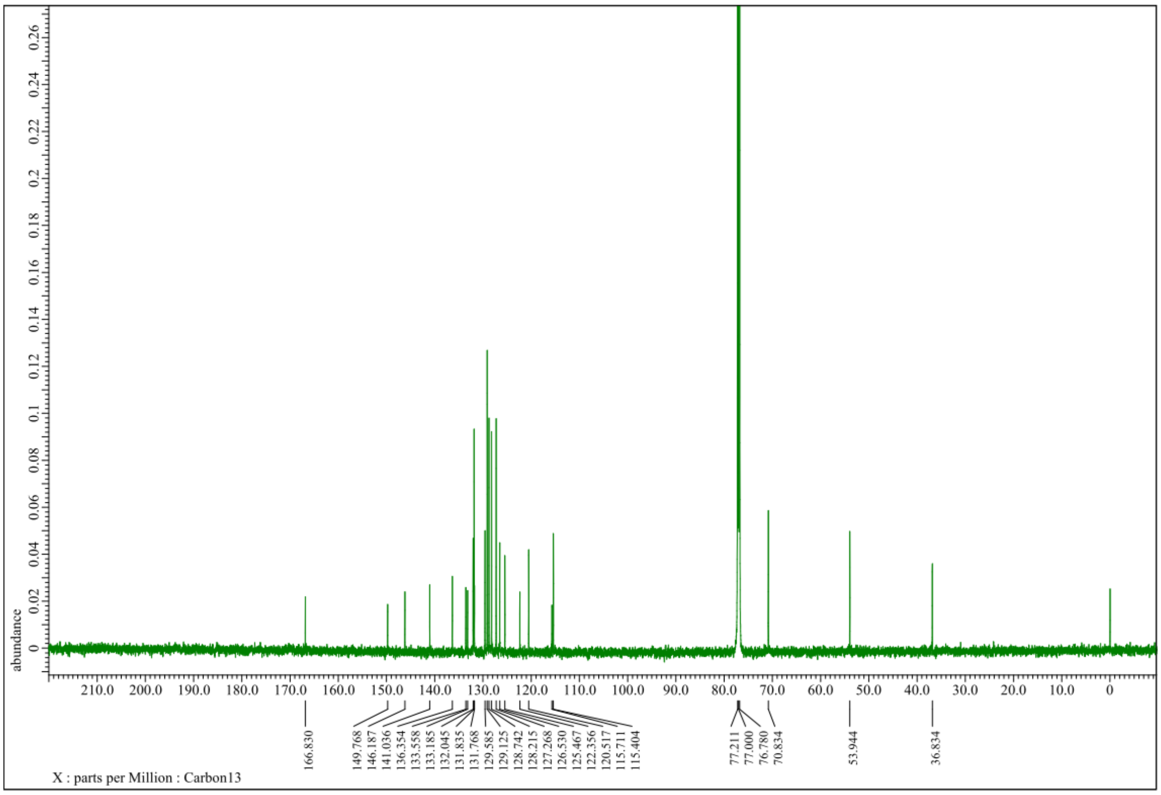

^{1}H NMR (600 MHz, $CDCl_3$) and ^{13}C NMR (151 MHz, $CDCl_3$) spectra of *anti*-**4ae**

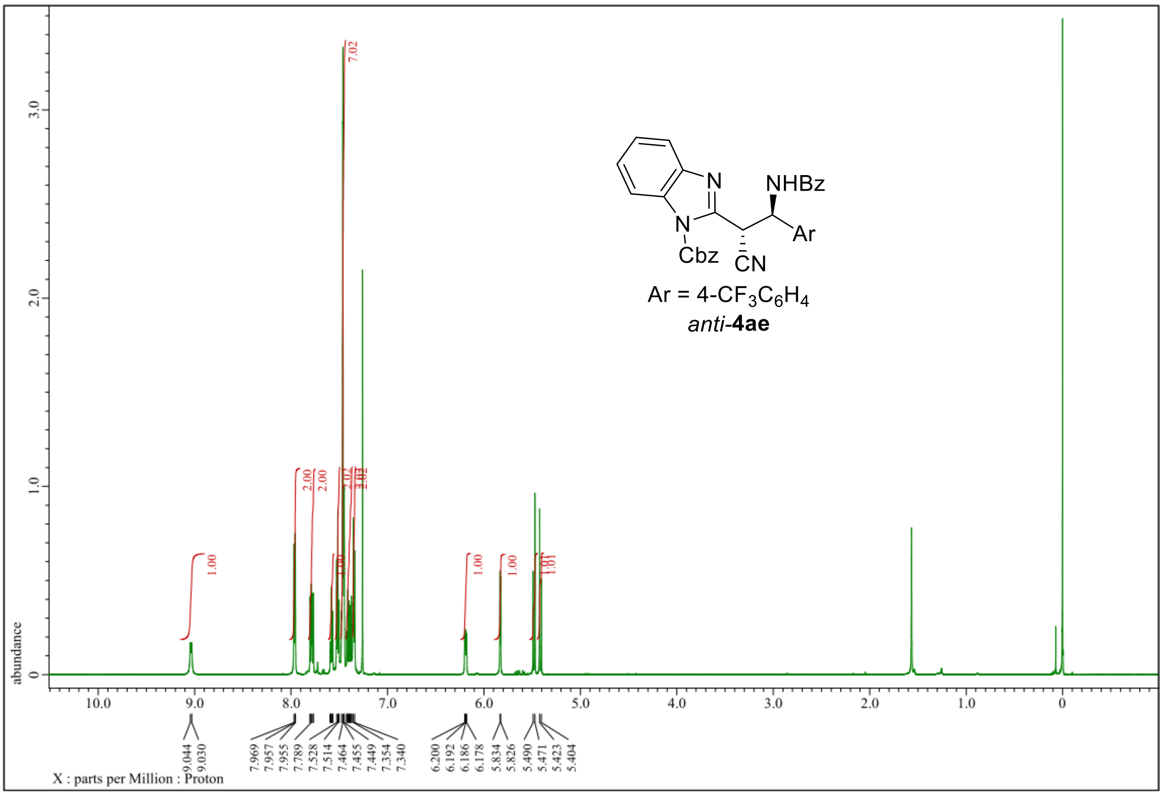

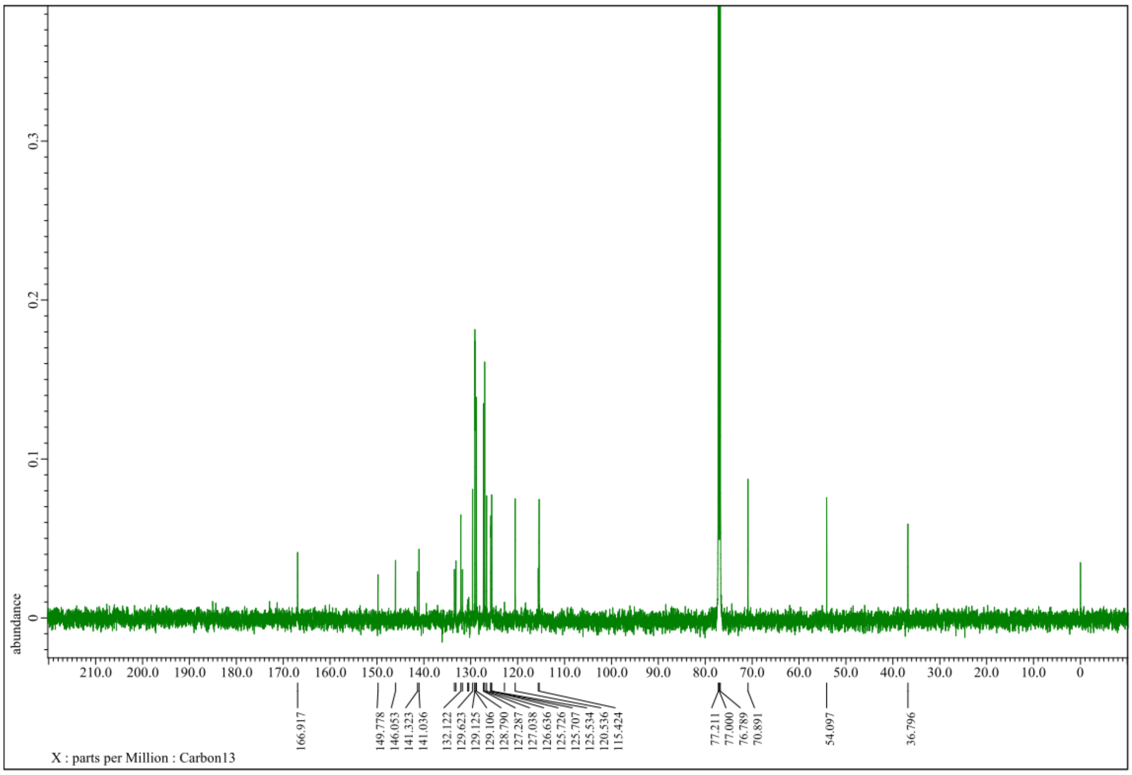

^{19}F NMR (565 MHz, $CDCl_3$) spectra of *anti*-**4ae**

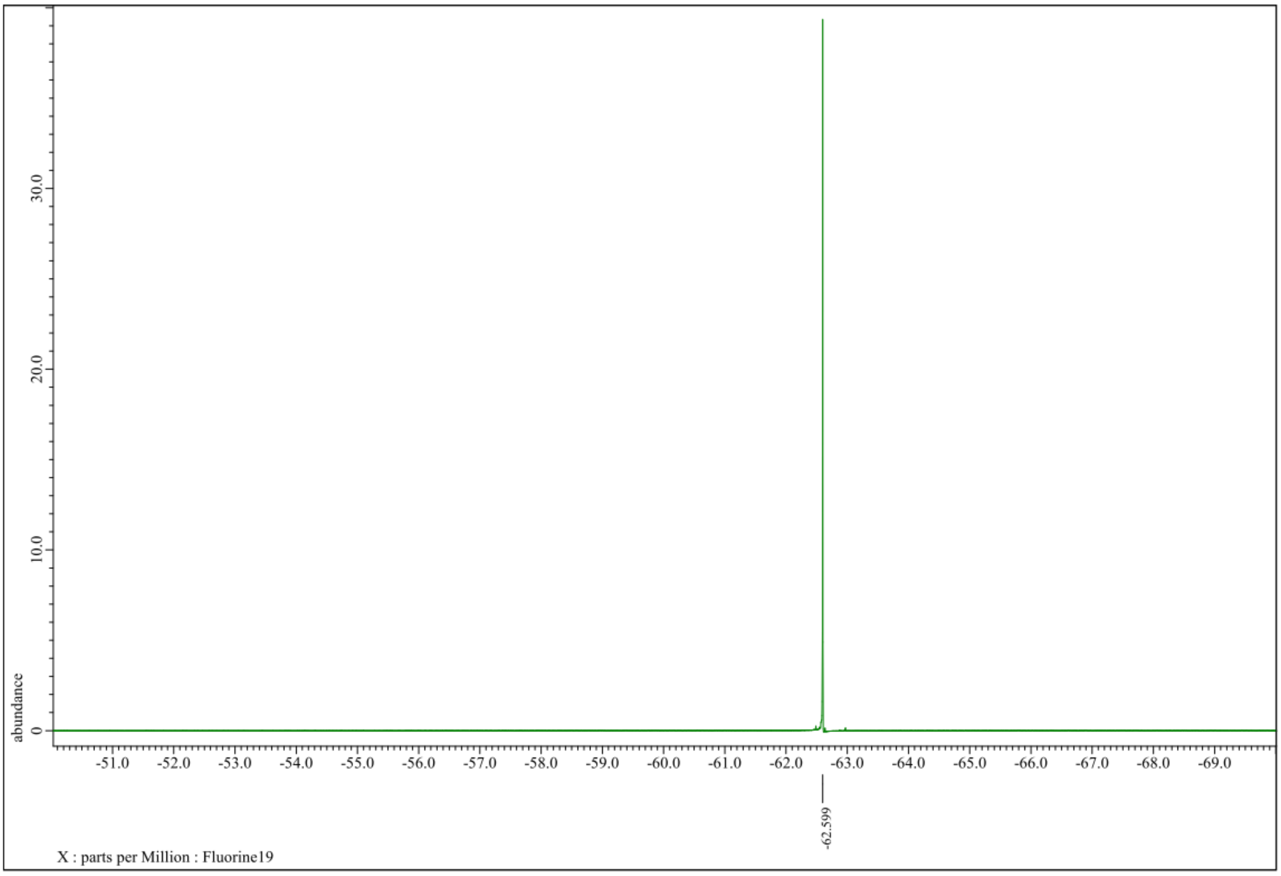

^{1}H NMR (600 MHz, $CDCl_3$) and ^{13}C NMR (151 MHz, $CDCl_3$) spectra of *anti*-**4af**

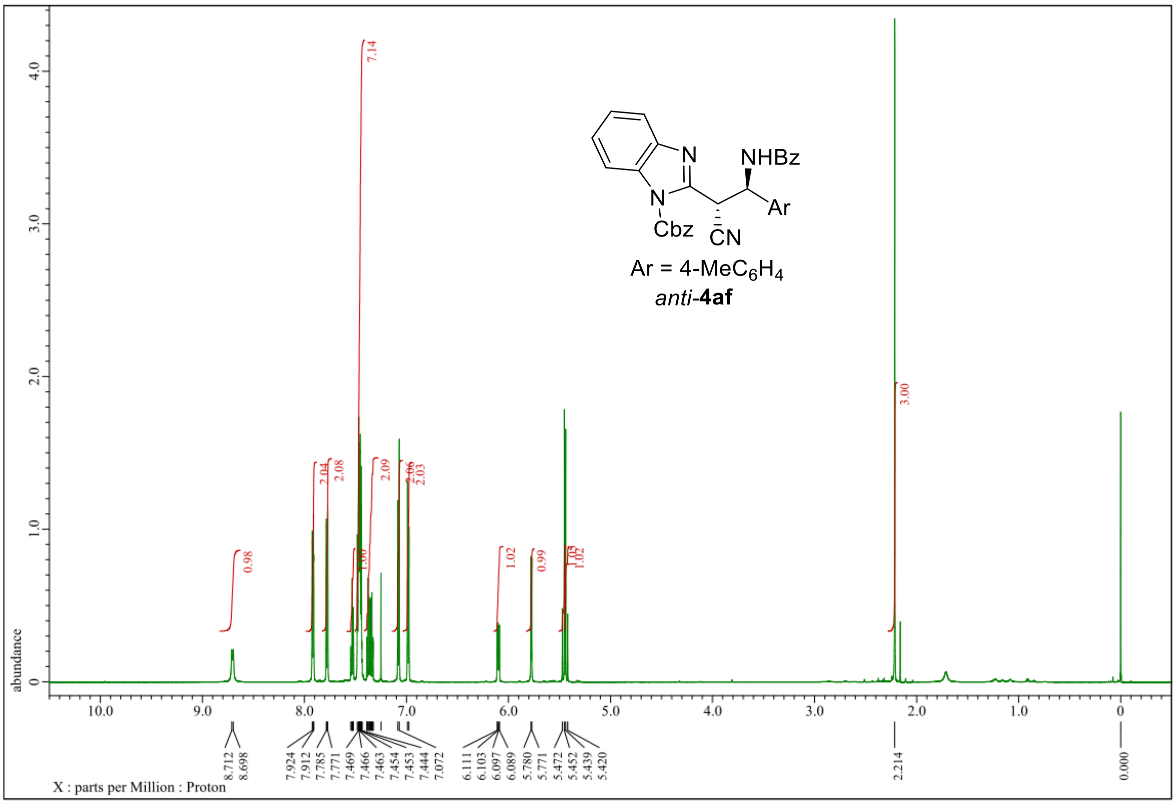

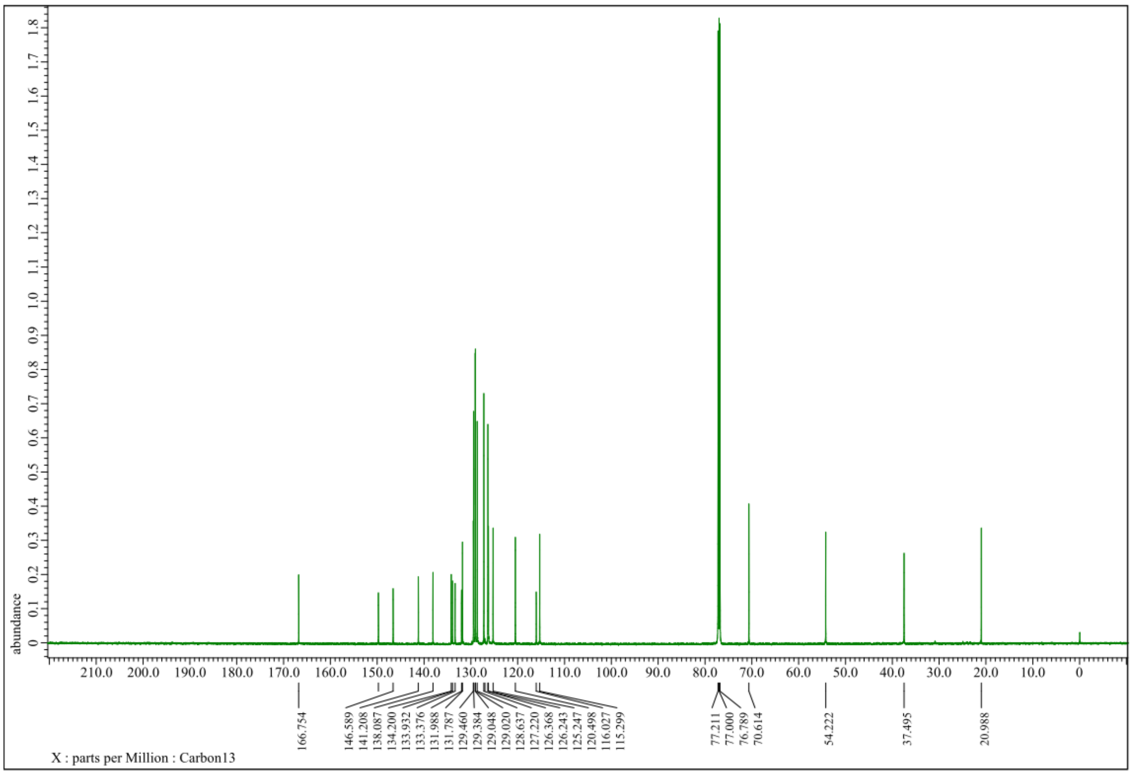

^{1}H NMR (600 MHz, $CDCl_3$) and ^{13}C NMR (151 MHz, $CDCl_3$) spectra of *anti*-**4ag**

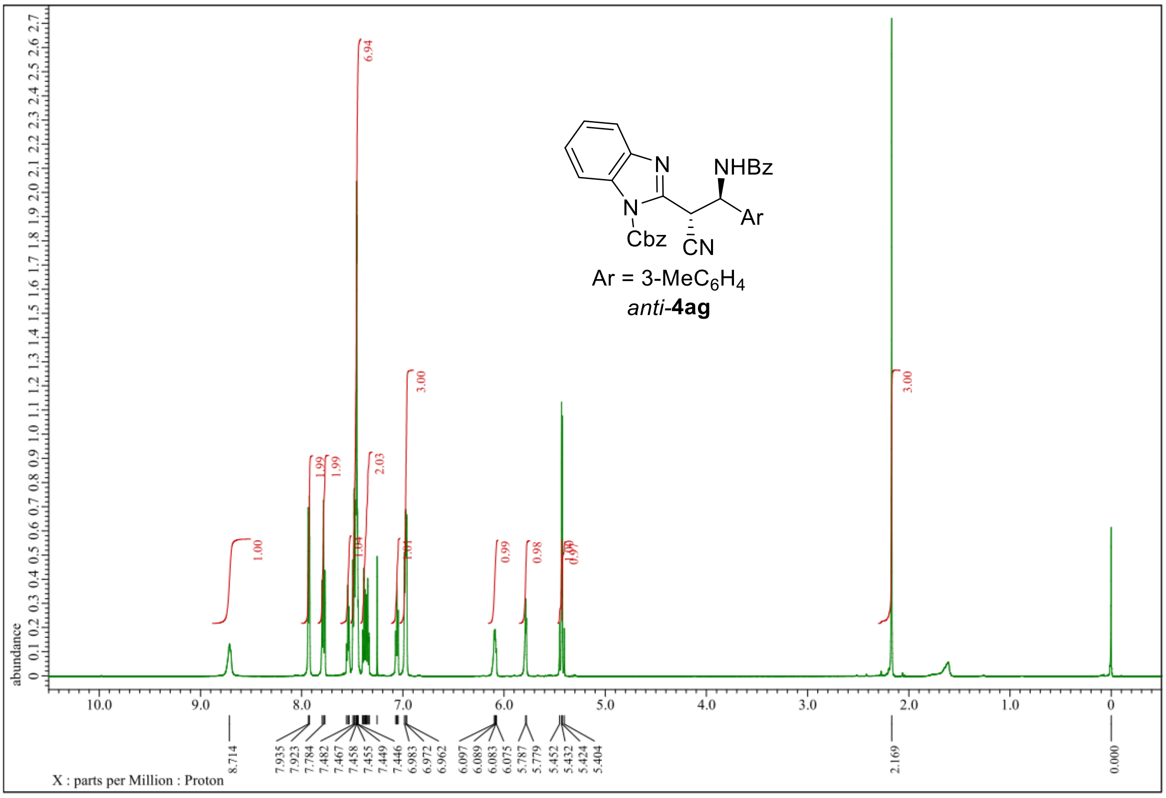

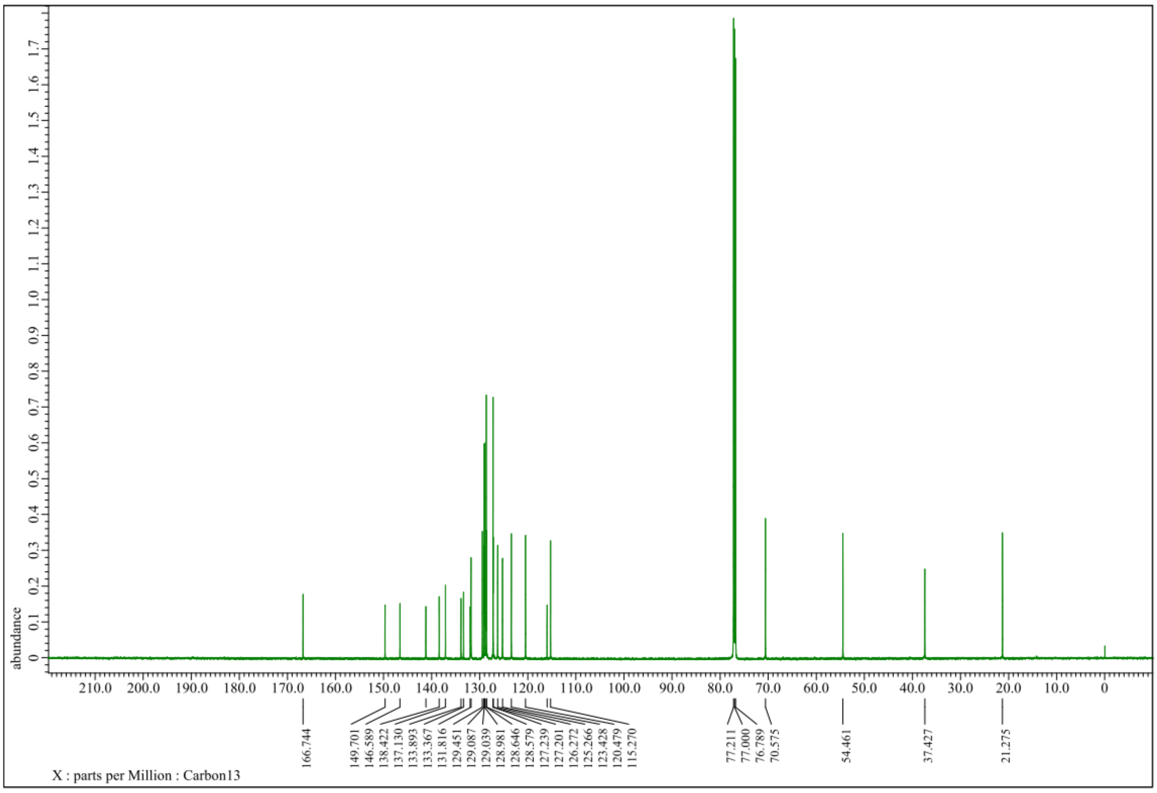

^{1}H NMR (400 MHz, $CDCl_3$) and ^{13}C NMR (100 MHz, $CDCl_3$) spectra of *anti*-**4ah**

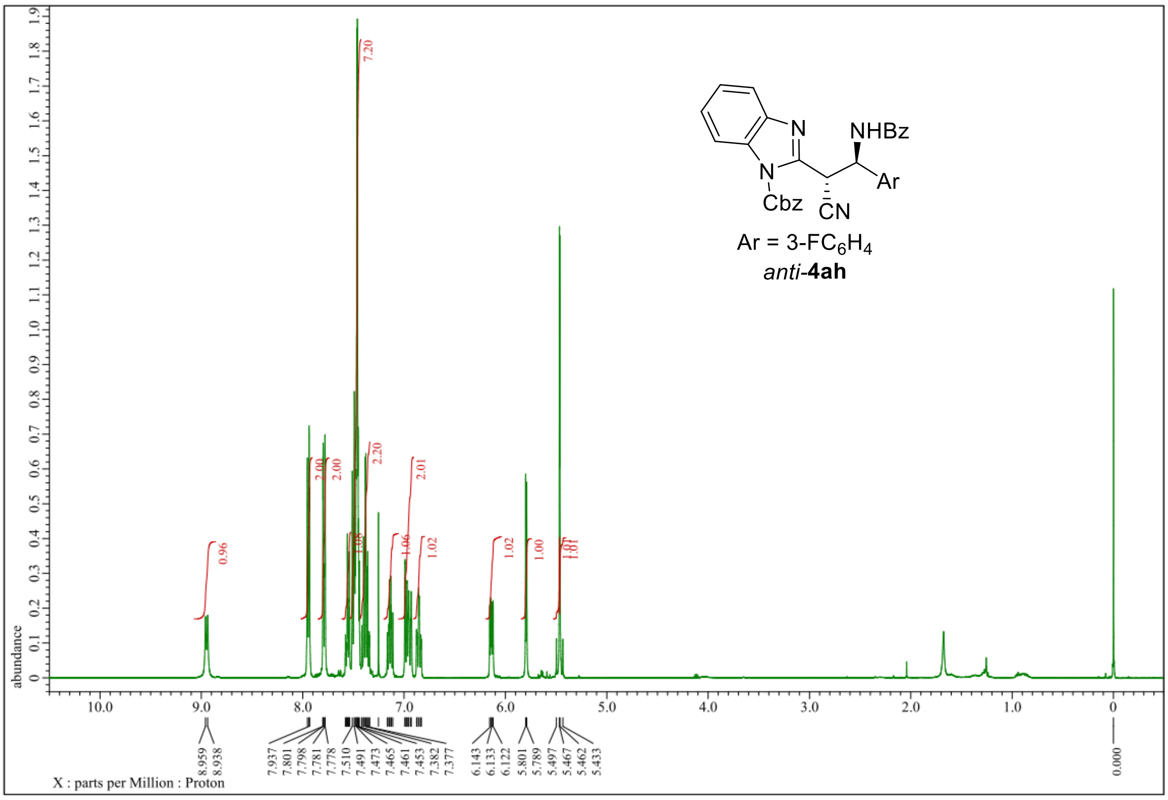

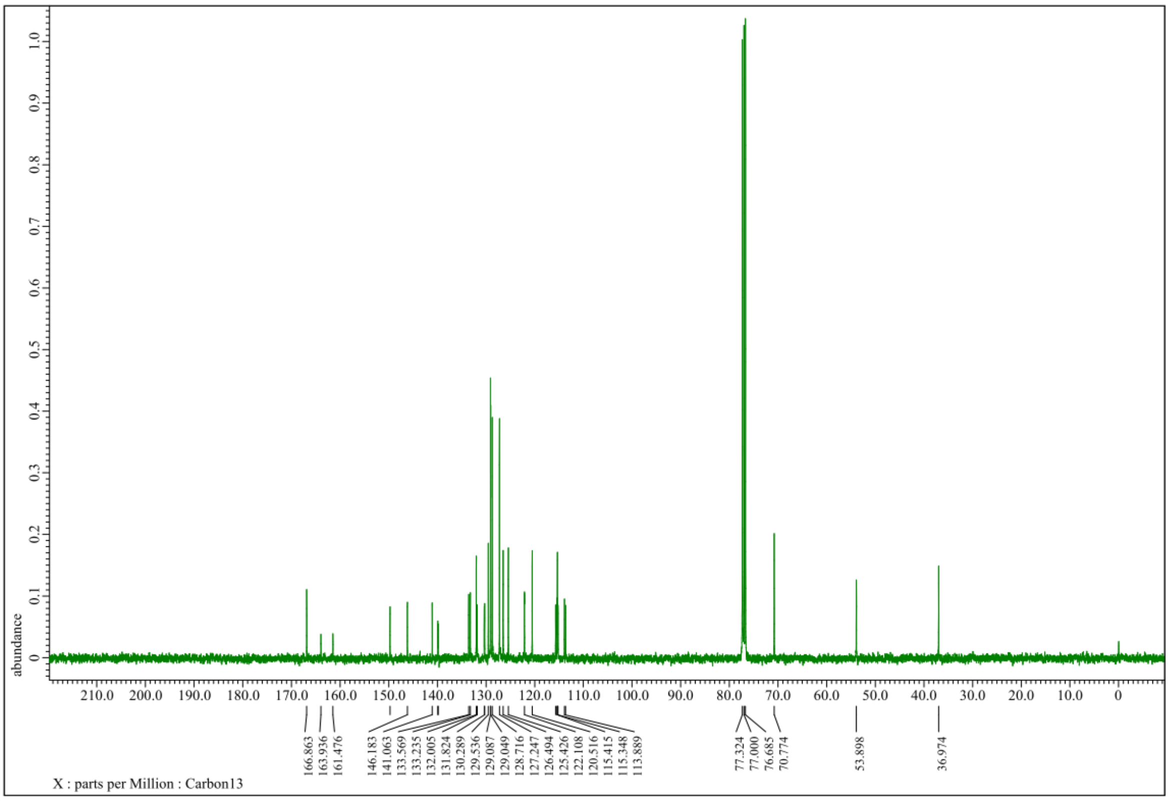

^{19}F NMR (377 MHz, $CDCl_3$) spectra of *anti*-**4ah**

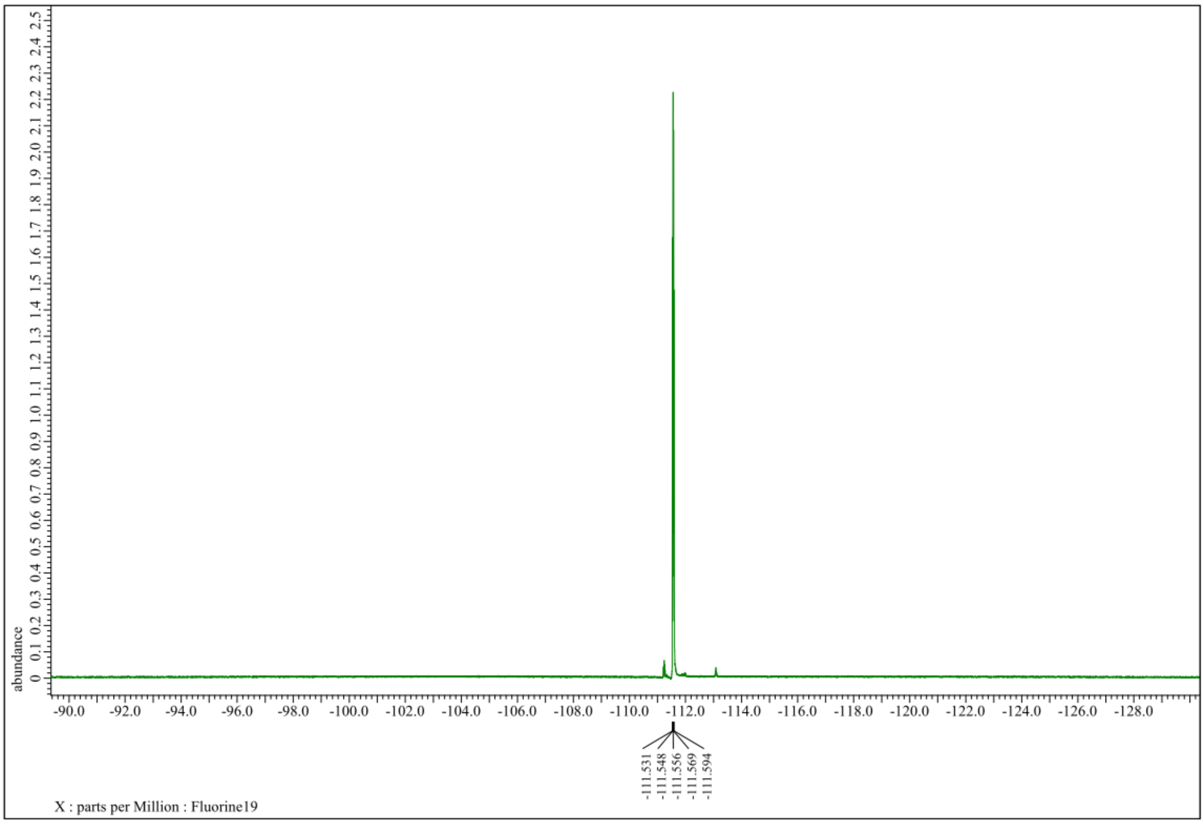

^{1}H NMR (400 MHz, $CDCl_3$) and ^{13}C NMR (100 MHz, $CDCl_3$) spectra of *anti*-**4ai**

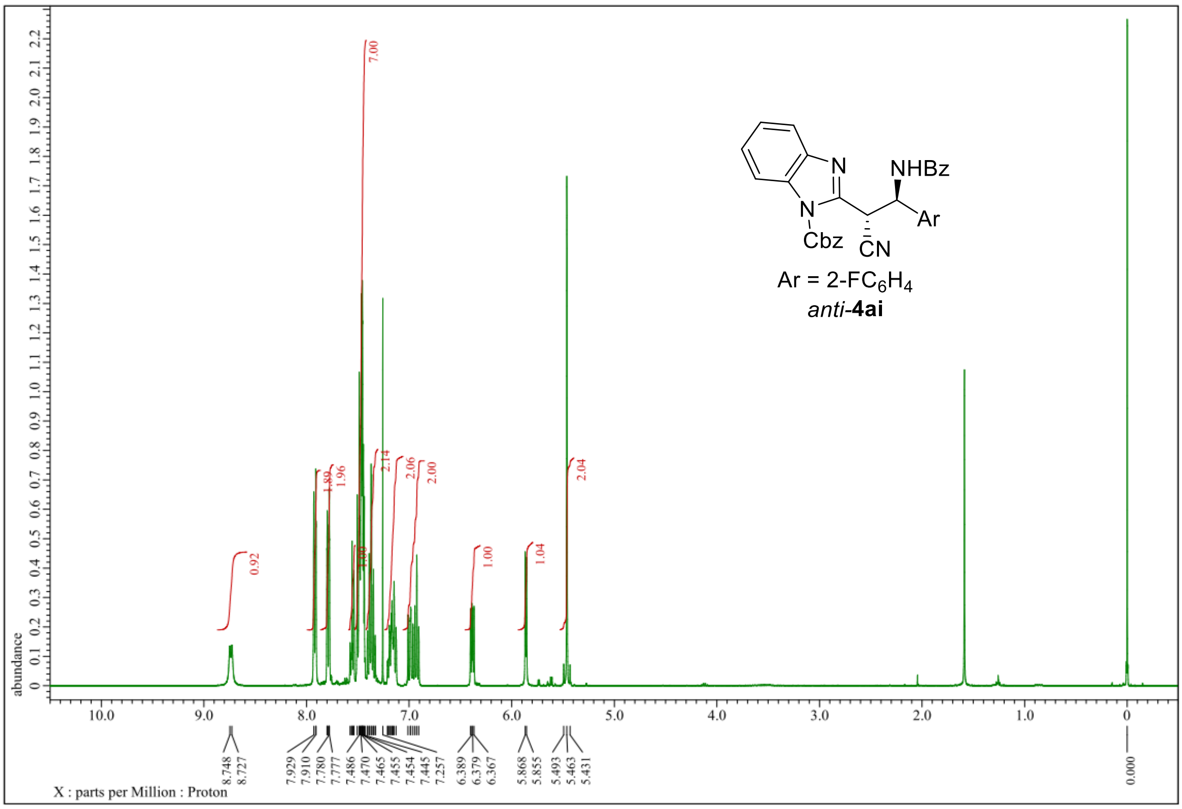

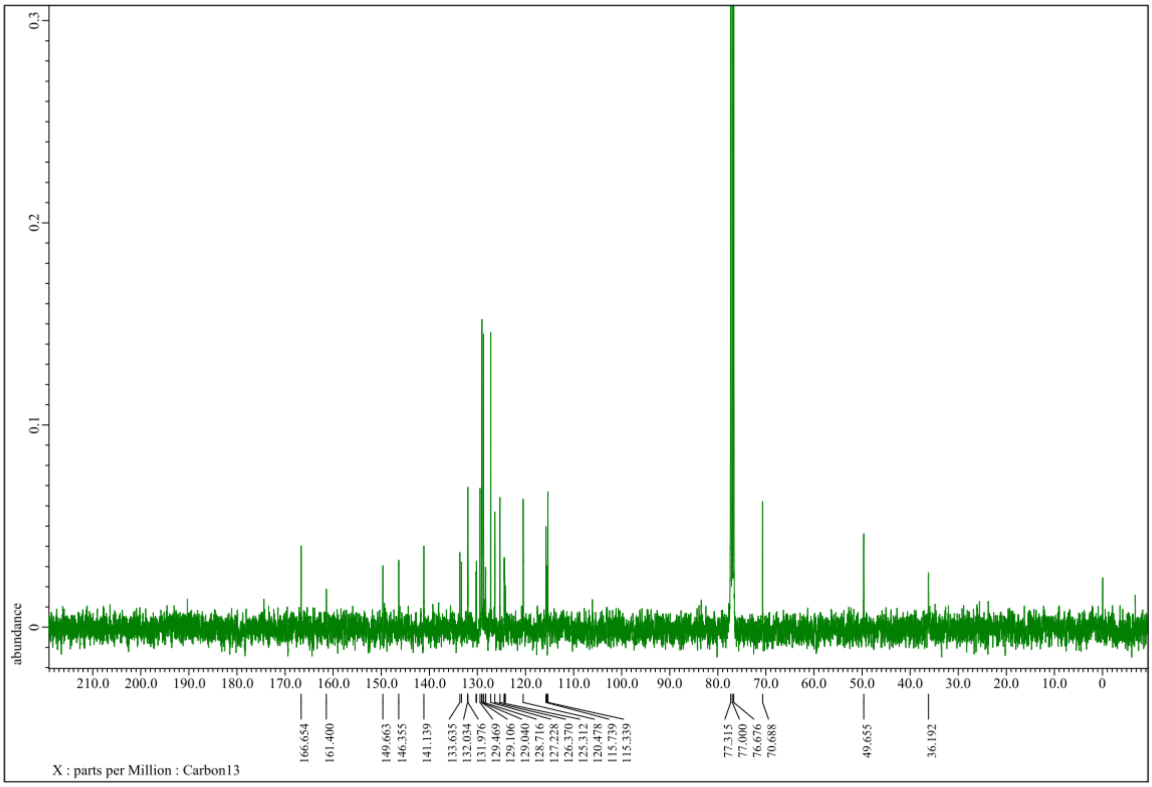

^{19}F NMR (377 MHz, $CDCl_3$) spectra of *anti*-**4ai**

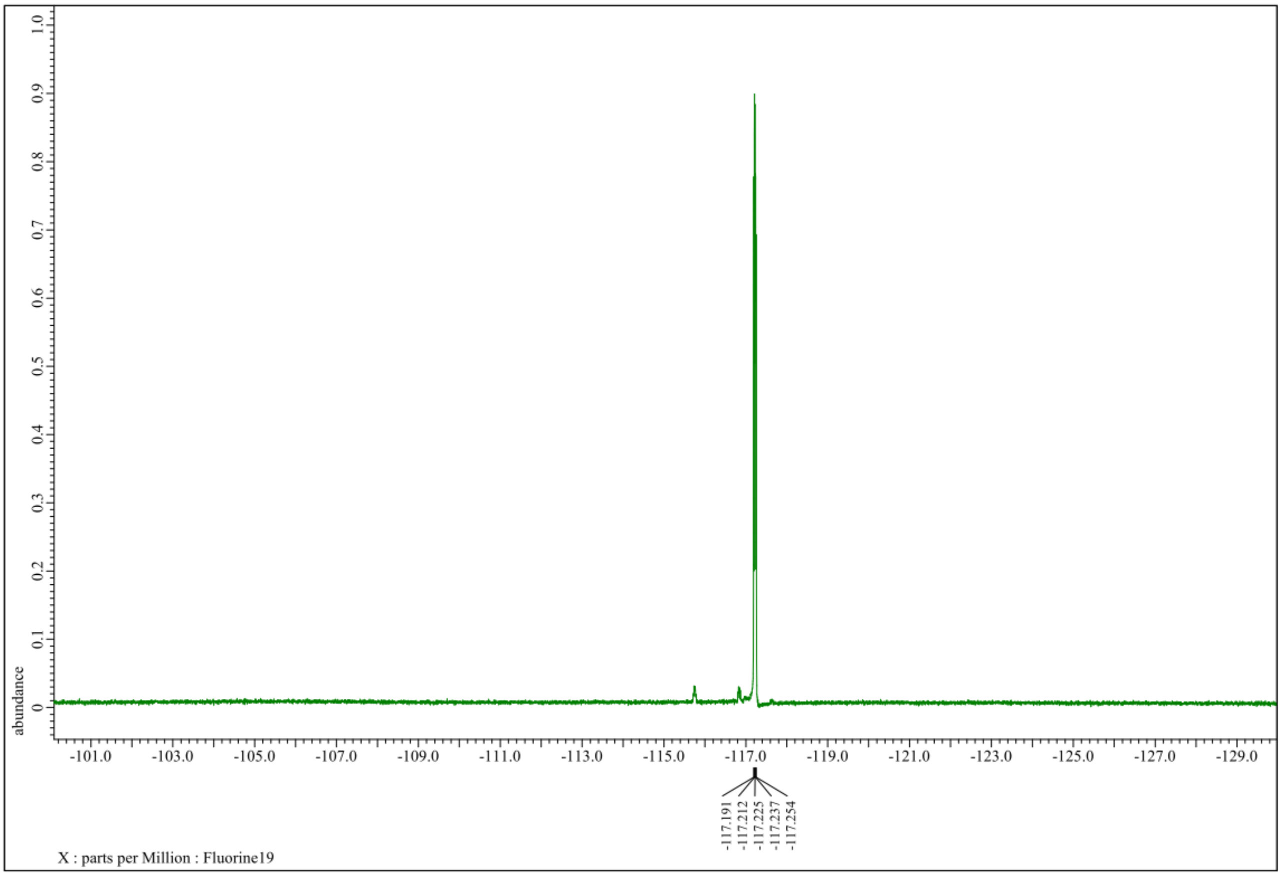

^{1}H NMR (600 MHz, $CDCl_3$) and ^{13}C NMR (151 MHz, $CDCl_3$) spectra of *anti*-**4aj**

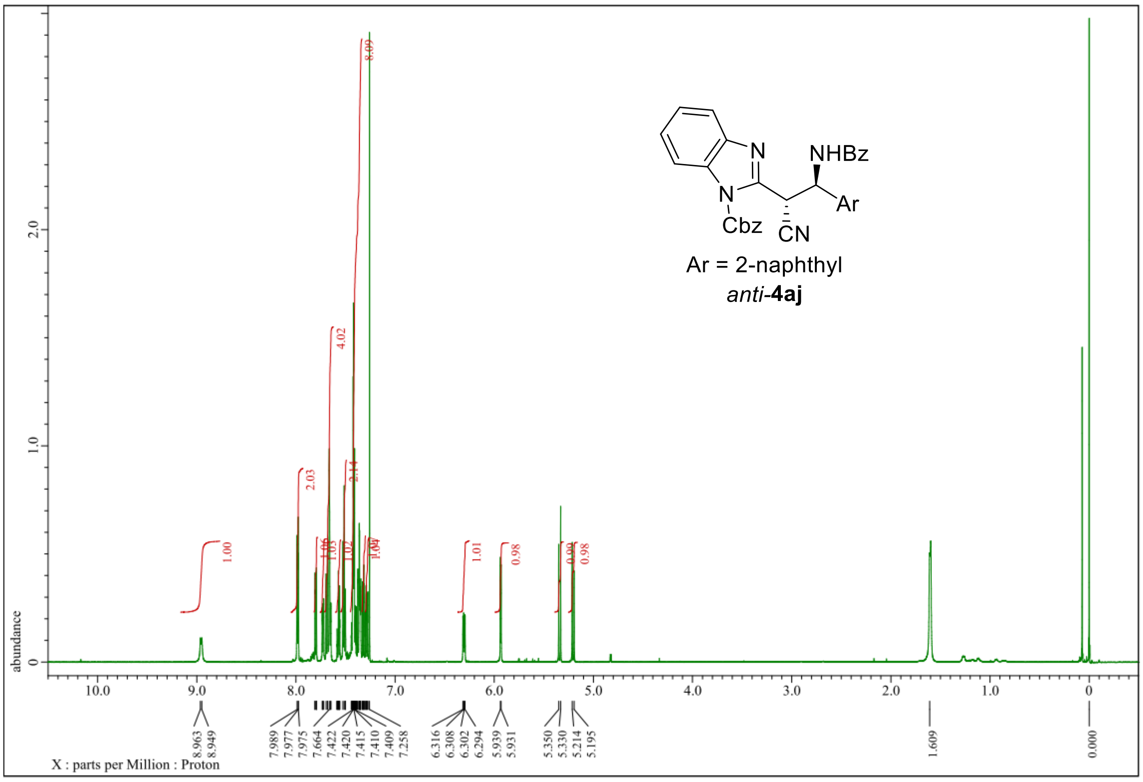

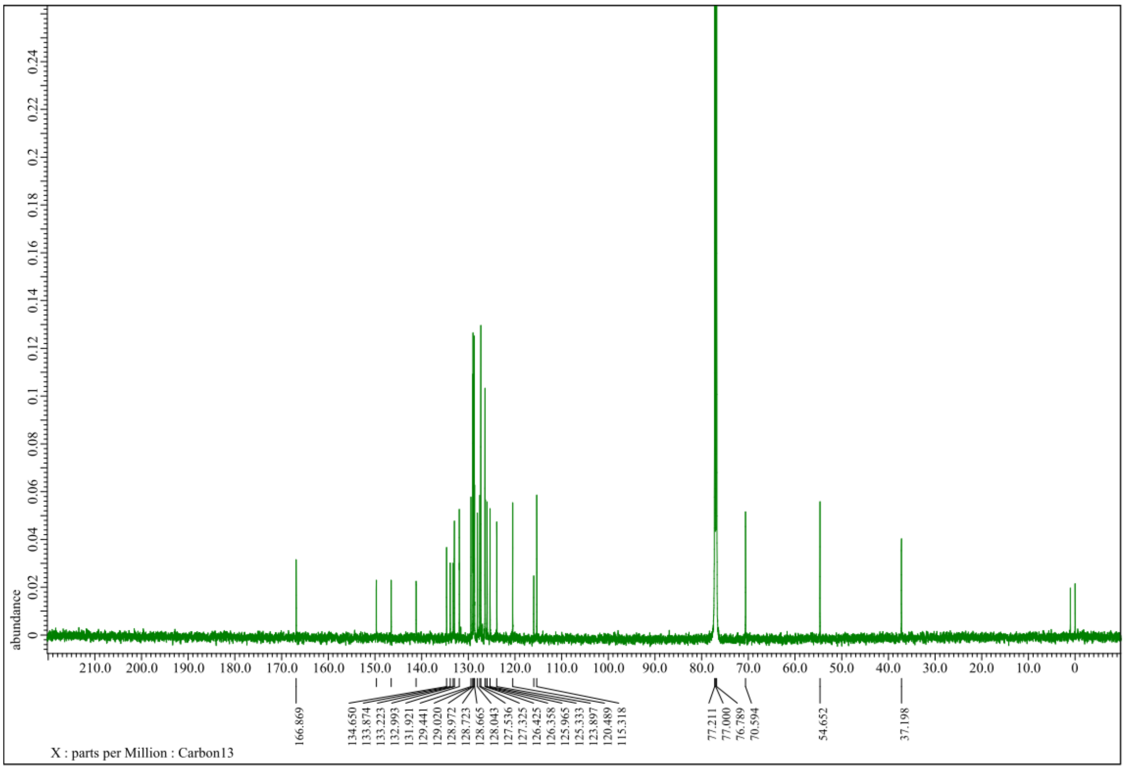

^{1}H NMR (600 MHz, $CDCl_3$) and ^{13}C NMR (151 MHz, $CDCl_3$) spectra of *anti*-**4ak**

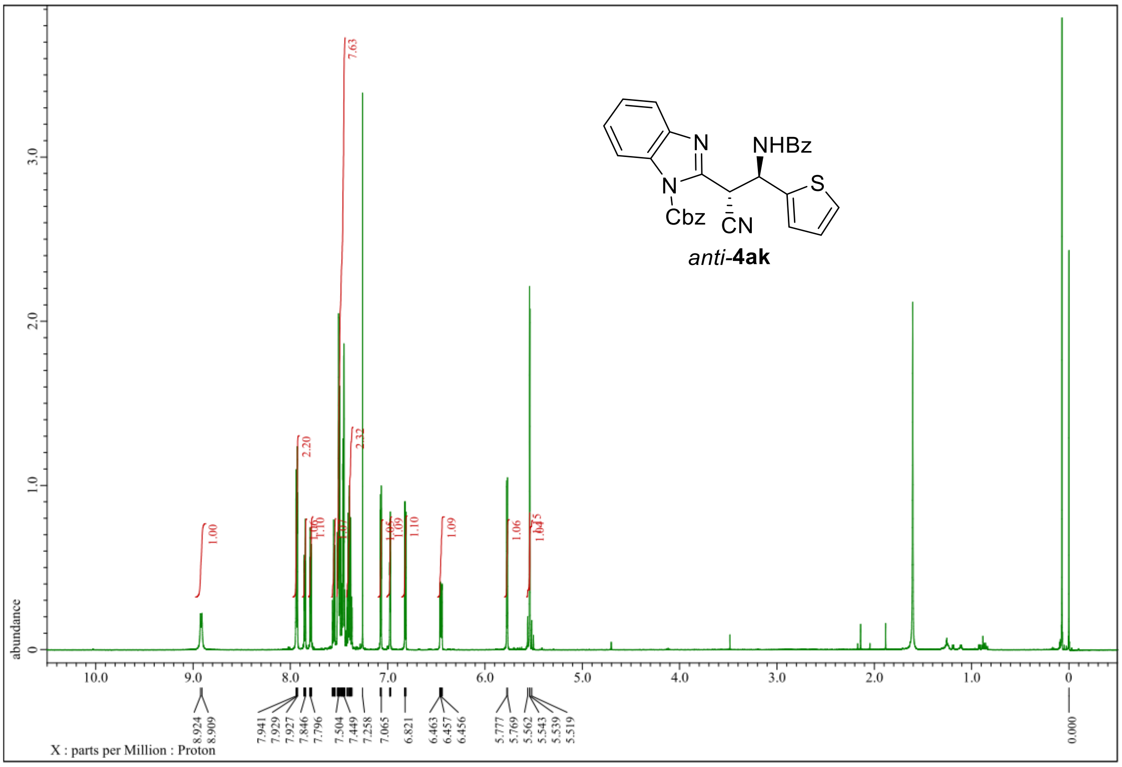

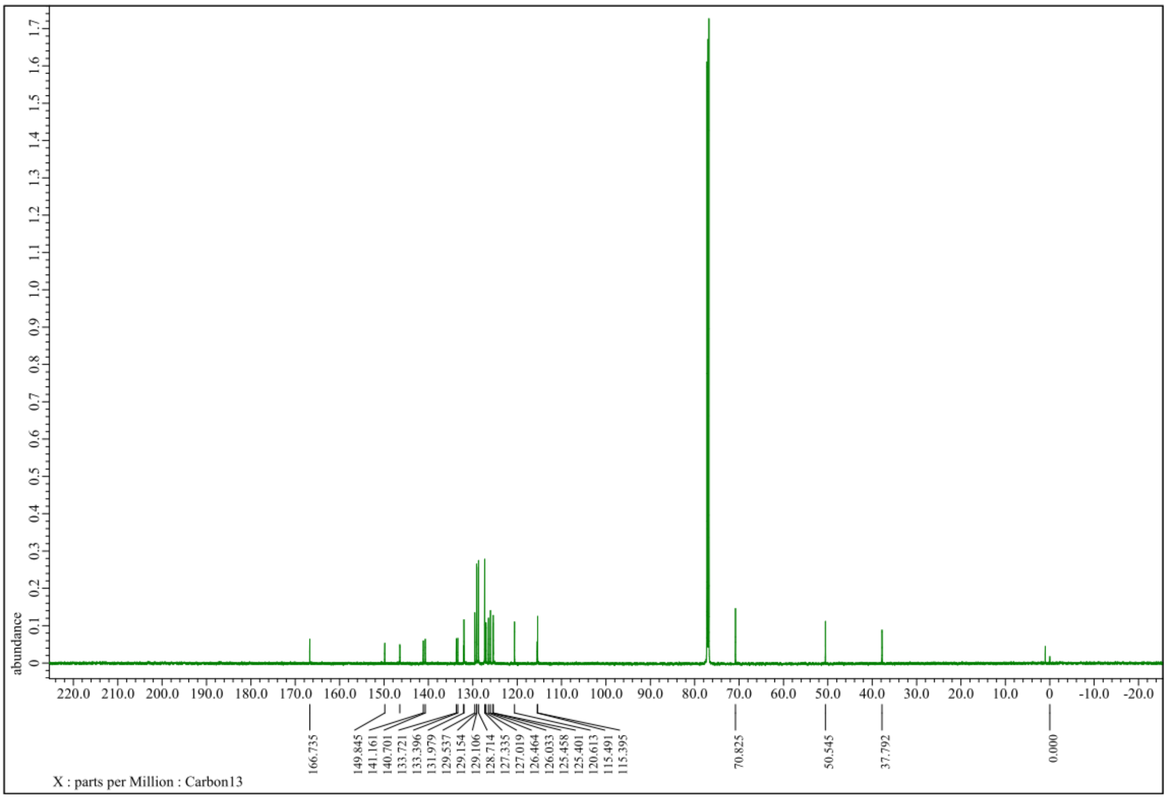

^{1}H NMR (600 MHz, $CDCl_3$) and ^{13}C NMR (151 MHz, $CDCl_3$) spectra of *anti*-**4al**

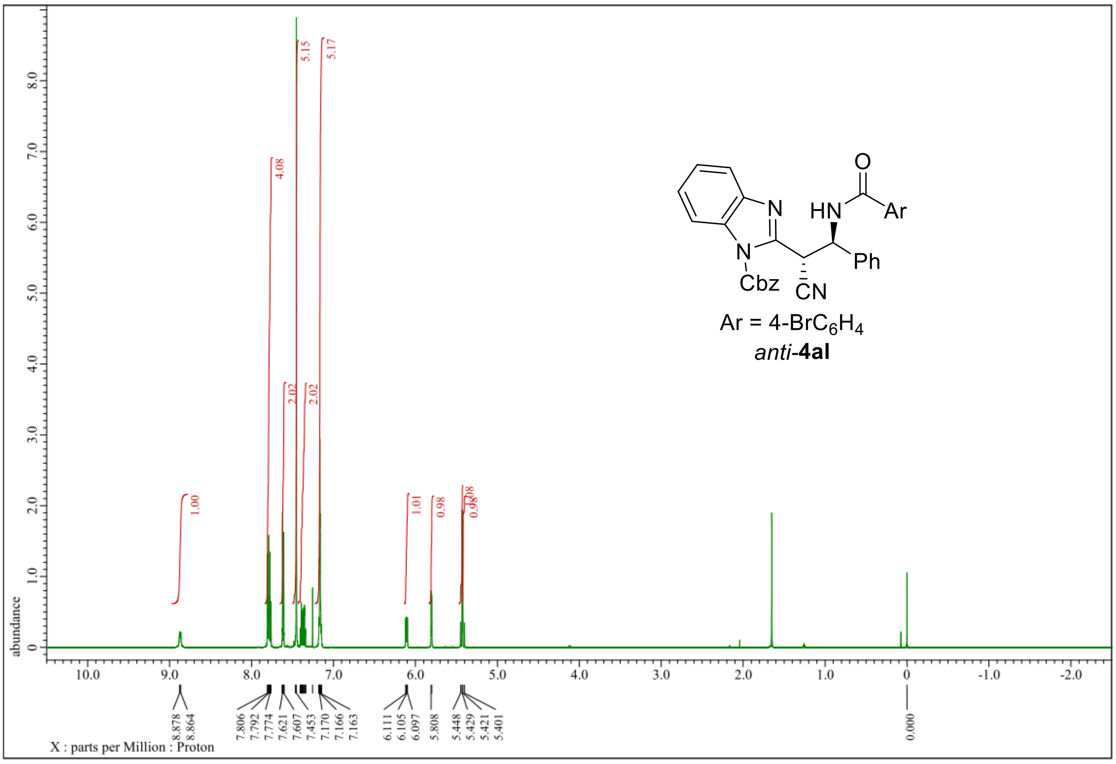

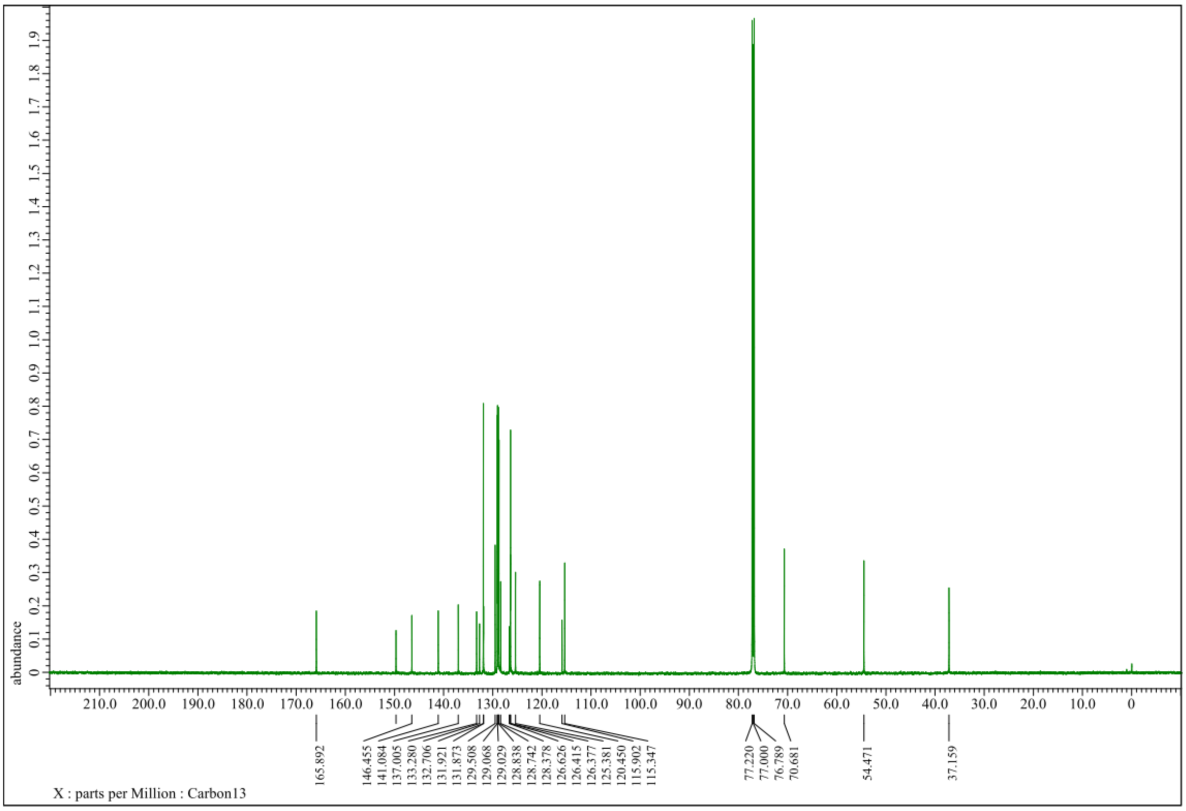

^{1}H NMR (600 MHz, $CDCl_3$) and ^{13}C NMR (151 MHz, $CDCl_3$) spectra of *anti*-**4am**

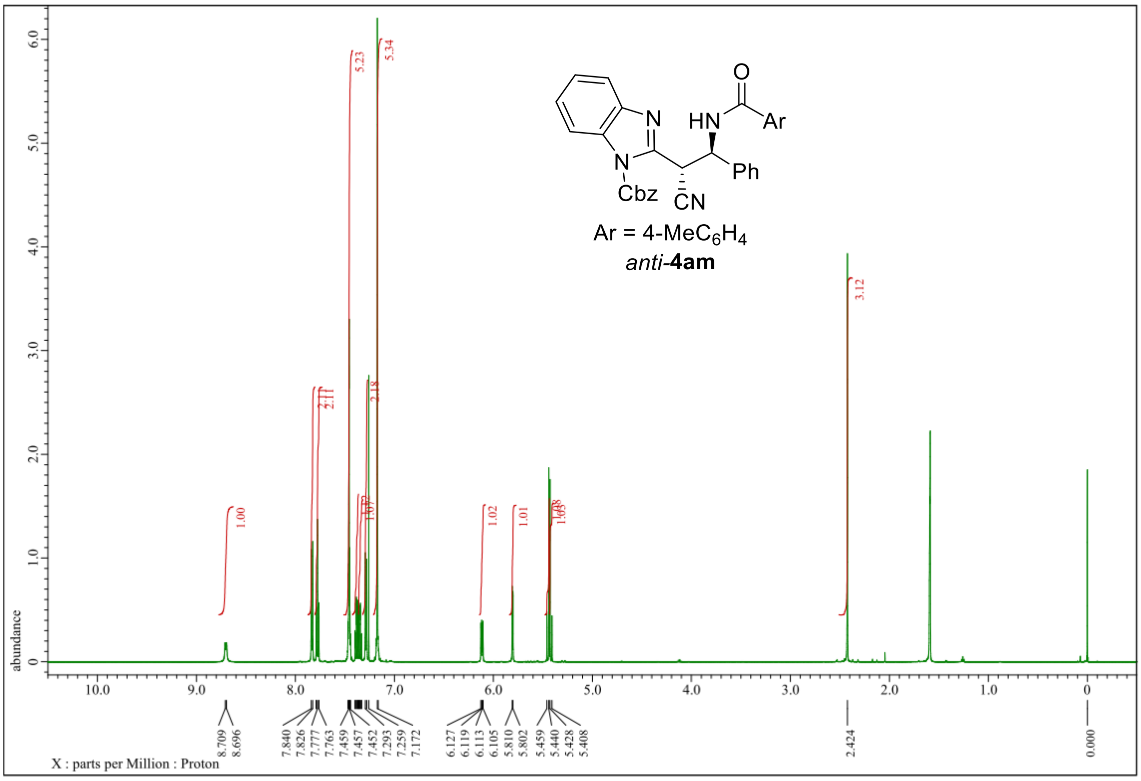

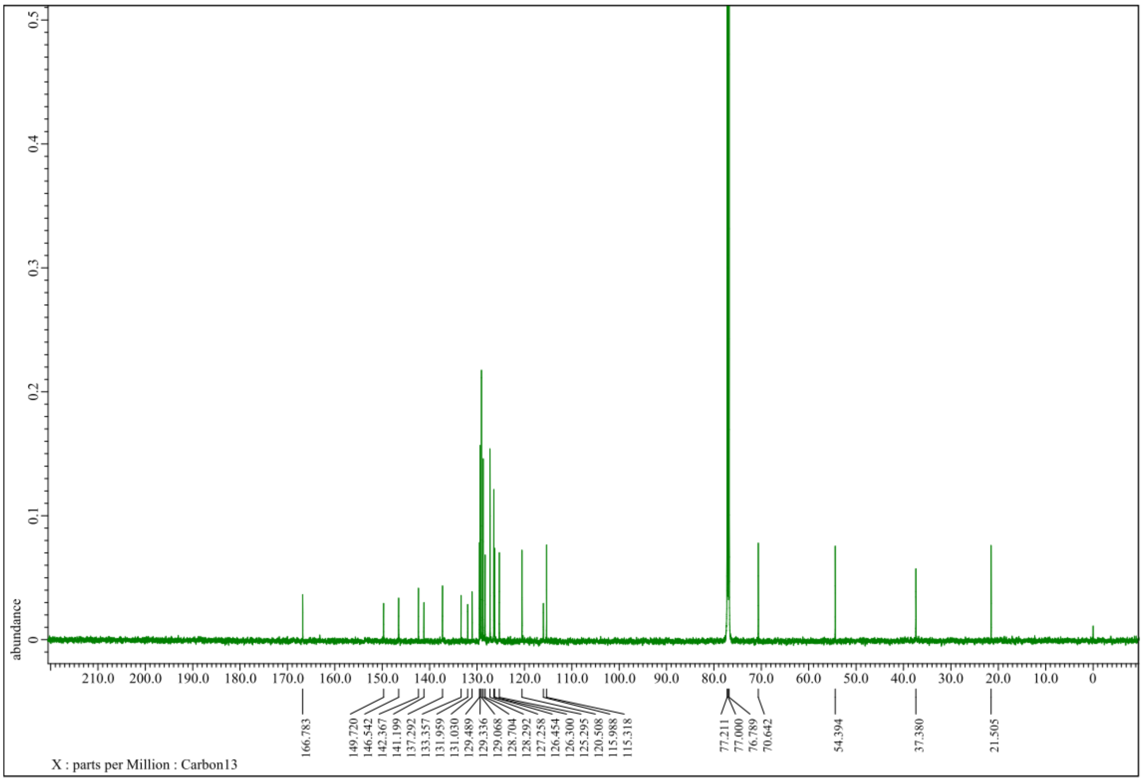

^{1}H NMR (600 MHz, $CDCl_3$) and ^{13}C NMR (151 MHz, $CDCl_3$) spectra of **4an**

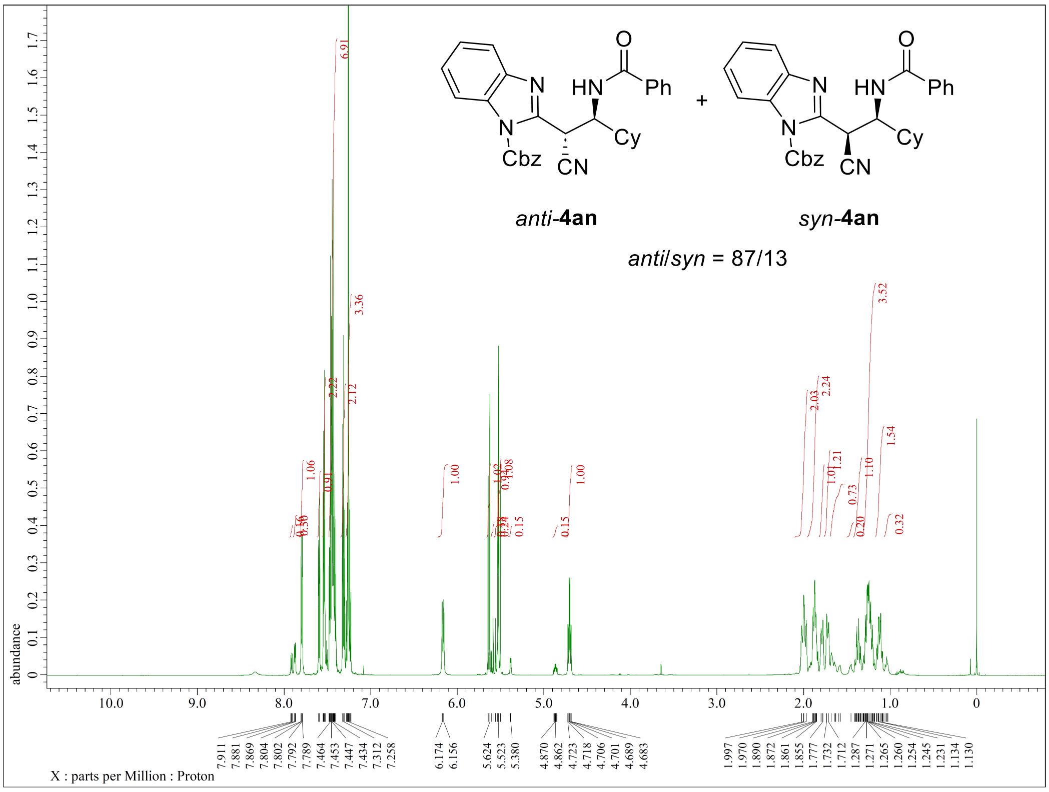

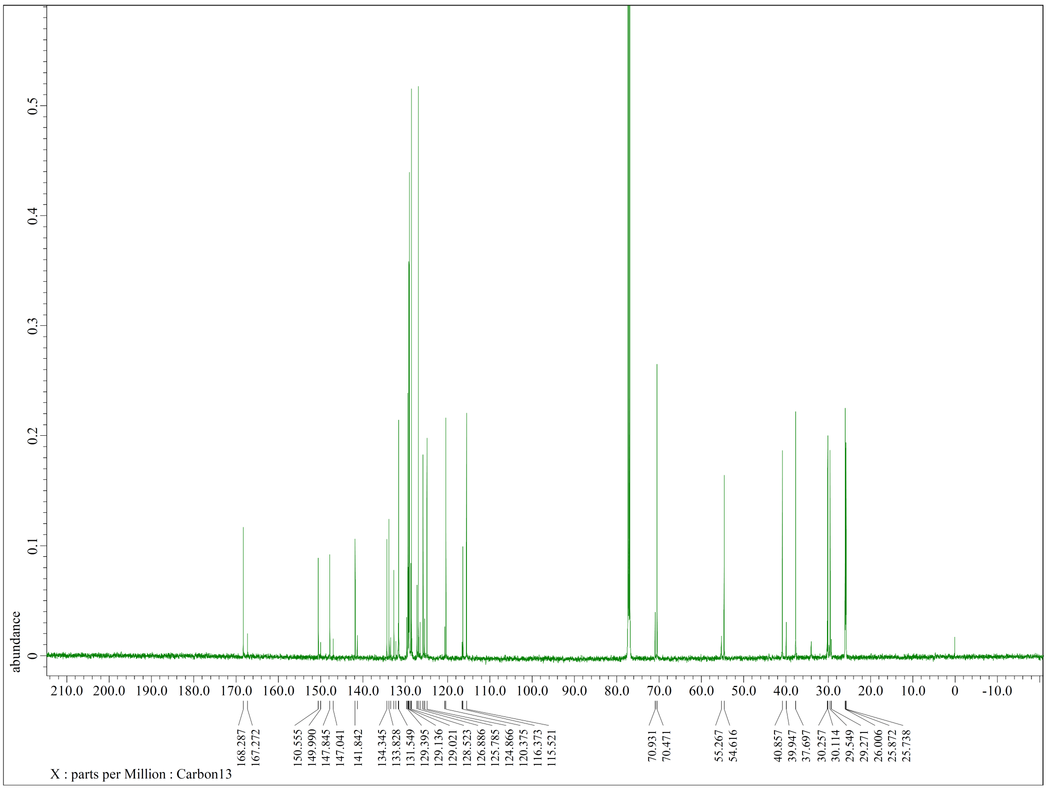

^{1}H NMR (600 MHz, $CDCl_3$) and ^{13}C NMR (151 MHz, $CDCl_3$) spectra of *anti*-**4ca**

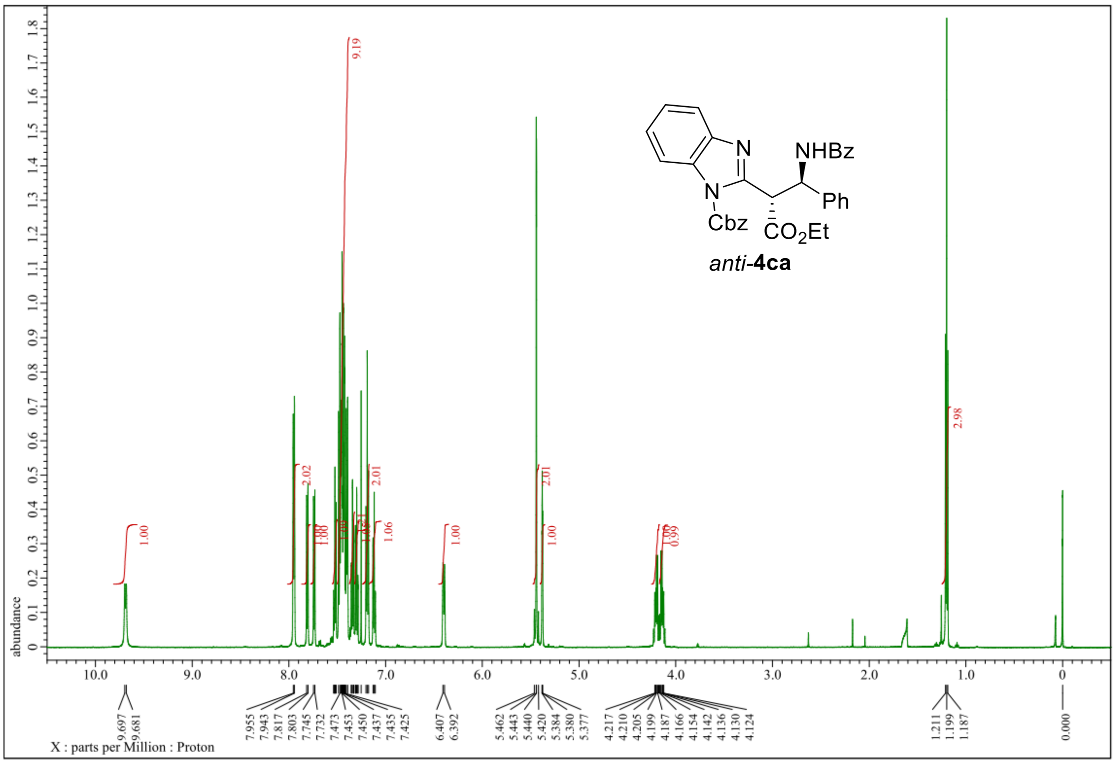

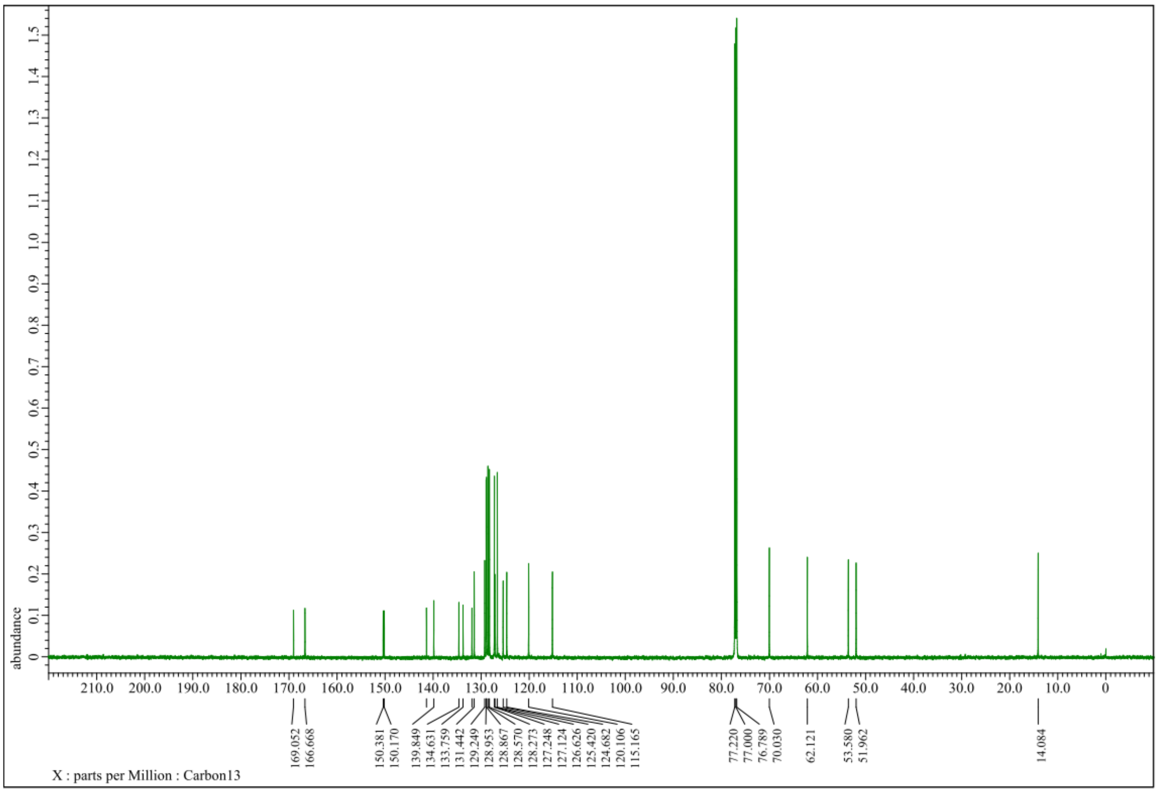

10. HPLC Charts

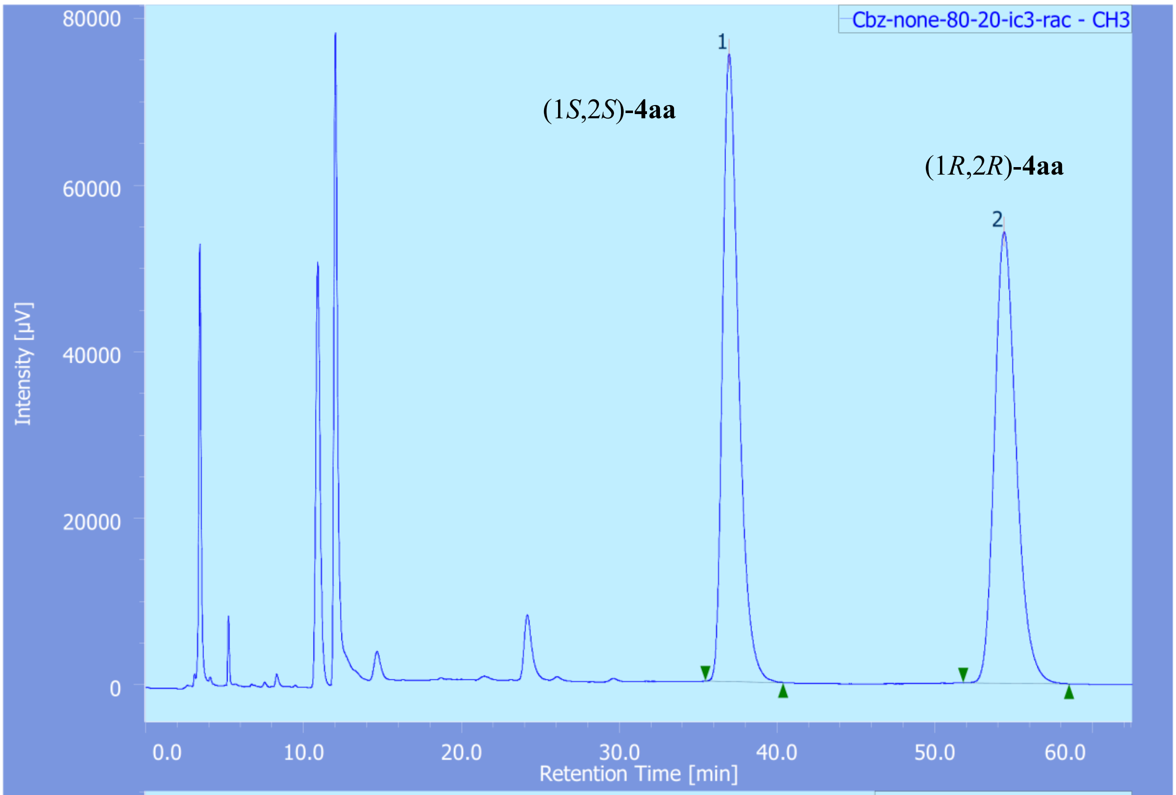

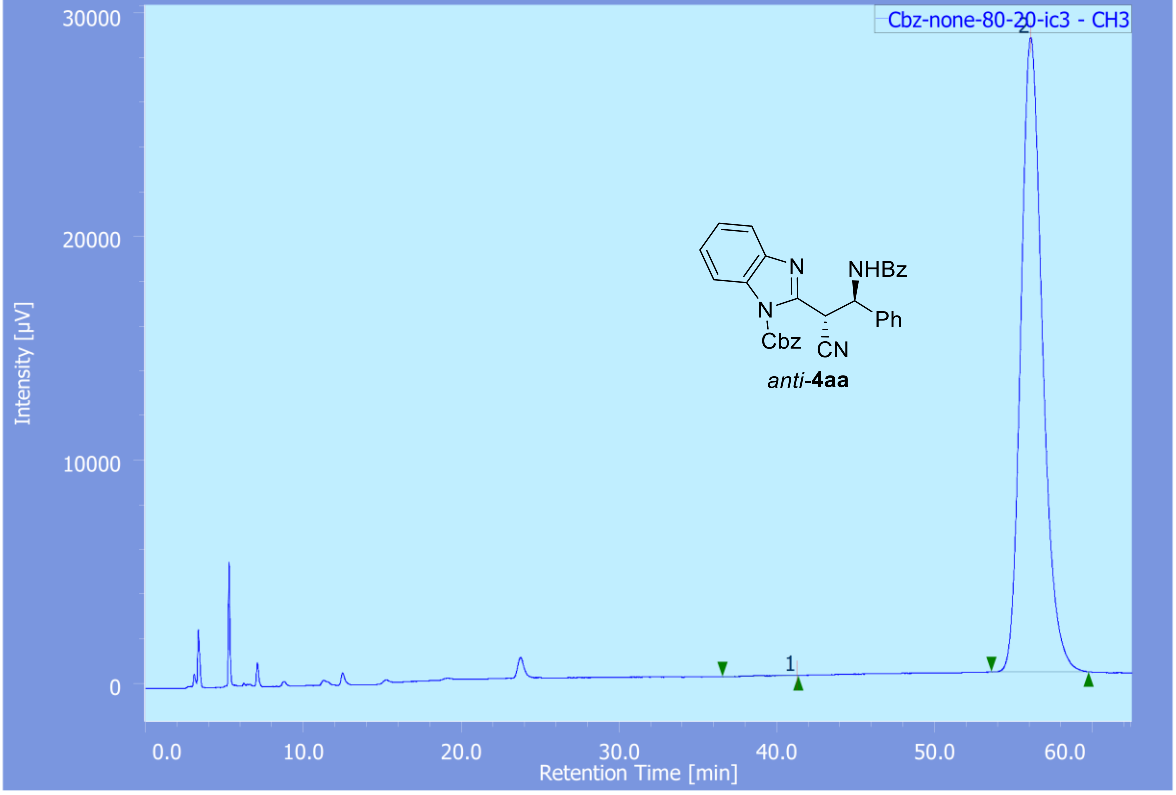

CHIRALPAK IC-3	Retention time (1)	Retention time (2)	Area (1)	Area (2)	% Area (1)	% Area (2)
anti-(±)-**4aa**	38.4	56.0	3174311	3106564	50.5	49.5
(1*R*,2*R*)-**4aa**	38.4	56.0	2278	2704528	0.1	99.9

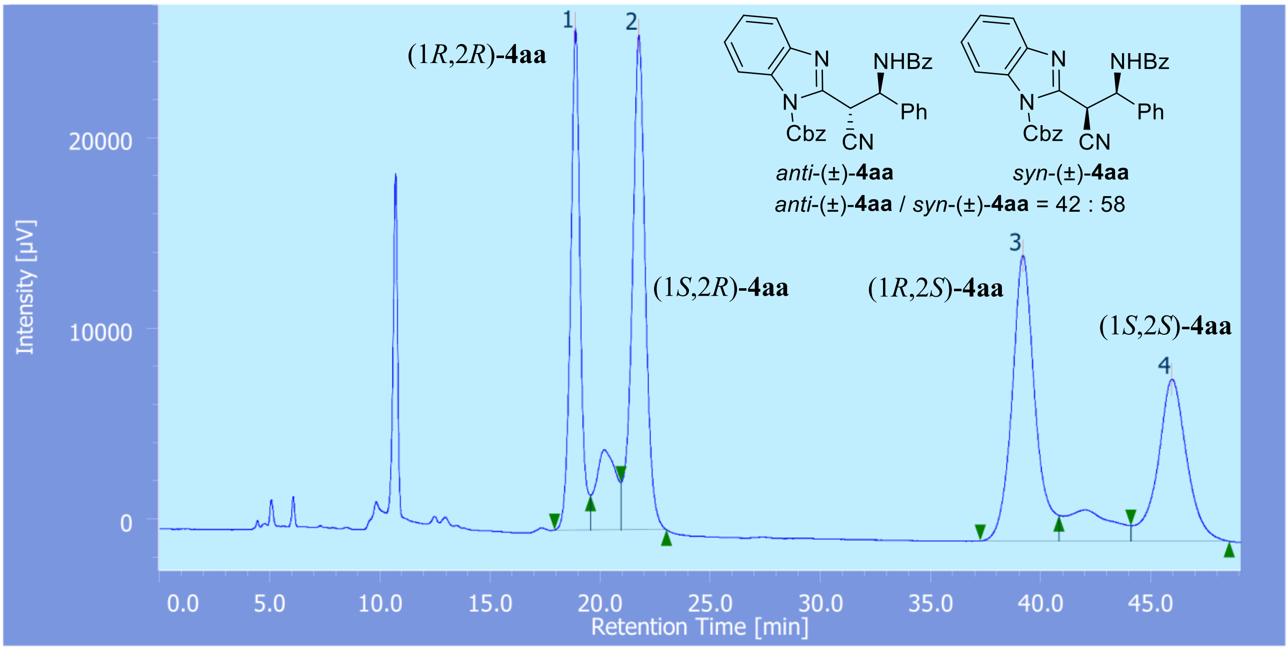

	Retention time	Area	% Area
anti- (1*R*,2*R*)-**4aa**	18.9	794490	21.273
syn-(1*S*,2*R*)-**4aa**	21.8	1074779	28.778
syn-(1*R*,2*S*)-**4aa**	39.2	1086514	29.092
anti-(1*S*,2*S*)-**4aa**	45.9	778951	20.857

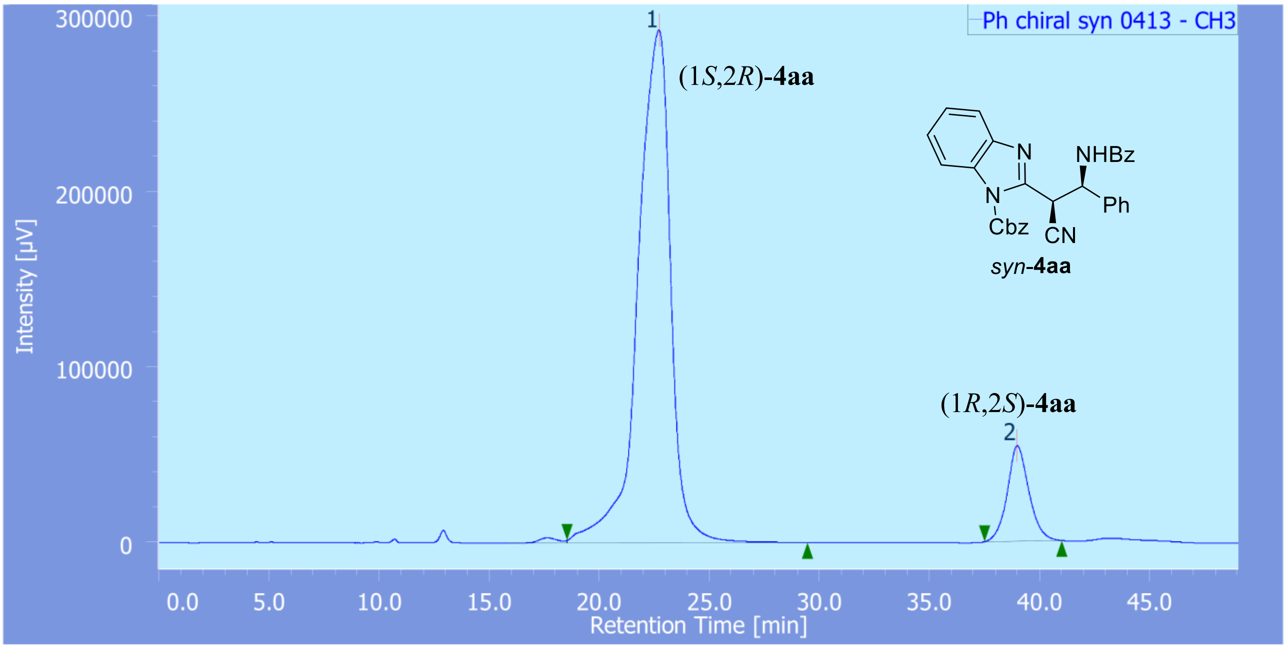

CHIRALPAK IA	Retention time	Area	% Area
syn-(1*S*,2*R*)-**4aa**	21.7	30086148	94.654
syn-(1*R*,2*S*)-**4aa**	38.9	1699325	5.346

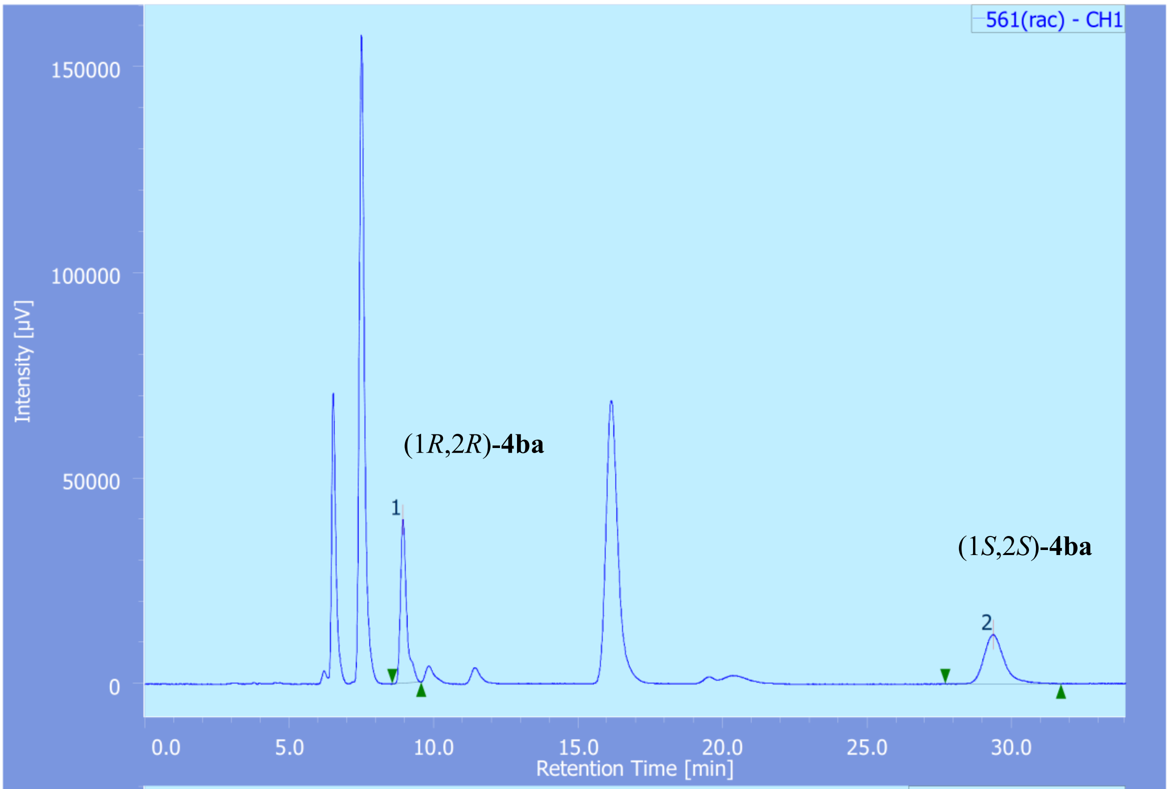

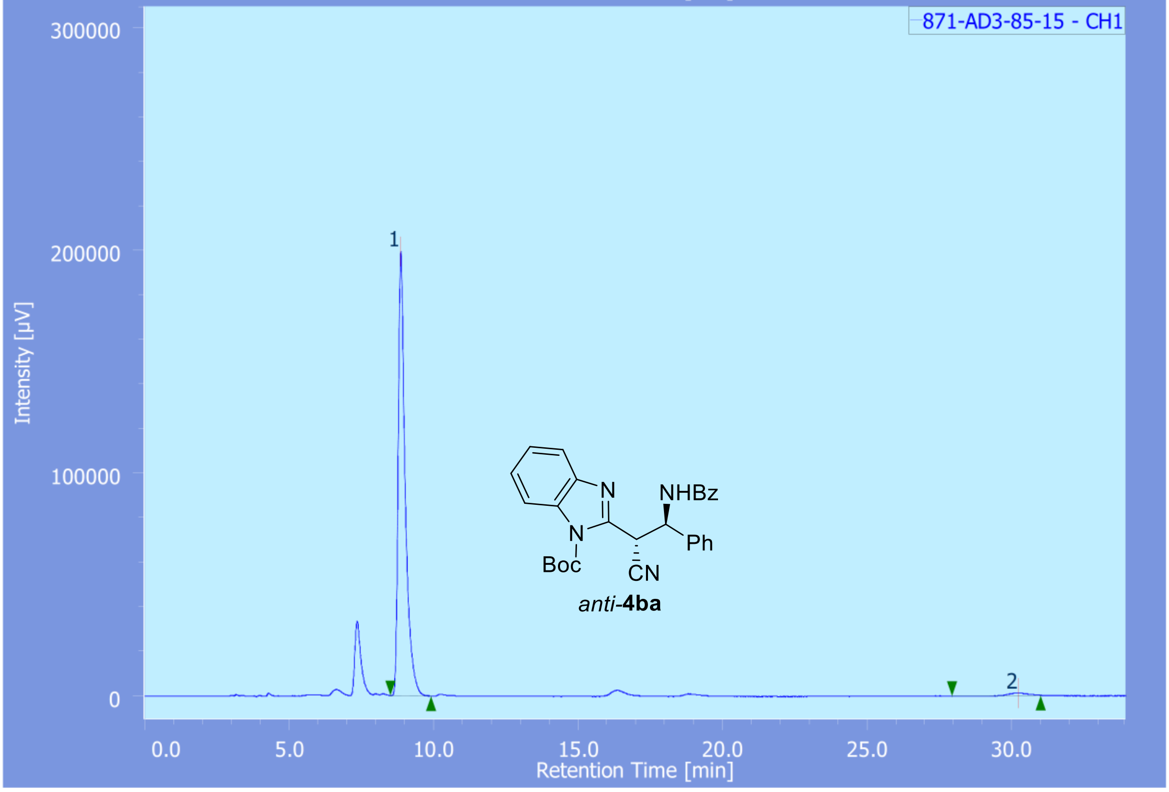

CHIRALPAK AD-3	Retention time (1)	Retention time (2)	Area (1)	Area (2)	% Area (1)	% Area (2)
(±)-**4ba**	8.9	29.4	595097	571549	51.0	49.0
(1*R*,2*R*)-**4ba**	8.8	29.4	3386017	49915	98.5	1.5

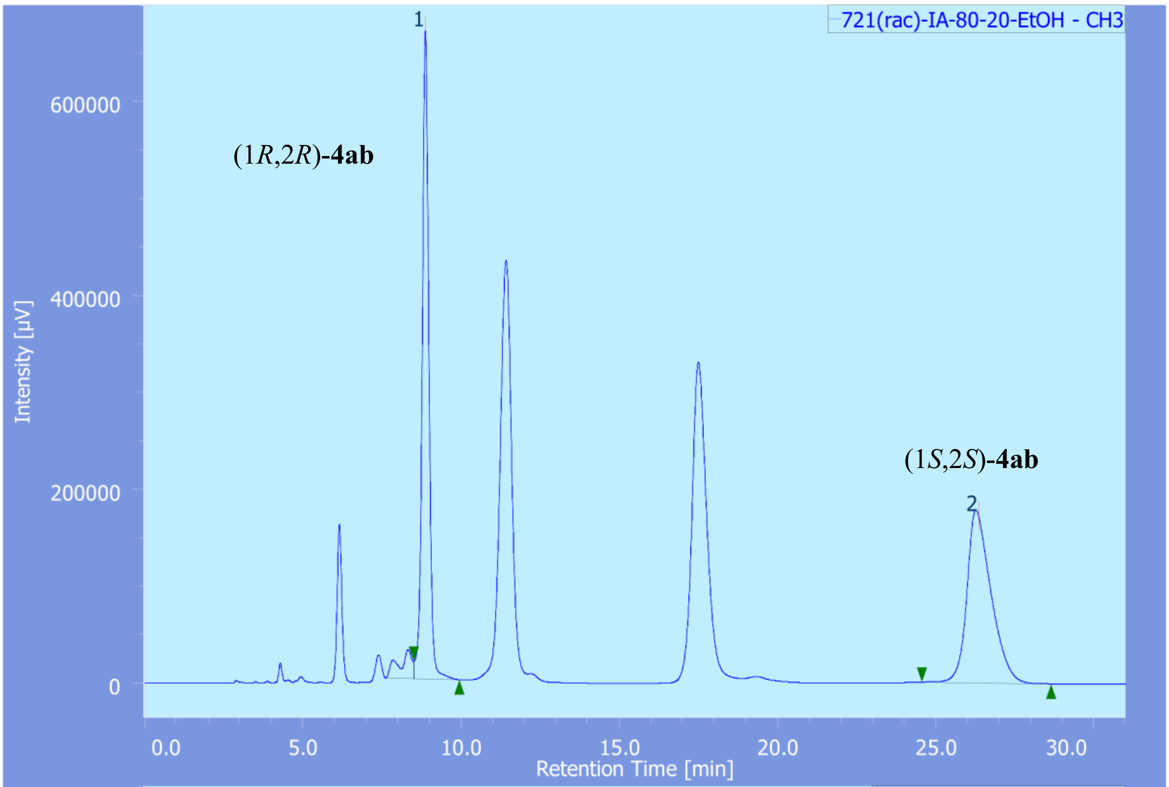

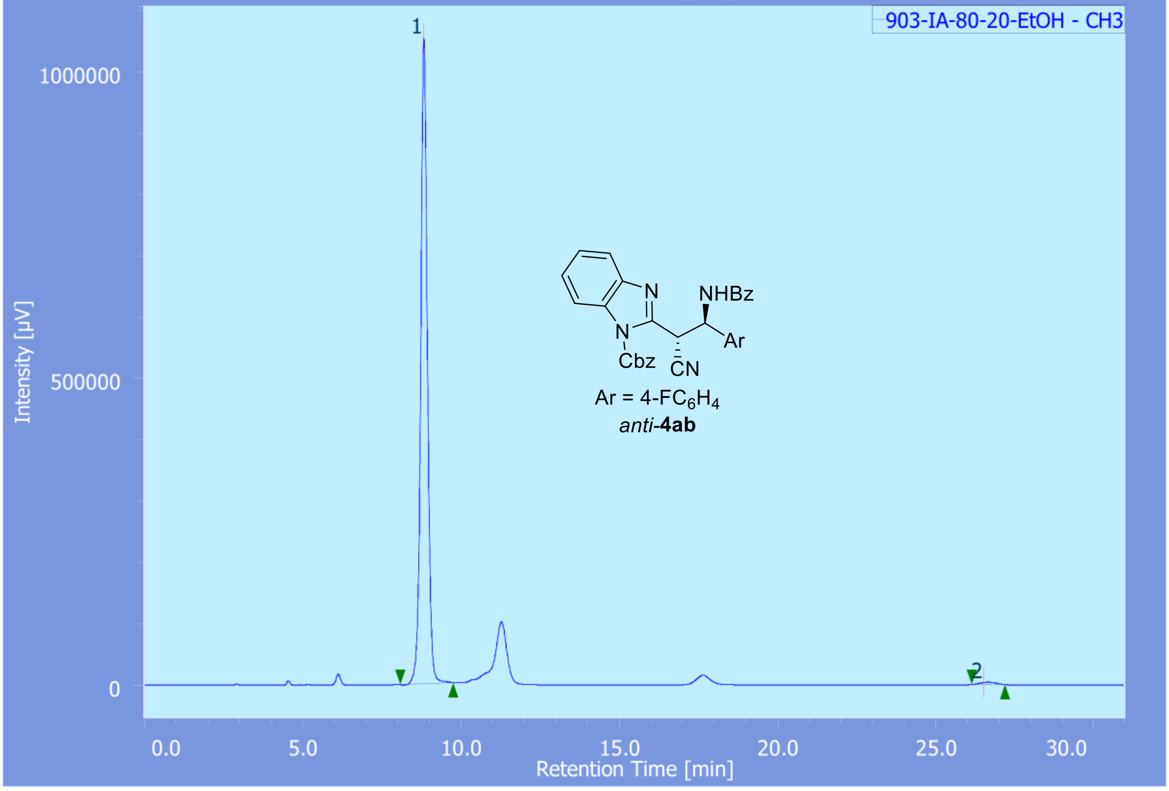

CHIRALPAK IA	Retention time (1)	Retention time (2)	Area (1)	Area (2)	% Area (1)	% Area (2)
(±)-**4ab**	8.8	26.5	9747905	9399544	50.9	49.1
(1*R*,2*R*)-**4ab**	8.8	26.5	15404114	128571	99.2	0.8

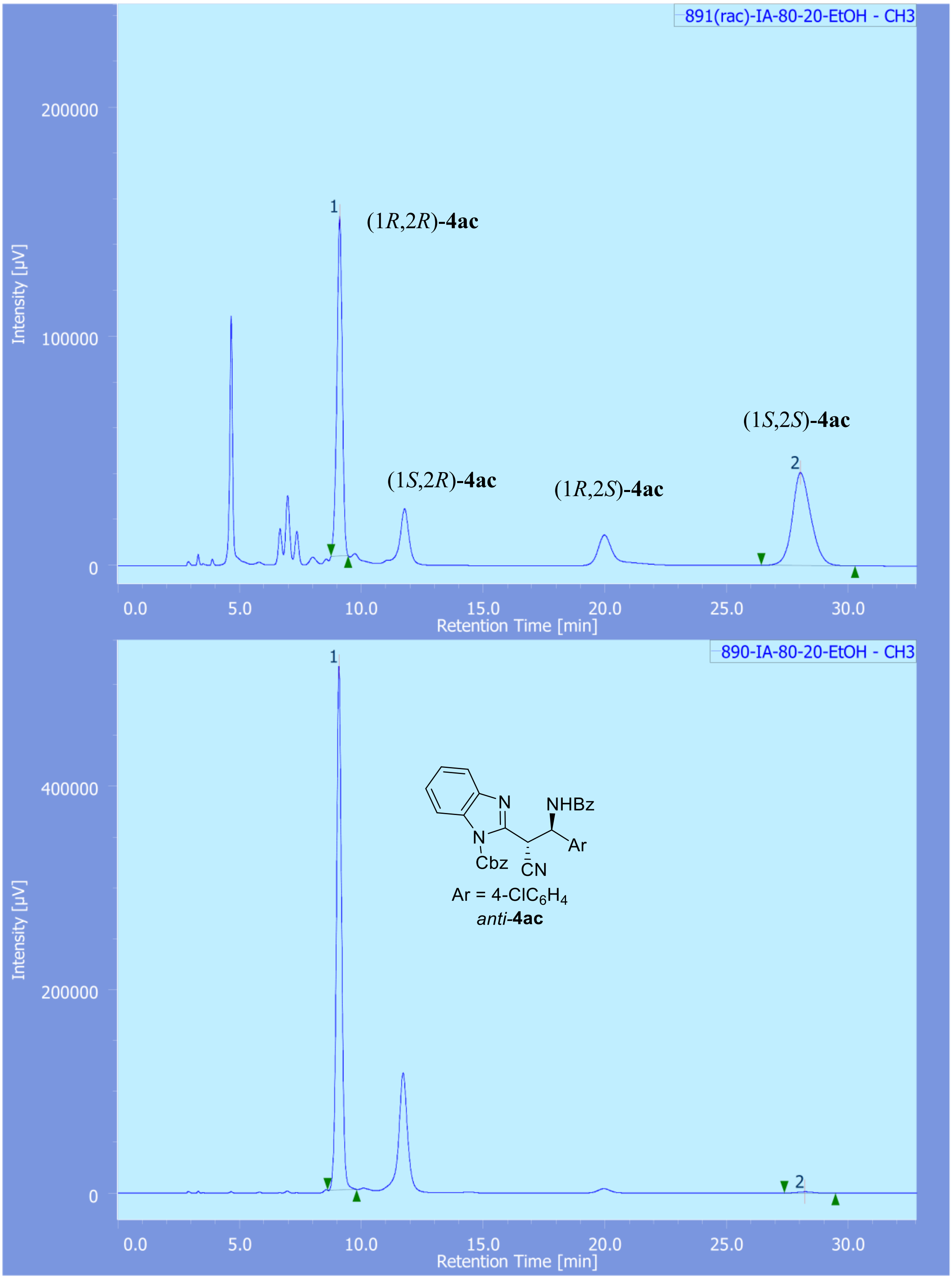

CHIRALPAK IA	Retention time (1)	Retention time (2)	Area (1)	Area (2)	% Area (1)	% Area (2)
(±)-**4ac**	9.1	28.2	2269273	2153136	51.3	48.7
(1*R*,2*R*)-**4ac**	9.1	28.2	7598783	65079	99.2	0.8

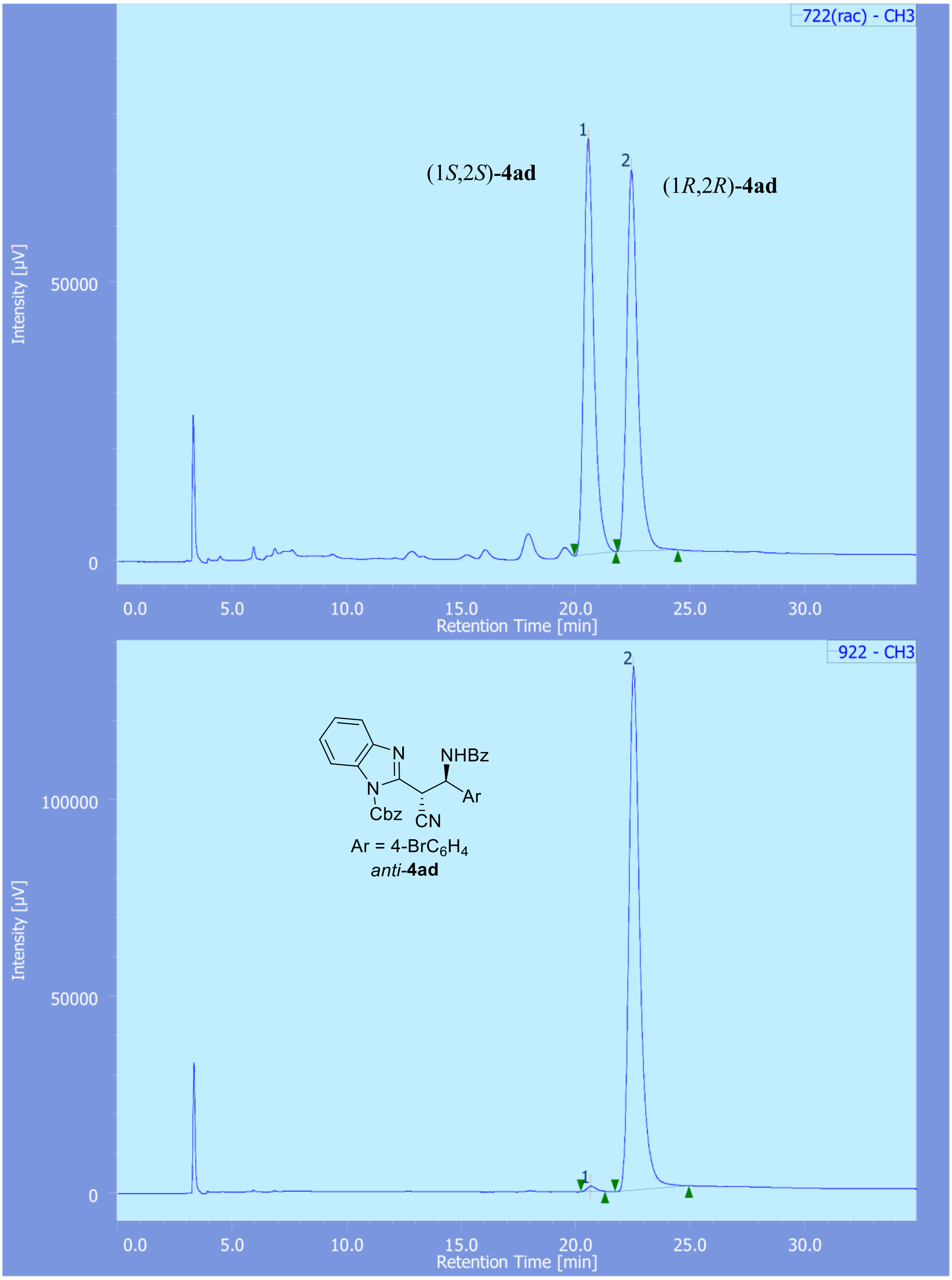

CHIRALPAK IC-3	Retention time (1)	Retention time (2)	Area (1)	Area (2)	% Area (1)	% Area (2)
(±)-**4ad**	20.6	22.4	2278380	2282501	50.0	50.0
(1*R*,2*R*)-**4ad**	20.6	22.4	37831	4456461	0.8	99.2

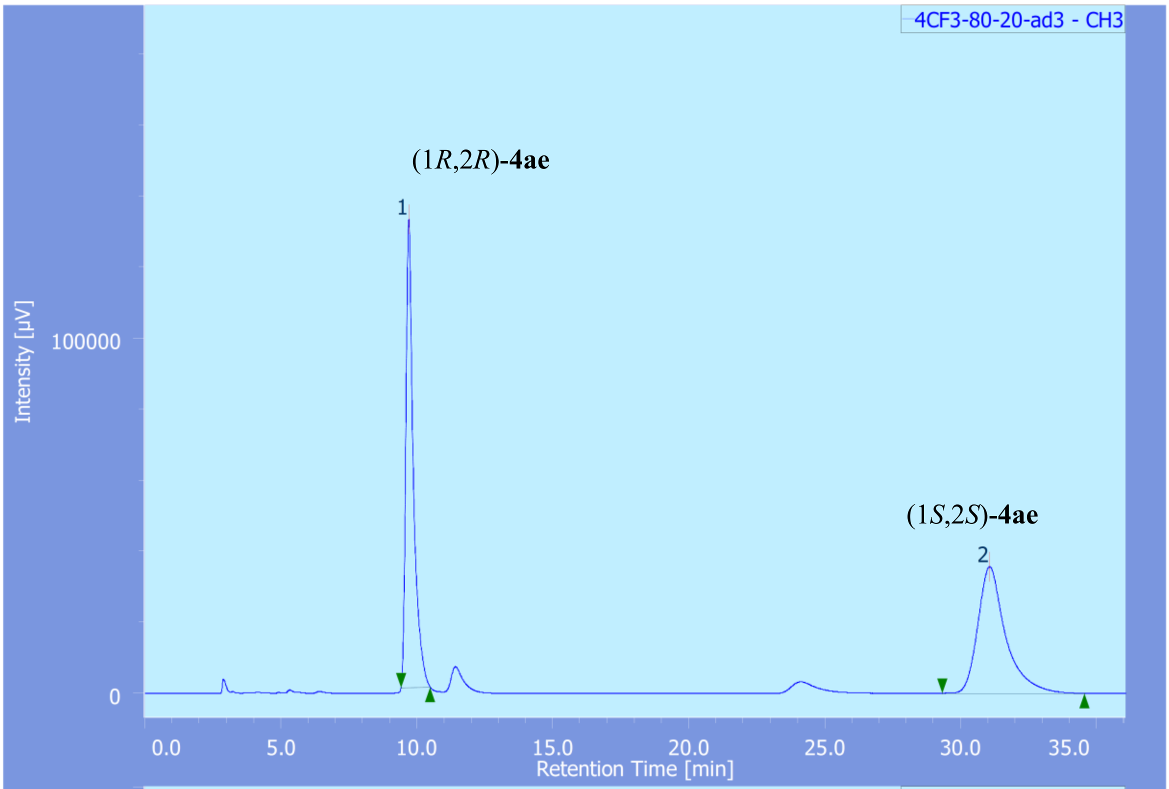

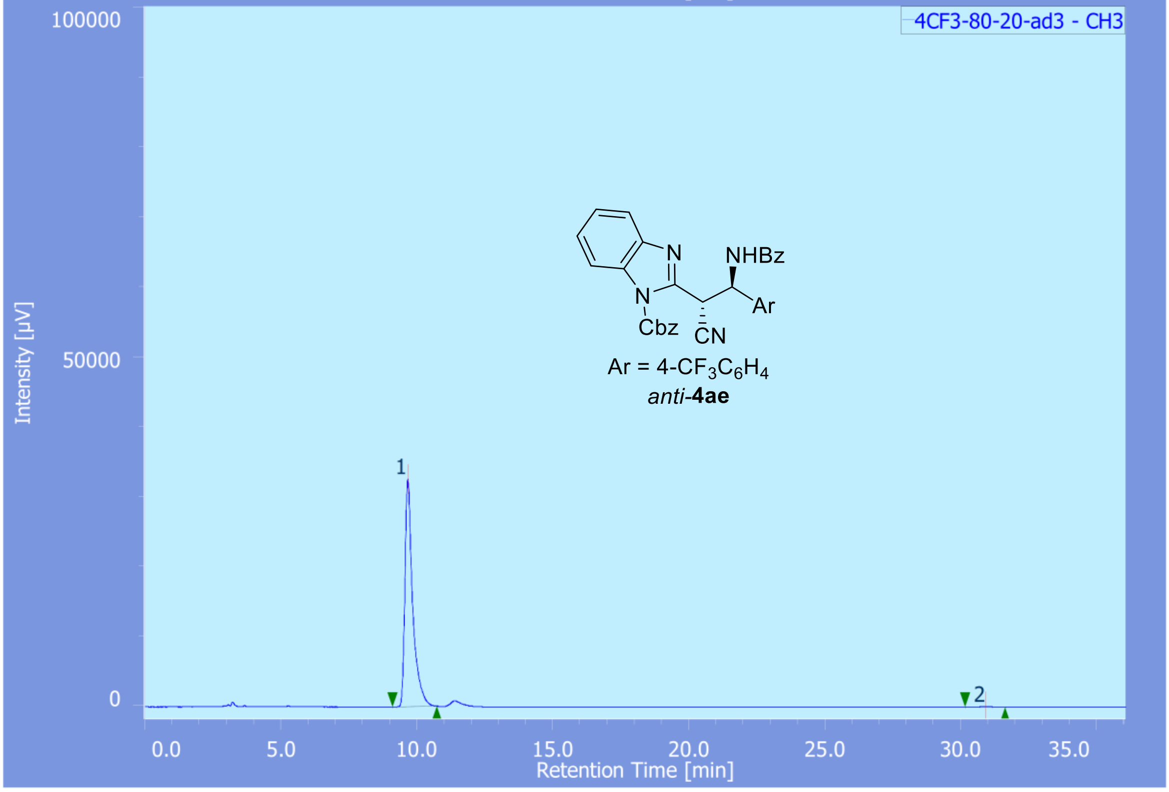

CHIRALPAK AD-3	Retention time (1)	Retention time (2)	Area (1)	Area (2)	% Area (1)	% Area (2)
(±)-**4ae**	9.7	31.0	2550119	2529213	50.2	49.8
(1*R*,2*R*)-**4ae**	9.6	30.9	644644	3235	99.5	0.5

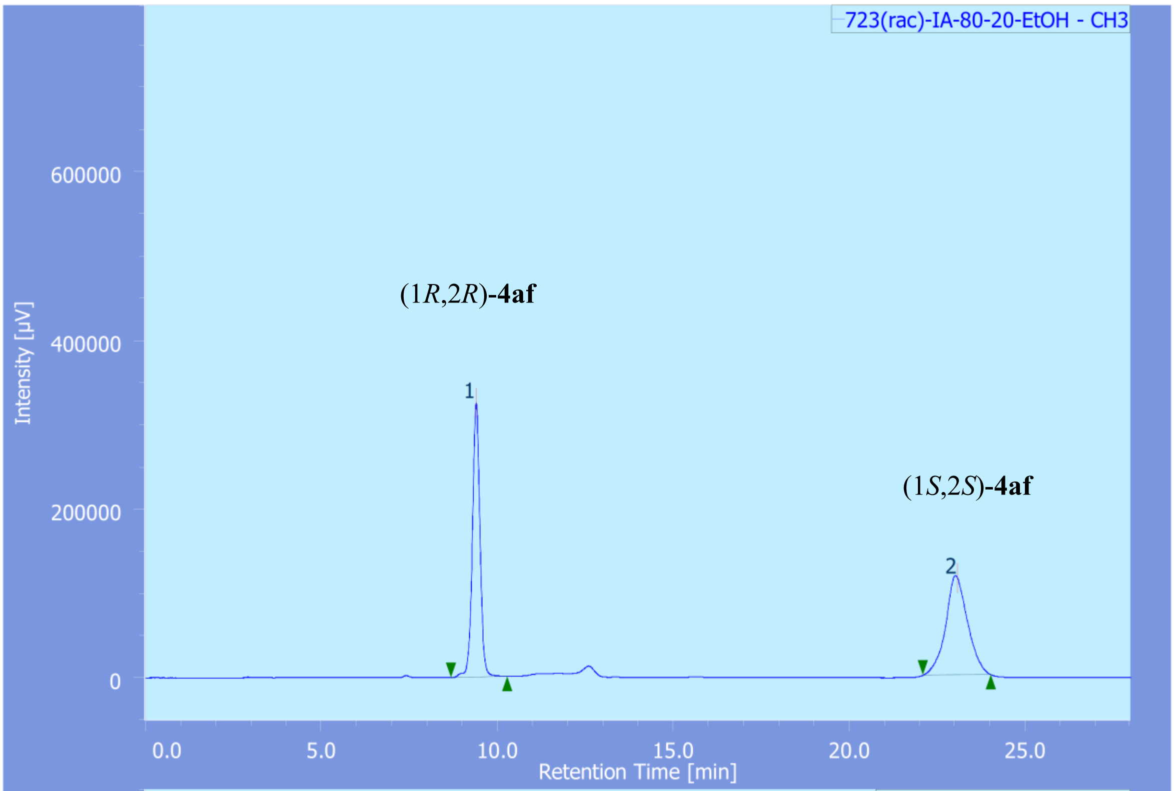

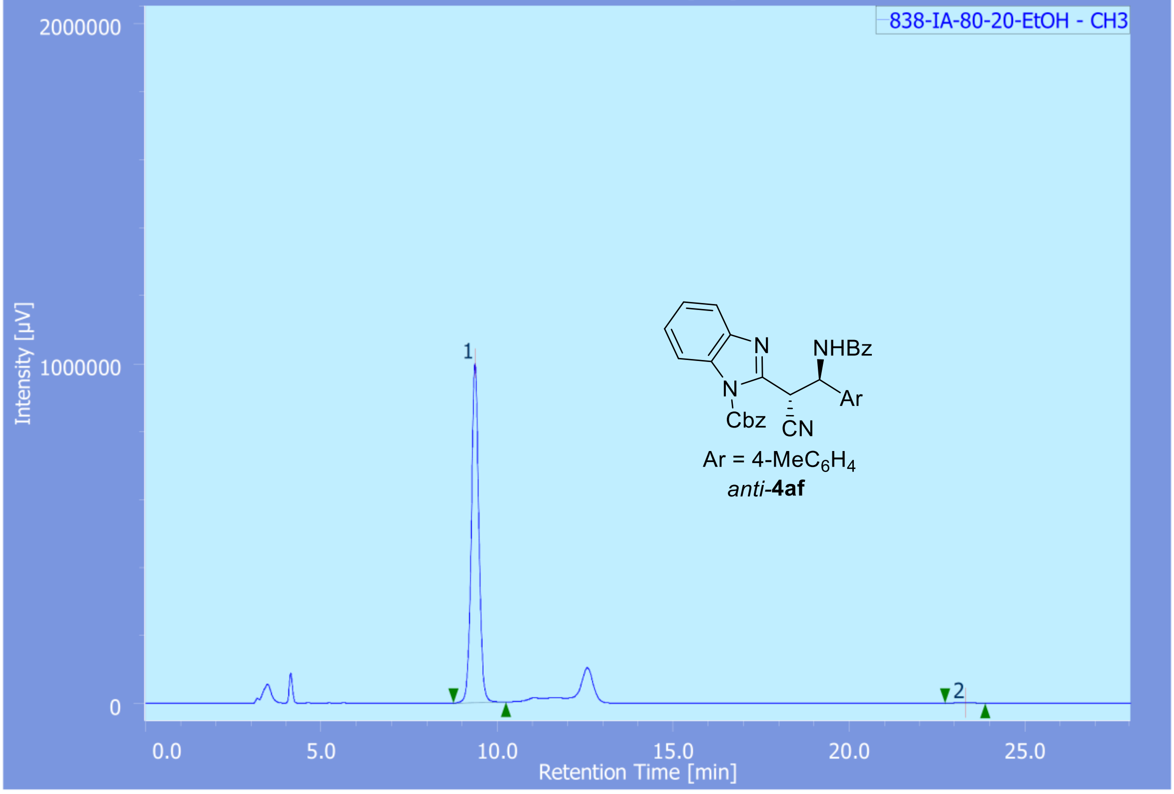

CHIRALPAK IA	Retention time (1)	Retention time (2)	Area (1)	Area (2)	% Area (1)	% Area (2)
(±)-**4af**	9.4	23.4	4915489	5095461	49.2	51.8
(1*R*,2*R*)-**4af**	9.4	23.4	14960755	76405	99.5	0.5

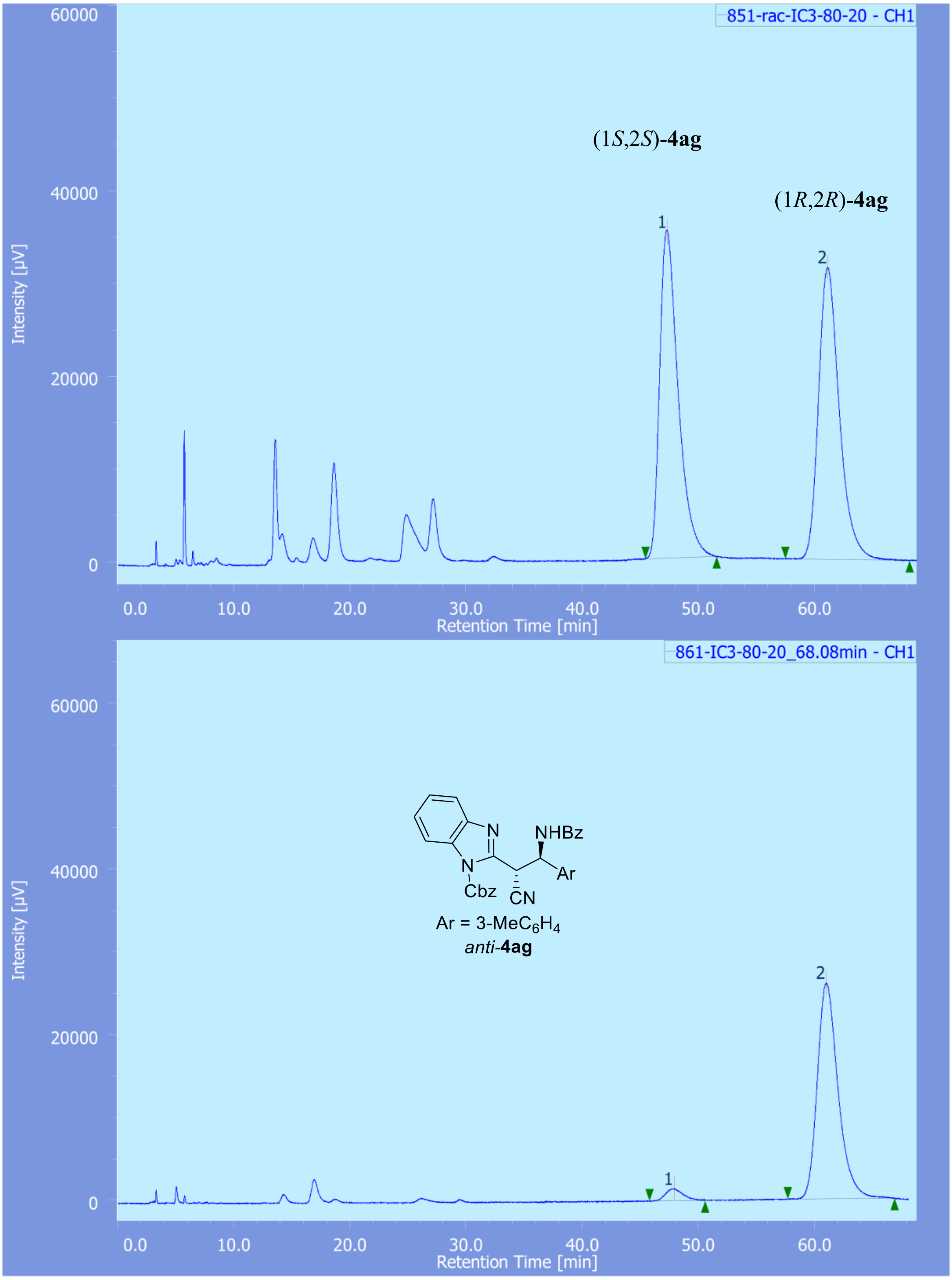

CHIRALPAK IC-3	Retention time (1)	Retention time (2)	Area (1)	Area (2)	% Area (1)	% Area (2)
(±)-**4ag**	47.9	60.9	3913509	3921309	49.9	50.1
(1*R*,2*R*)-**4ag**	47.9	60.9	13200	3190516	4.1	95.9

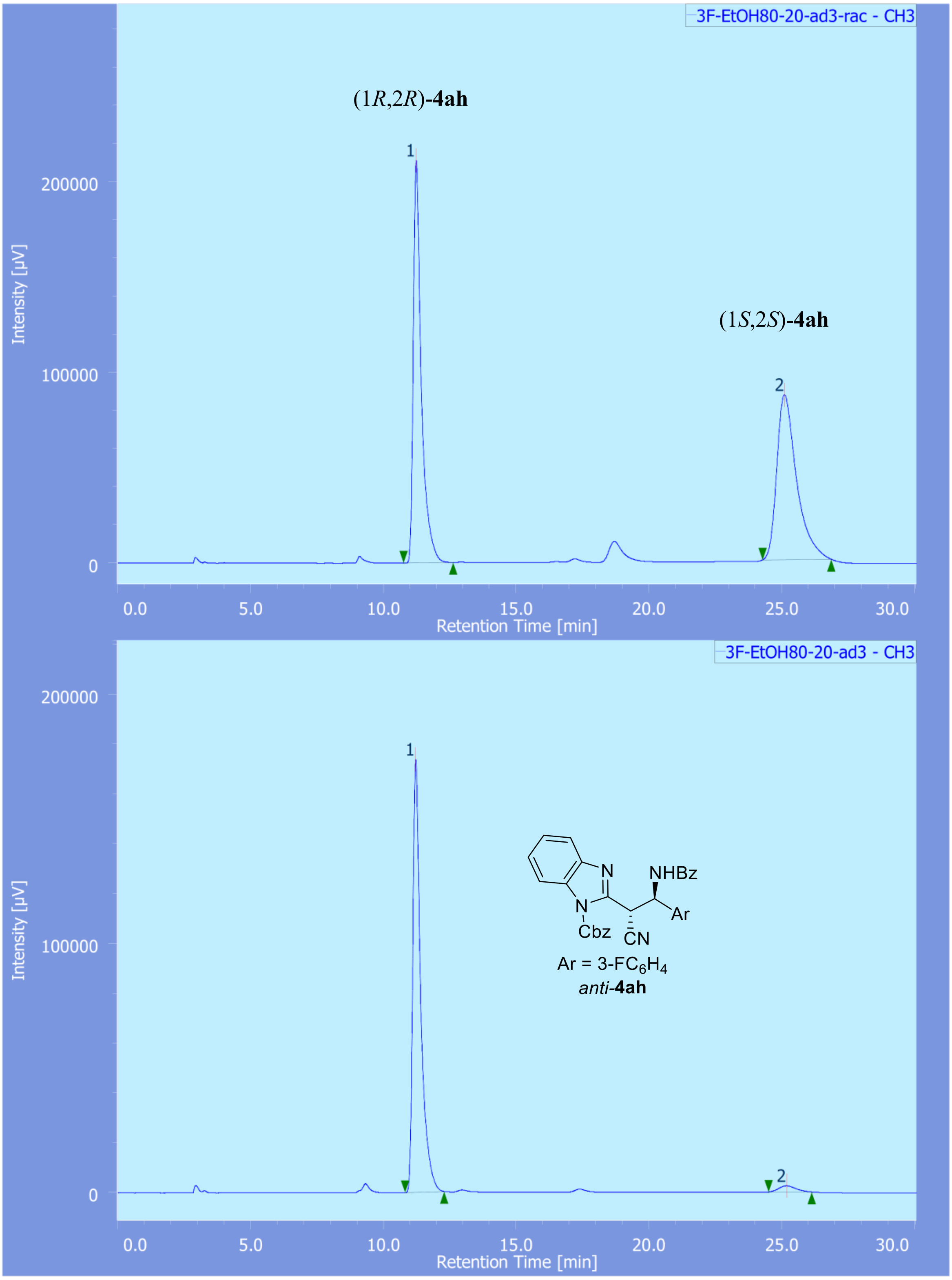

CHIRALPAK AD-3	Retention time (1)	Retention time (2)	Area (1)	Area (2)	% Area (1)	% Area (2)
(±)-**4ah**	11.2	25.0	4415320	4478299	49.6	50.4
(1*R*,2*R*)-**4ah**	11.2	25.1	3606022	109228	97.0	3.0

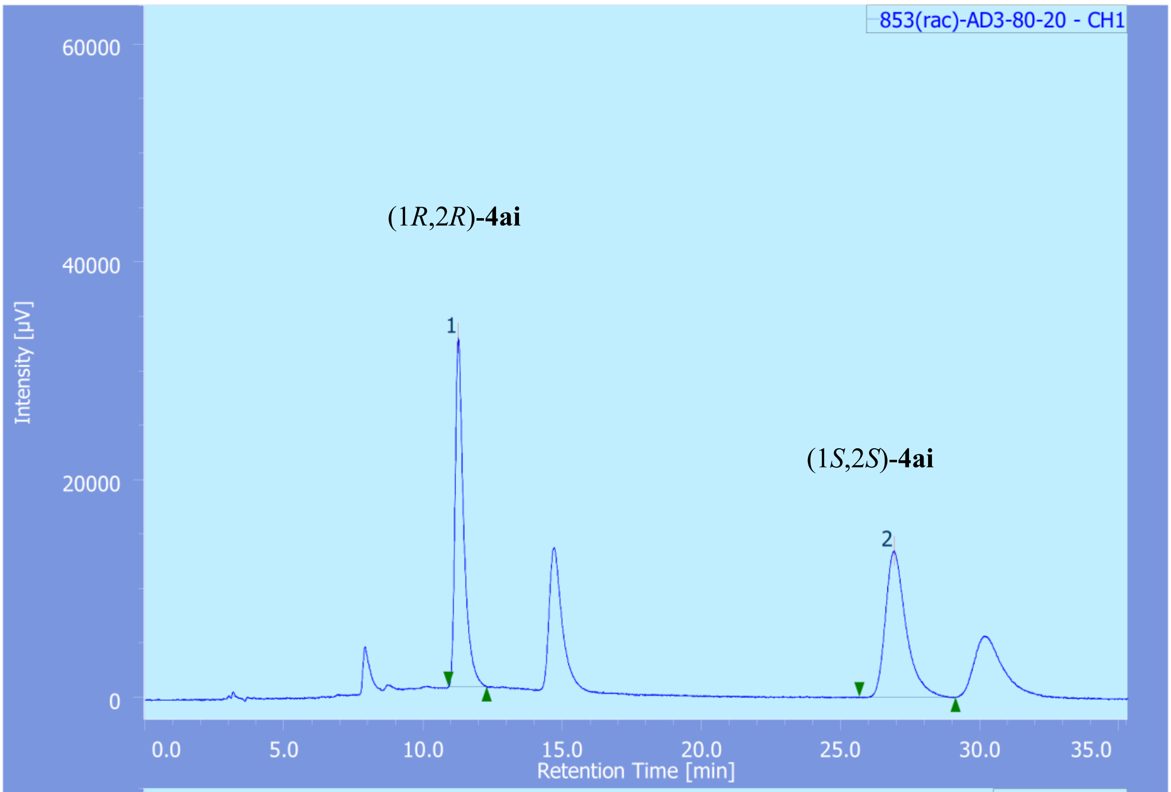

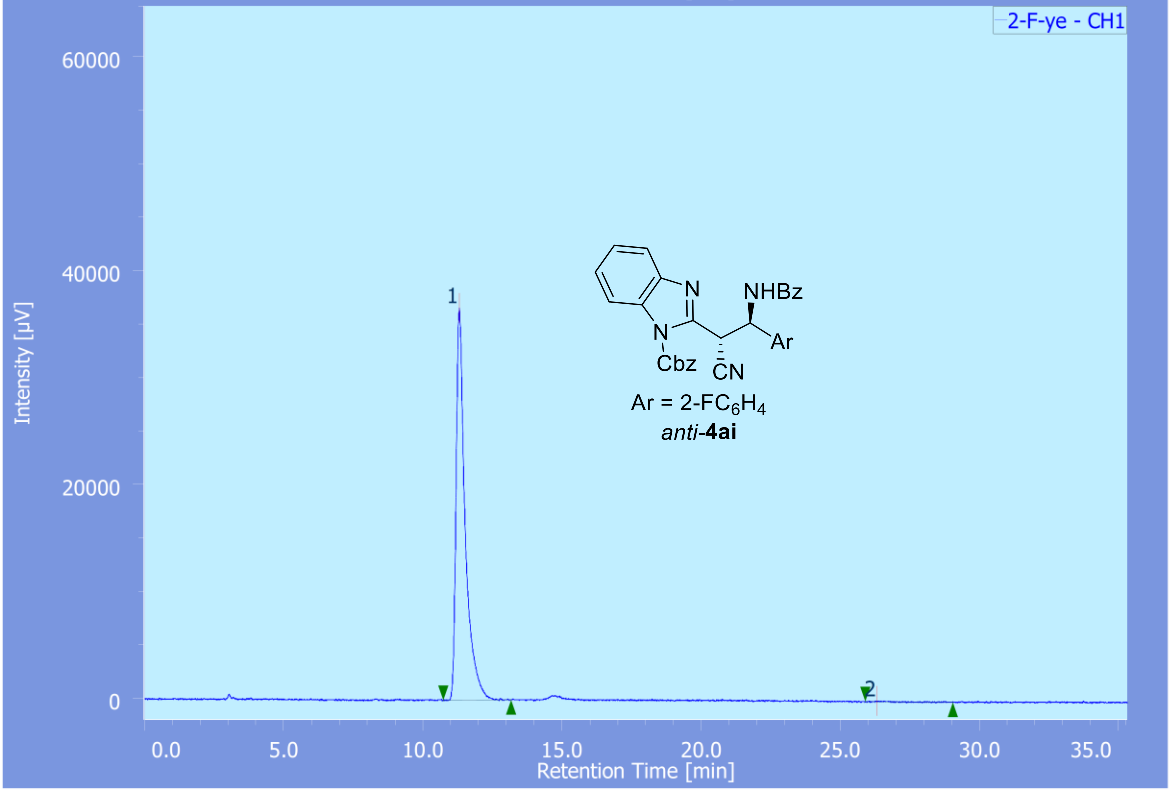

CHIRALPAK AD-3	Retention time (1)	Retention time (2)	Area (1)	Area (2)	% Area (1)	% Area (2)
(±)-**4ai**	11.2	26.9	690476	687331	50.1	49.9
(1*R*,2*R*)-**4ai**	11.2	26.9	836789	2963	99.6	0.4

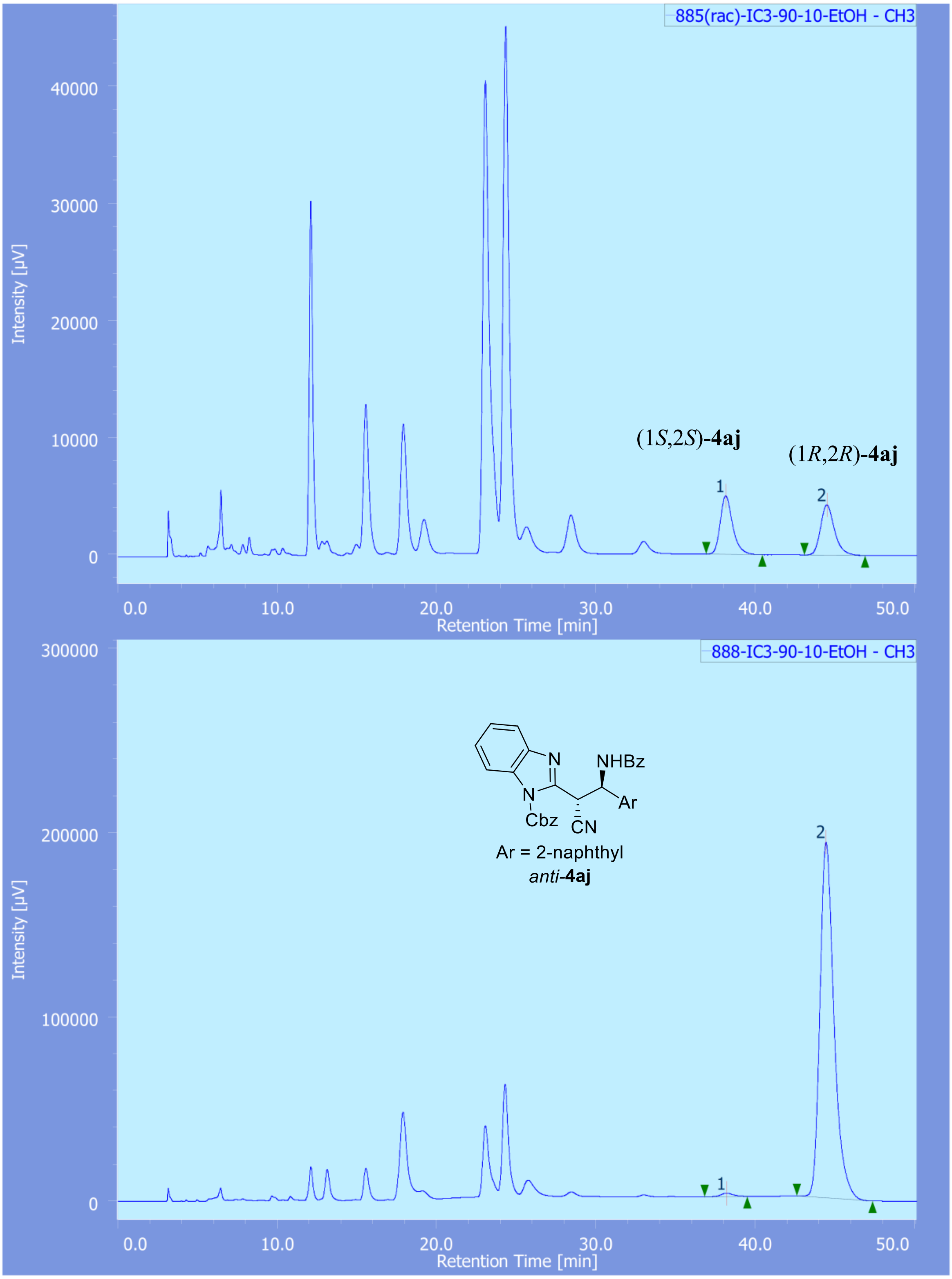

CHIRALPAK IC-3	Retention time (1)	Retention time (2)	Area (1)	Area (2)	% Area (1)	% Area (2)
(±)-**4aj**	38.1	44.7	270151	261694	50.8	49.2
(1*R*,2*R*)-**4aj**	38.1	44.7	96139	11833093	0.8	99.2

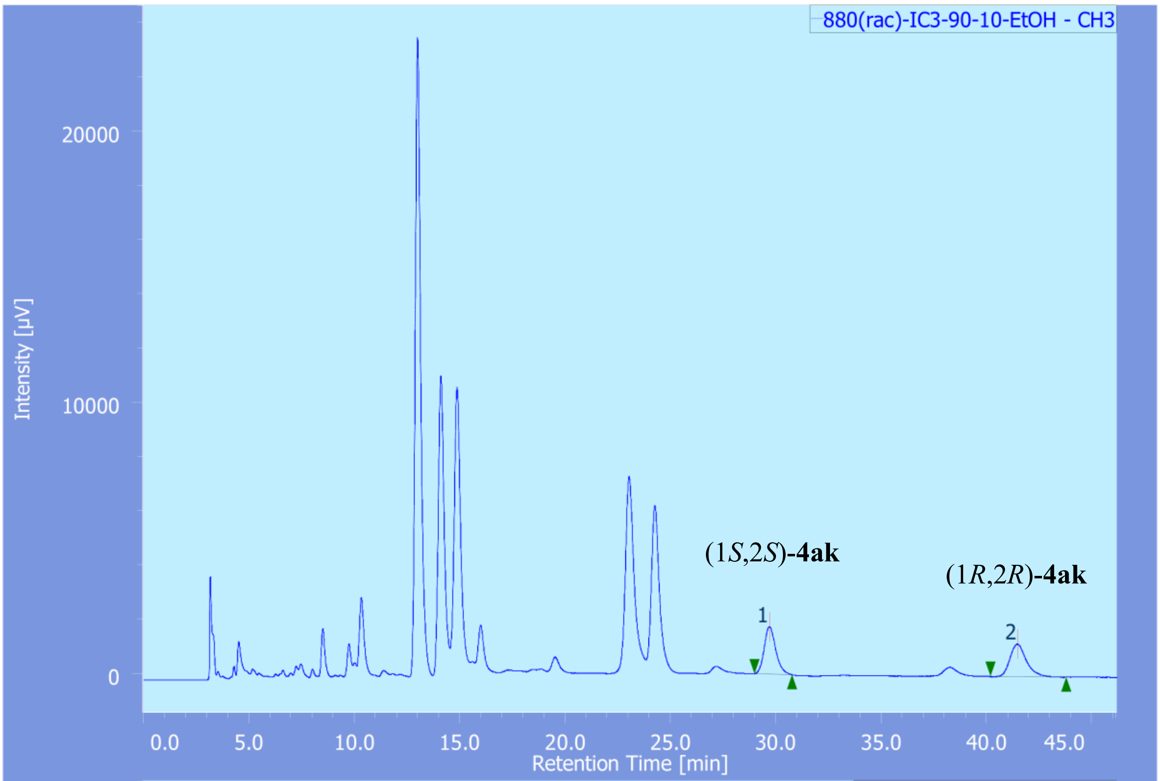

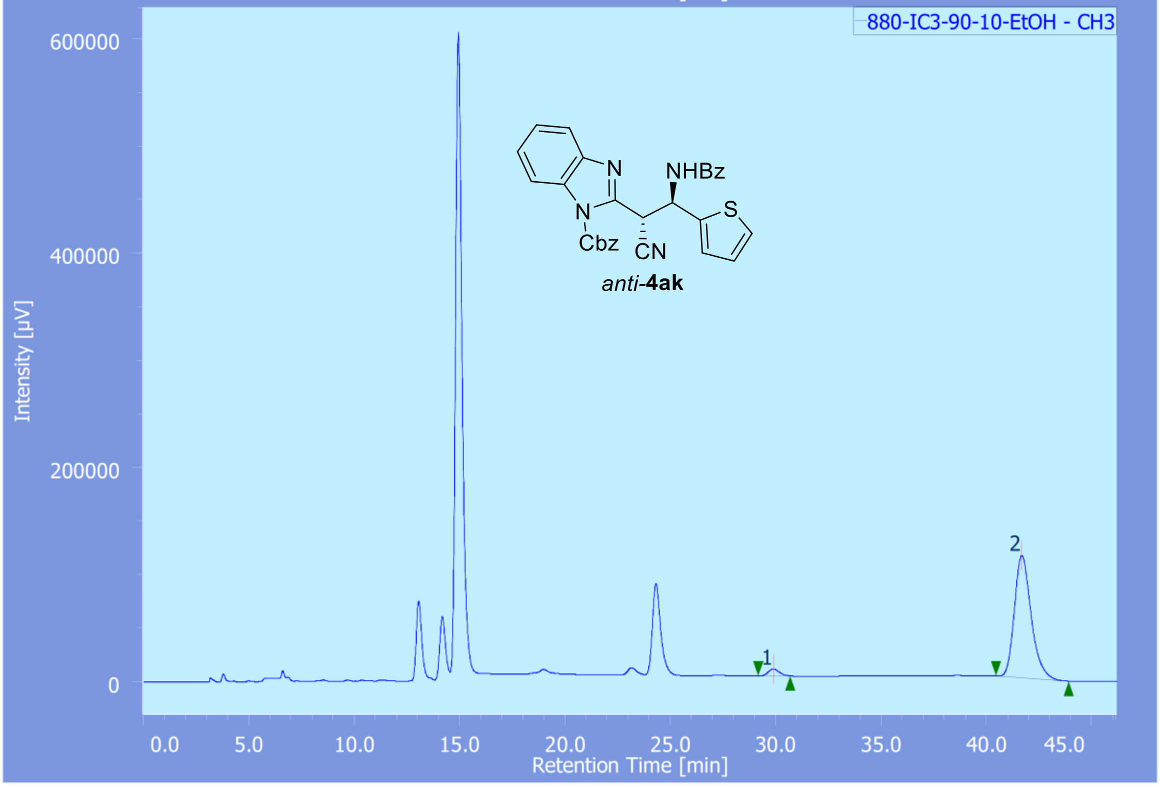

CHIRALPAK IC-3	Retention time (1)	Retention time (2)	Area (1)	Area (2)	% Area (1)	% Area (2)
(±)-**4ak**	29.8	41.6	66060	65834	50.1	49.9
(1*R*,2*R*)-**4ak**	29.8	41.6	243169	6332915	3.7	96.4

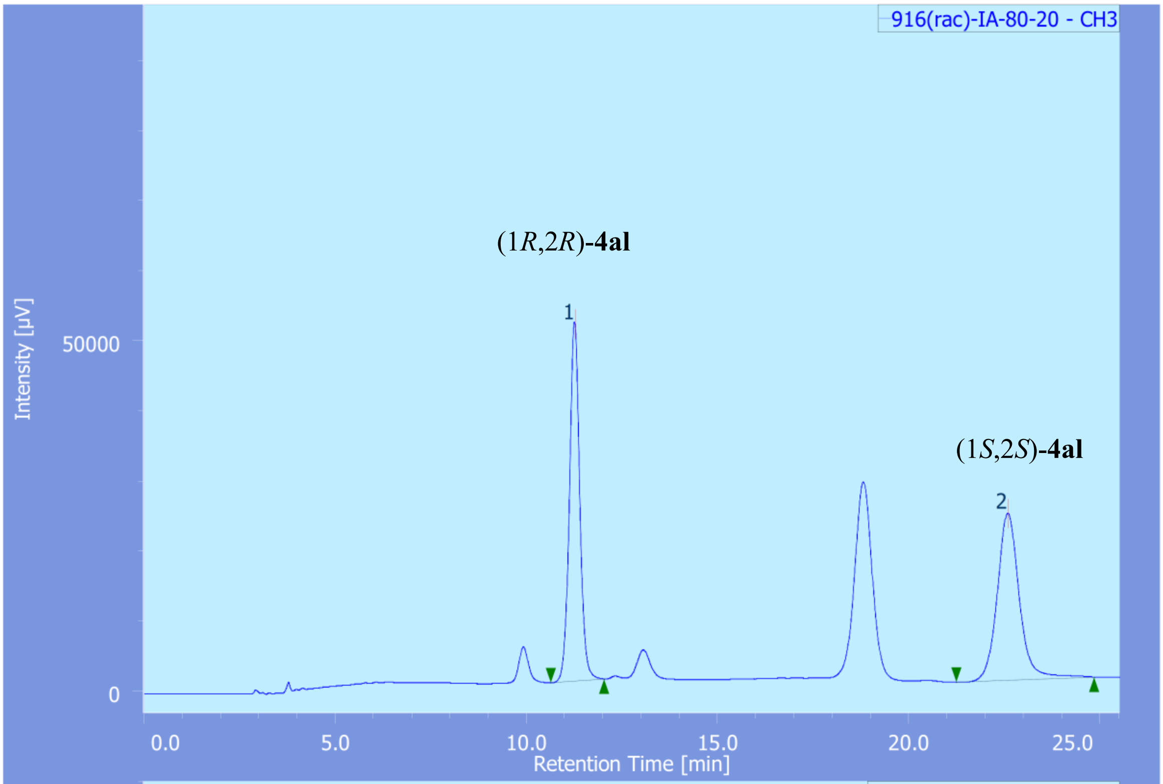

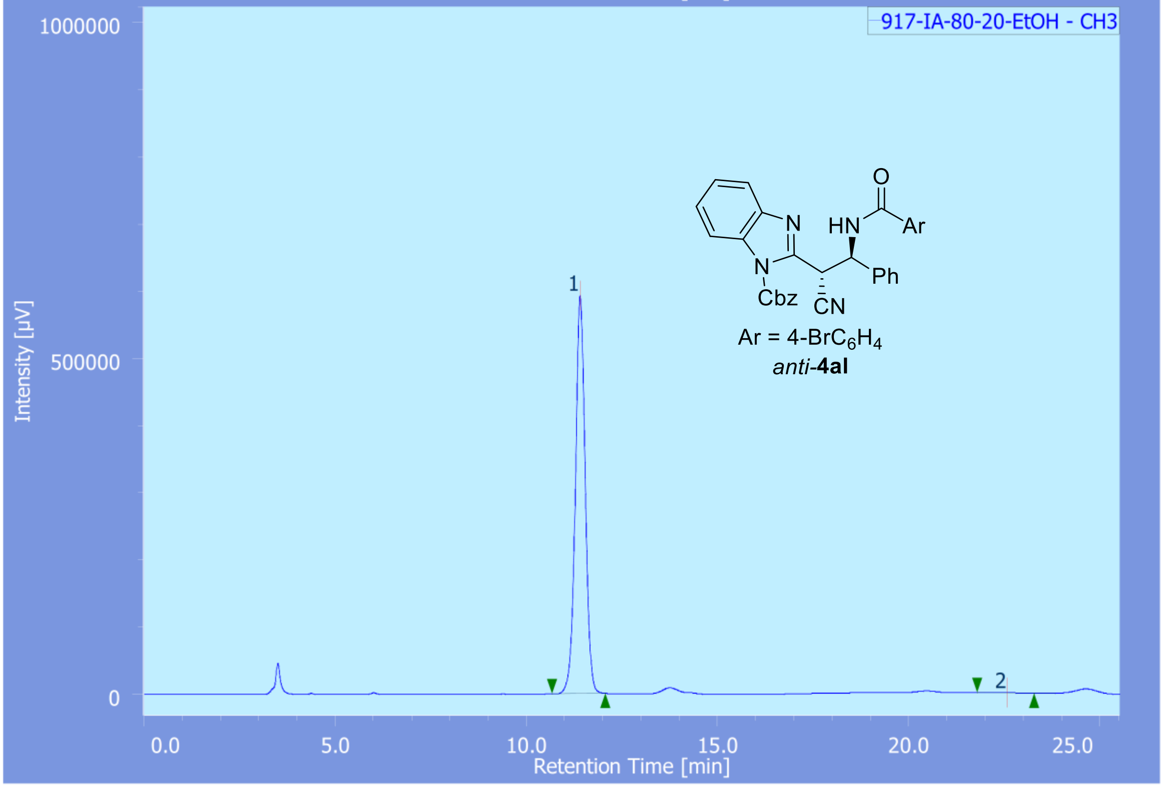

CHIRALPAK IA	Retention time (1)	Retention time (2)	Area (1)	Area (2)	% Area (1)	% Area (2)
(±)-**4al**	11.4	22.6	960868	10640571	49.9	50.1
(1*R*,2*R*)-**4al**	11.4	22.6	963283	7756	99.9	0.1

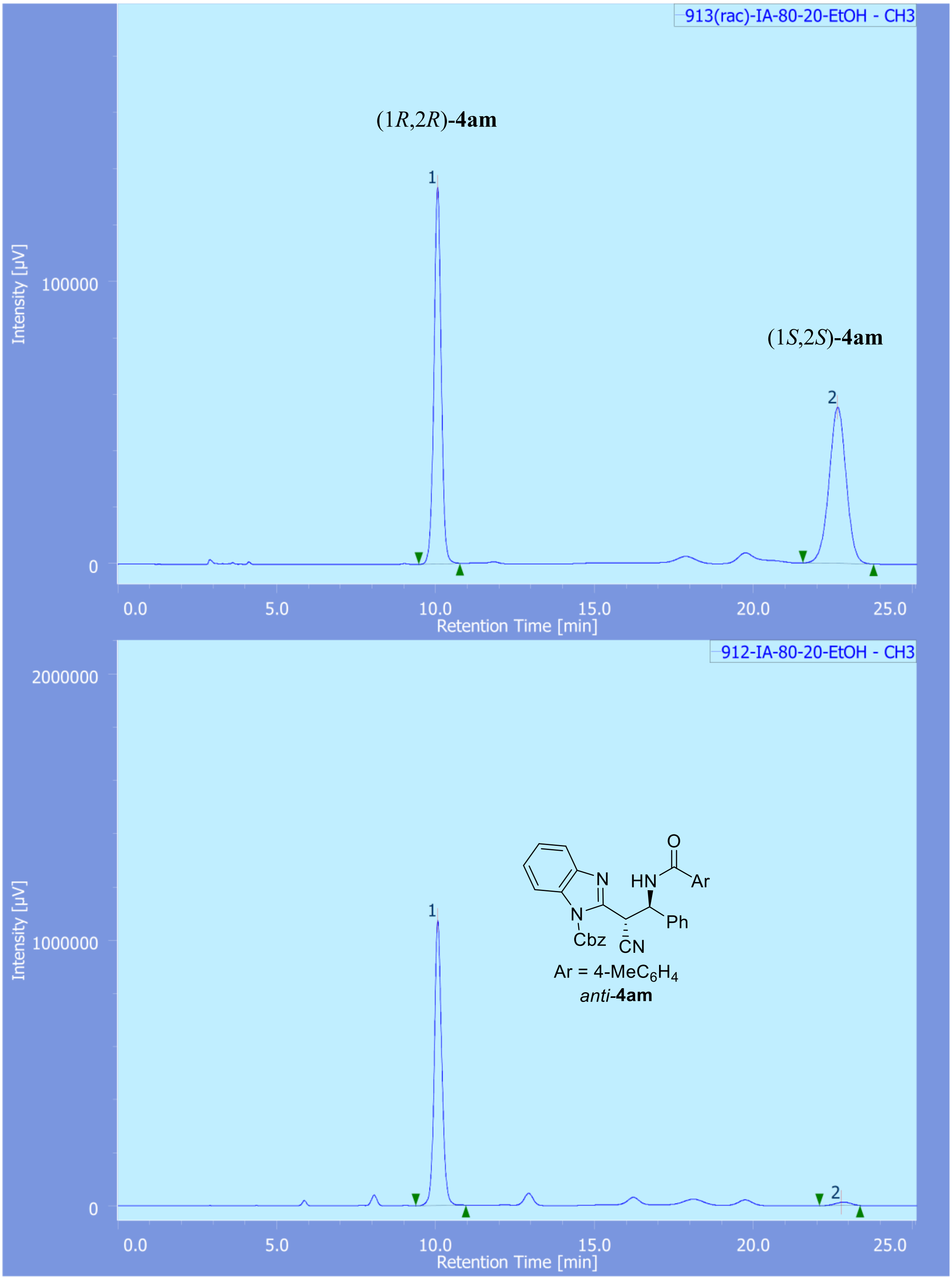

CHIRALPAK IA	Retention time (1)	Retention time (2)	Area (1)	Area (2)	% Area (1)	% Area (2)
(±)-**4am**	11.1	22.7	2122709	2063957	50.7	49.3
(1*R*,2*R*)-**4am**	11.1	22.7	17486527	455023	97.5	2.5

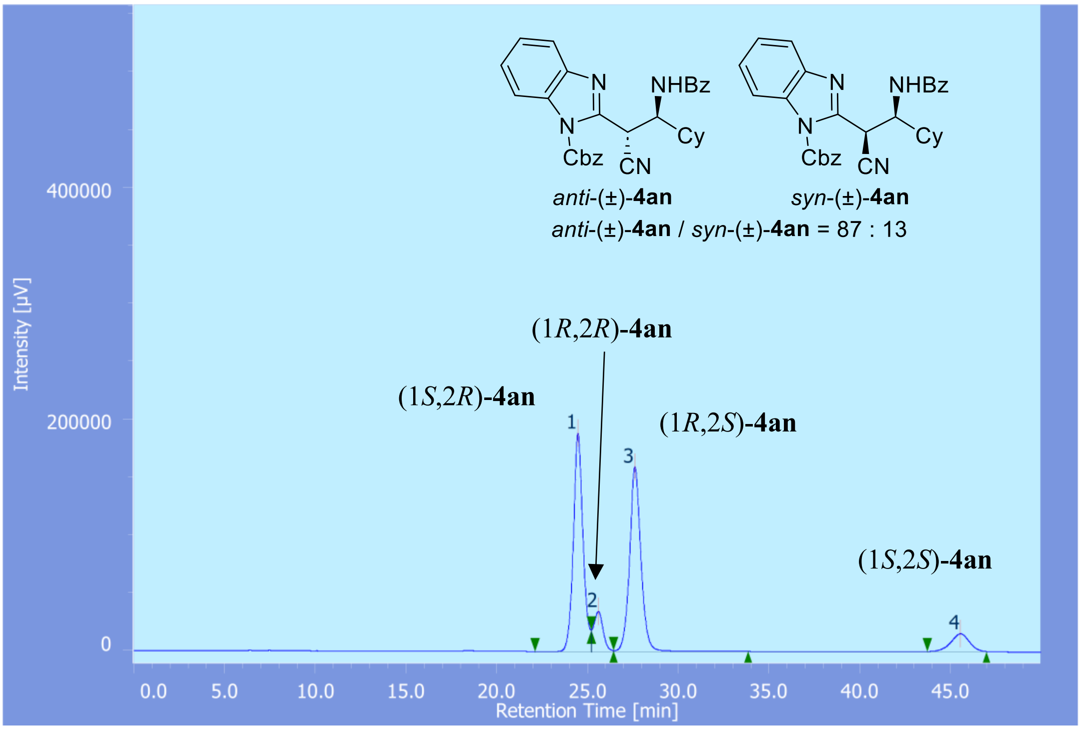

	Retention time	Area	% Area
anti-(1*S*,2*R*)-**4an**	24.5	6971293	42.884
syn-(1*R*,2*R*)-**4an**	25.6	1164271	7.162
anti-(1*R*,2*S*)-**4an**	27.6	6946797	42.733
syn-(1*S*,2*S*)-**4an**	45.5	1173833	7.221

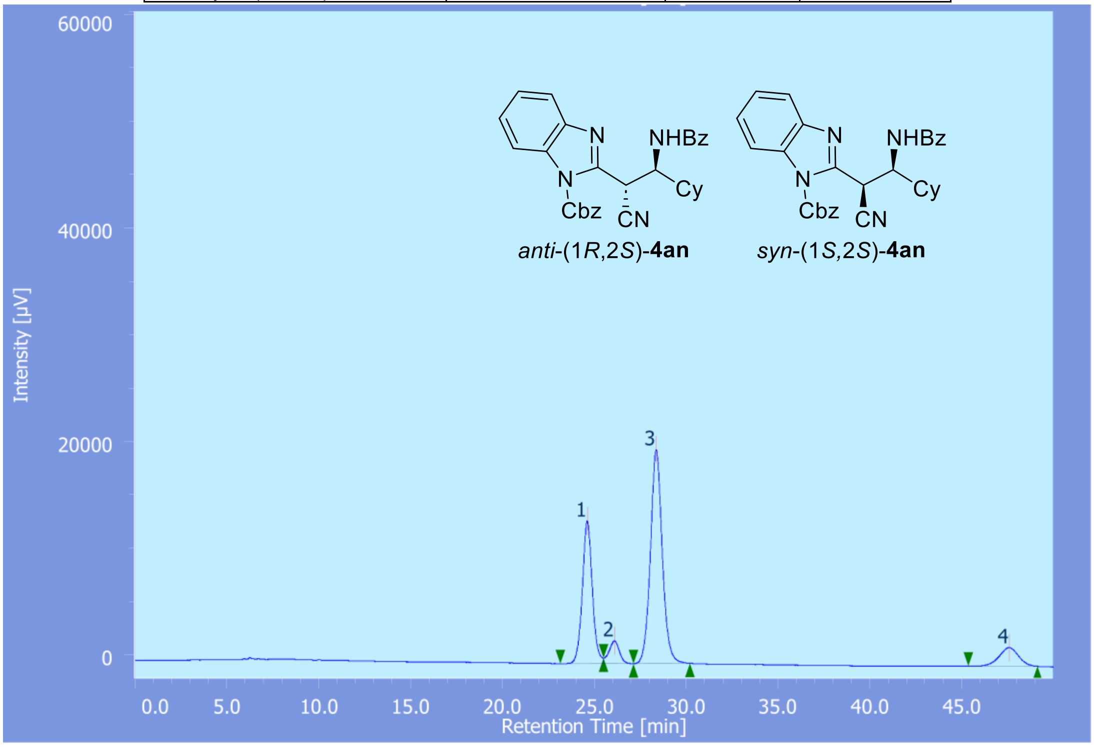

	Retention time	Area	% Area
anti-(1*S*,2*R*)-**4an**	24.6	503830	31.247
syn-(1*R*,2*R*)-**4an**	26.0	82960	5.145
anti-(1*R*,2*S*)-**4an**	28.3	895981	55.568
syn-(1*S*,2*S*)-**4an**	47.6	129620	8.039

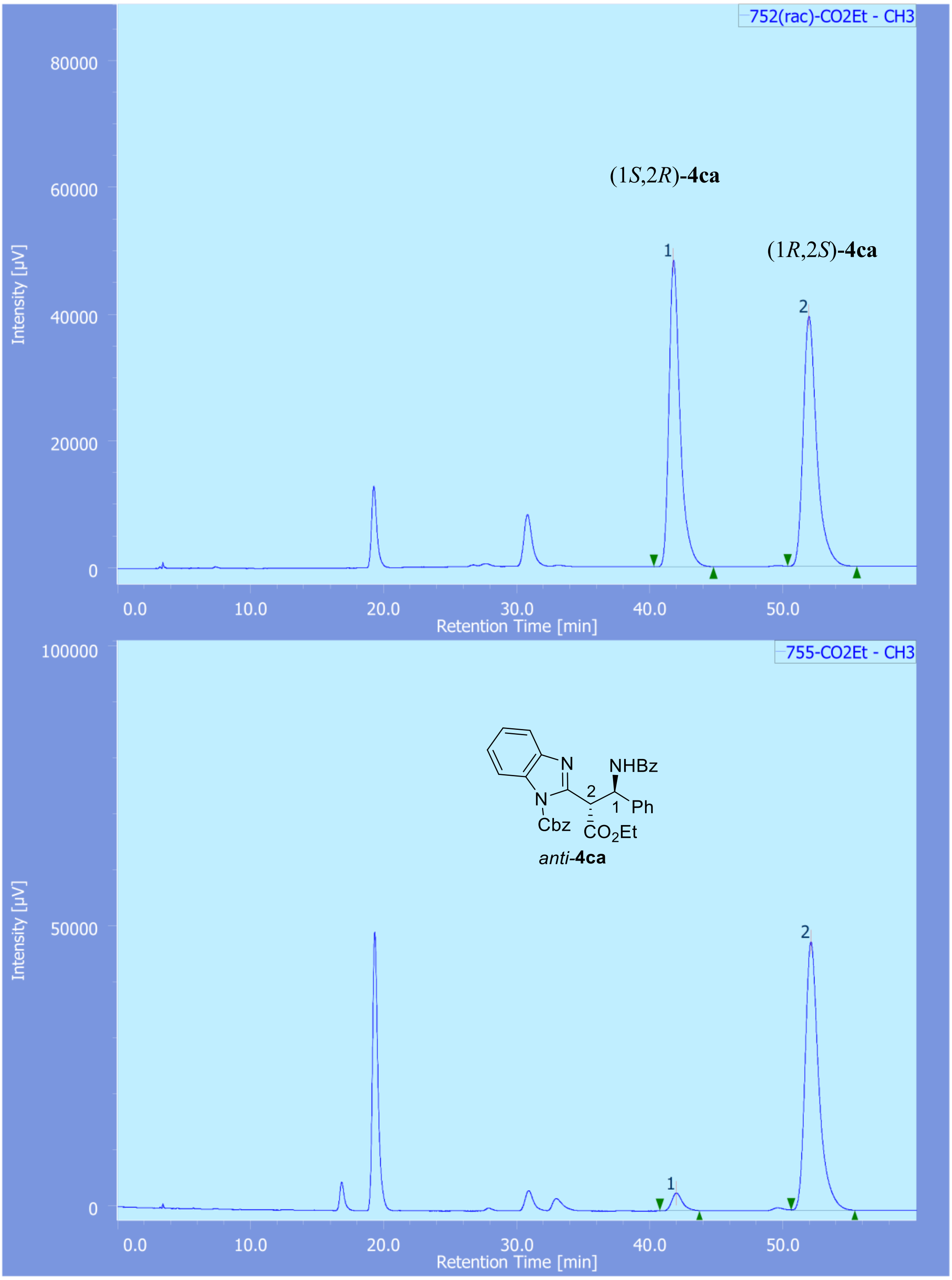

CHIRALPAK IC-3	Retention time (1)	Retention time (2)	Area (1)	Area (2)	% Area (1)	% Area (2)
(±)-**4ca**	42.0	52.1	2798779	2792029	50.1	49.9
(1*R*,2*S*)-**4ca**	42.0	52.1	177234	3402066	4.9	95.1
